# Metal-free C–H arylation of imidazoheterocycles with aryl hydrazines†

**DOI:** 10.1039/c8ra01474d

**Published:** 2018-04-03

**Authors:** Sourav Jana, Sadhanendu Samanta, Avik K. Bagdi, Valerii Z. Shirinian, Alakananda Hajra

**Affiliations:** Department of Chemistry, Visva-Bharati (A Central University) Santiniketan 731235 West Bengal India alakananda.hajra@visva-bharati.ac.in http://www.visvabharati.ac.in/AlakanandaHajra.html; Department of Chemistry, TDB College Raniganj Burdwan 713347 India; Zelinsky Institute of Organic Chemistry RAS 47 Leninsky Prosp. 119991 Moscow Russian Federation

## Abstract

A simple and efficient metal-free arylation of imidazo[1,2-*a*]pyridines at the C-3 position with arylhydrazine has been achieved at room temperature under ambient air conditions. Various 2,3-disubstituted imidazopyridines and imidazothiazoles were synthesized with high yields. The present methodology demonstrates the usefulness of commercially available aryl hydrazine as an arylating agent.

## Abstract 2

Metal-free C–H arylation of imidazo[1,2-*a*]pyridines at C-3 position with arylhydrazines in presence of DBU has been developed at room temperature under ambient air.

## Graphical abstract

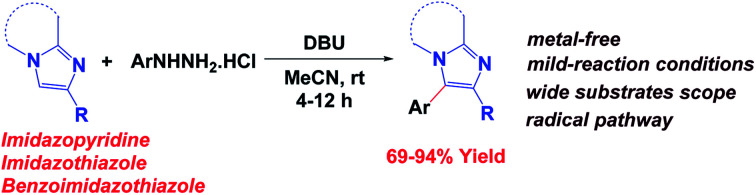

## Introduction

The development of efficient methodologies for the arylation of biological active heterocycles has been of great importance over the years.^1^ Traditionally, arylation is achieved *via* transition-metal-catalyzed cross-coupling reactions.^2^ In the last decade, transition-metal-catalyzed direct C–H arylation has emerged as an alternative to the conventional cross-coupling reaction.^3^ However, use of a metal catalyst, ligand, and additives limits the application of these methodologies. As such, it is desirable to develop transition-metal-free methods for the arylation of bioactive heterocycles.^4^ Arylhydrazine has recently been used as an arylating agent due to its ready availability.^5^ Few methodologies have been developed using arylhydrazine for the arylation of various heterocycles.^5*b*,*d*,*f*,*i*,*j*^

**Fig. 1:**
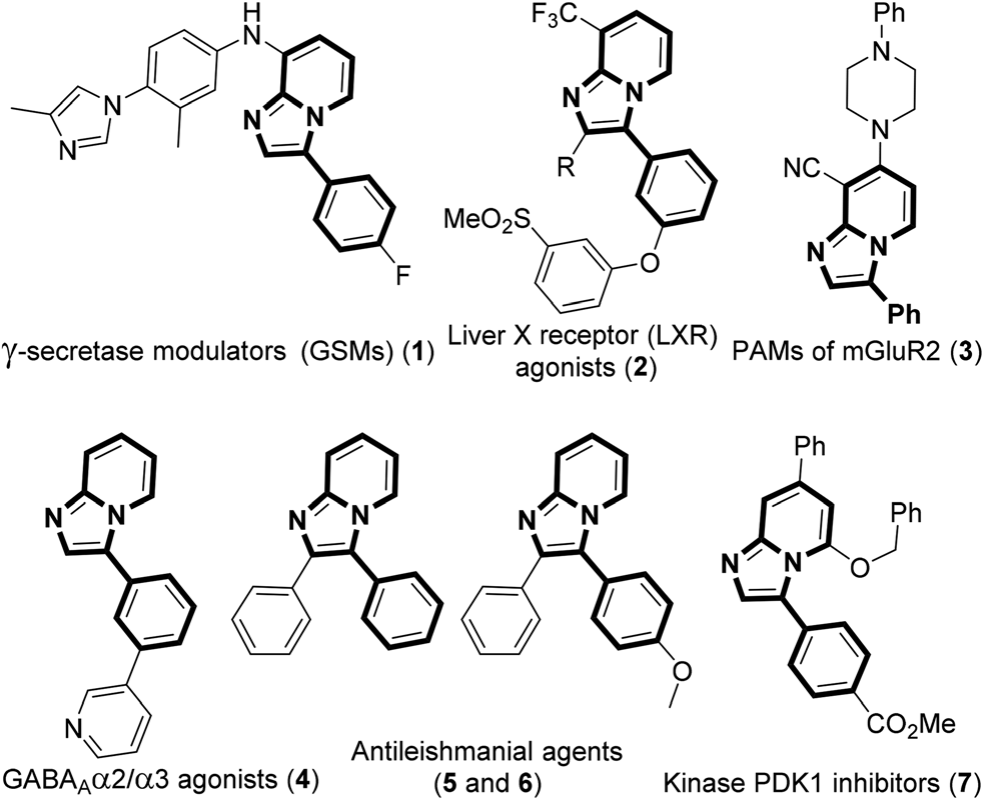
Arylimidazo[1,2-*a*]pyridine containing bioactive molecules.

**Scheme 1:**
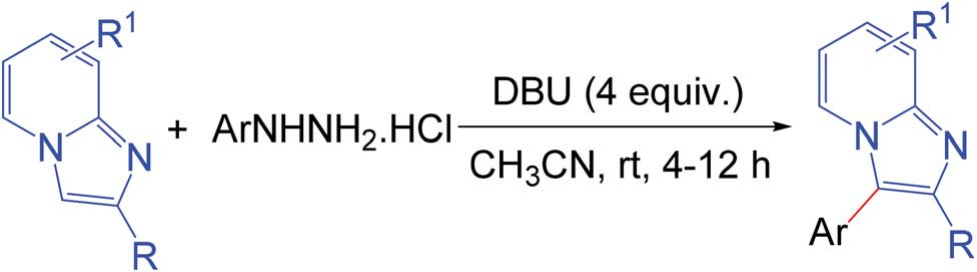
Arylation of imidazopyridines.

Imidazo[1,2-*a*]pyridine has attracted much interest due to its wide range of applications in pharmaceuticals and material science.^6^ The pharmacological activity of this moiety is dependent on its substituents. Several bioactive compounds such as γ-secretase modulators (GSMs) (1), liver X receptor (LXR) agonists (2), positive allosteric modulators (PAMs) of metabotropic glutamate 2 receptor (3), GABA_A_α2/α3 agonists (4), antileishmanial agents (5 and 6), and kinase inhibitors (7) contain the arylimidazo[1,2-*a*]pyridine moiety as the core structure (Fig. 1).^7^ As a consequence, a number of methodologies have been developed for the synthesis and functionalization of this moiety.^8^ Conventionally, arylation of imidazo[1,2-*a*]pyridine is carried out by transition-metal-catalyzed cross-coupling reactions using aryl halide/tosylate/mesylate as the aryl source.^9^ Despite these advances in the functionalization of this moiety, to the best of our knowledge, there is no metal-free protocol for the arylation of imidazo[1,2-*a*]pyridines. This prompted us to develop a transition-metal-free method for the arylation of imidazo[1,2-*a*]pyridines. Herein, we report a direct C–H arylation of imidazo[1,2-*a*]pyridines using easily accessible arylhydrazine hydrochloride in the presence of 1,8-diazabicyclo[5.4.0]undec-7-ene (DBU) at room temperature under ambient air (Scheme 1).

## Results and discussion

We commenced our study by taking 2-phenylimidazo[1,2-*a*]pyridine (1a) and phenyl hydrazine hydrochloride (2a) as the model substrates for the arylation of imidazopyridines. Initially we carried out the reaction using Et_3_N (4 equiv.) as a base in MeCN. Gratifyingly, the expected 2,3-diphenyl imidazo[1,2-*a*]pyridine was obtained in 56% yield within 4 h (Table 1, entry 1). On screening with other organic bases such as Et_2_NH, ^i^Pr_2_NH, DBU and 1,4-diazabicyclo[2.2.2]octane (DABCO), it was found that DBU was the optimal base, affording the desired product in 83% yield (Table 1, entries 2–5). Inorganic bases (K_2_CO_3_, Cs_2_CO_3_, K_3_PO_4_ and KO^*t*^Bu) were also tested, but none were as effective (Table 1, entries 6–9). Other solvents including dimethyl sulfoxide (DMSO), dimethyl formamide (DMF), dimethyl acetamide (DMA), MeNO_2_, dichloroethane (DCE), H_2_O, MeOH and EtOH were not as effective as MeCN (Table 1, entries 10–17). Increment of base loading did not improve the yield, whereas decrement of base loading diminished the yield significantly (Table 1, entries 18 and 19). The reaction did not occur in the absence of base, which suggests a significant role for the base in this arylation reaction (Table 1, entry 20). When the reaction was carried in an oxygen atmosphere, no further improvement in yield was observed (Table 1, entry 21); however, in an inert atmosphere, only a trace amount of the product was obtained (Table 1, entry 22). Thus, the optimum yield was obtained by carrying out the reaction using 4 equiv. of DBU in MeCN in ambient air (Table 1, entry 4).

**Table 1:** Optimization of the reaction conditions^a^

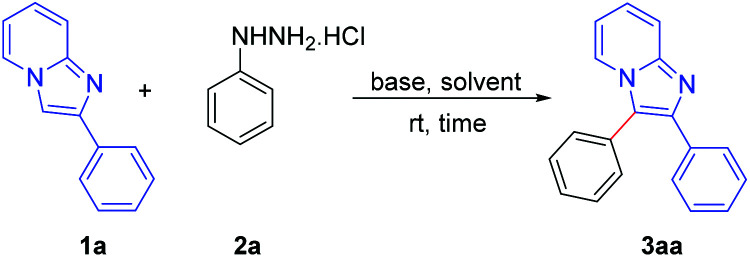				
Entry	Base (equiv.)	Time	Solvent	Yield^b^
1	Et_3_N (4)	4 h	MeCN	56%
2	Et_2_NH (4)	4 h	MeCN	45%
3	^i^Pr_2_NH (4)	4 h	MeCN	35%
4	DBU (4)	4 h	MeCN	83%
5	DABCO (4)	4 h	MeCN	48%
6	K_2_CO_3_ (4)	4 h	MeCN	45%
7	Cs_2_CO_3_ (4)	4 h	MeCN	48%
8	K_3_PO_4_ (4)	4 h	MeCN	39%
9	KO^*t*^Bu (4)	4 h	MeCN	51%
10	DBU (4)	4 h	DMSO	60%
11	DBU (4)	4 h	DMF	40%
12	DBU (4)	4 h	DMA	30%
13	DBU (4)	4 h	MeNO_2_	Trace
14	DBU (4)	4 h	DCE	22%
15	DBU (4)	4 h	H_2_O	NR
16	DBU (4)	4 h	MeOH	54%
17	DBU (4)	4 h	EtOH	41%
18	DBU (5)	4 h	MeCN	83%
19	DBU (3)	4 h	MeCN	55%
20	—	4 h	MeCN	NR^c^
21	DBU (4)	4 h	MeCN	82%^d^
22	DBU (4)	4 h	MeCN	Trace^e^

a Reaction conditions: 0.2 mmol of 1a, 1.3 equiv. of 2a and base in 3 mL solvent at rt.

b Isolated yield.

c NR = no reaction.

d O_2_ atmosphere.

e Ar atmosphere.

**Scheme 2:**
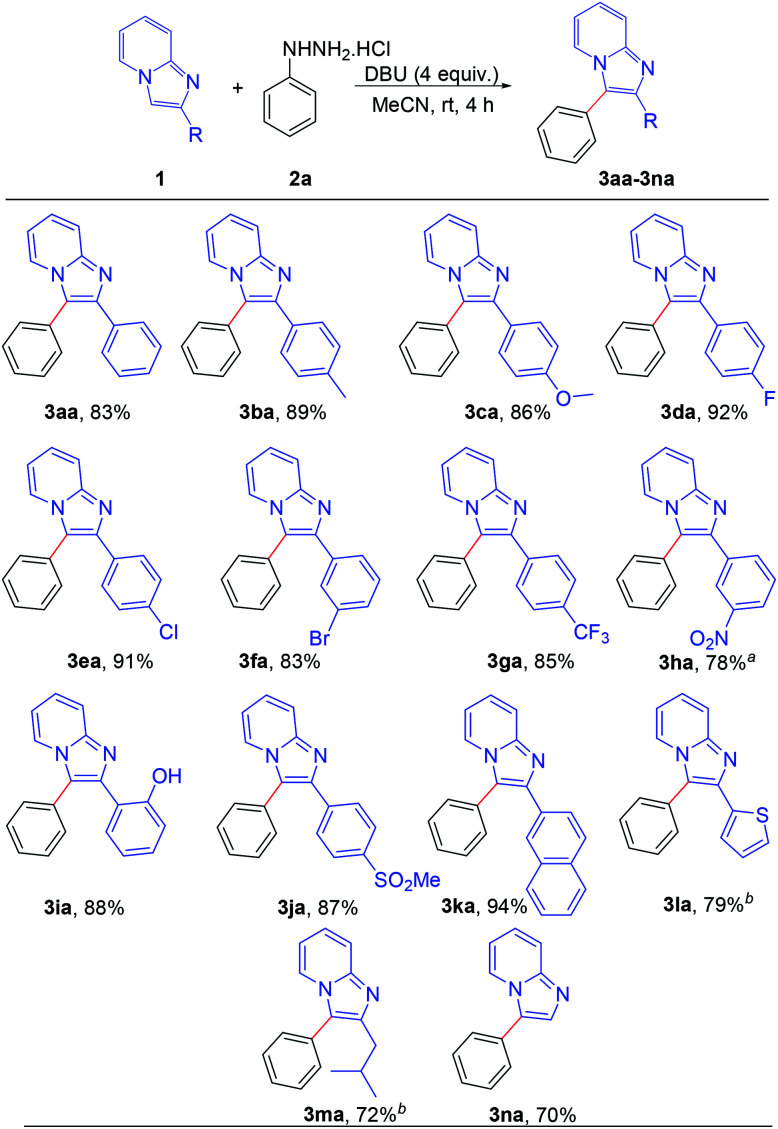
Scope of substrates: variation of C-2 substituents on the imidazo[1,2-*a*]pyridine. Reaction conditions: 0.2 mmol of 1, 1.3 equiv. of 2a and 4 equiv. of DBU in 3 mL MeCN at rt (27 °C) for 4 h. ^a^Reaction completed in 12 h. ^b^Reaction completed in 9 h.

After establishing the optimized reaction conditions, we investigated the substrate scope of this arylation reaction. For this purpose, the effect of the substituent present at the 2-position on the imidazo[1,2-*a*]pyridine moiety was first tested (Scheme 2). Imidazo[1,2-*a*]pyridines bearing a phenyl ring with electron-donating as well as electron-withdrawing functionality afforded the desired products with excellent yields (3aa–3ia). The commercially available drug, zolimidine, was also arylated under the present reaction conditions with 87% yield (3ja). 2-Naphthyl imidazo[1,2-*a*]pyridine and 2-thiophenyl imidazo[1,2-*a*]pyridine also yielded the corresponding products (3ka and 3la). Imidazo[1,2-*a*]pyridine containing an aliphatic substituent at the 2-position effectively reacted with phenyl hydrazine to generate the desired product (3ma). Moreover, unsubstituted imidazo[1,2-*a*]pyridine afforded the regioselectively 3-arylated product with good yield (3na). However, 3-phenylimidazo[1,2-*a*]pyridine did not react under the present reaction conditions. It should also be mentioned that 2,3-diphenylimidazo[1,2-*a*]pyridine (3aa) and 2-(4-methoxyphenyl)-3-phenylimidazo[1,2-*a*]pyridine (3ca) are known as potent antileishmanial agents.^7*d*^

**Scheme 3:**
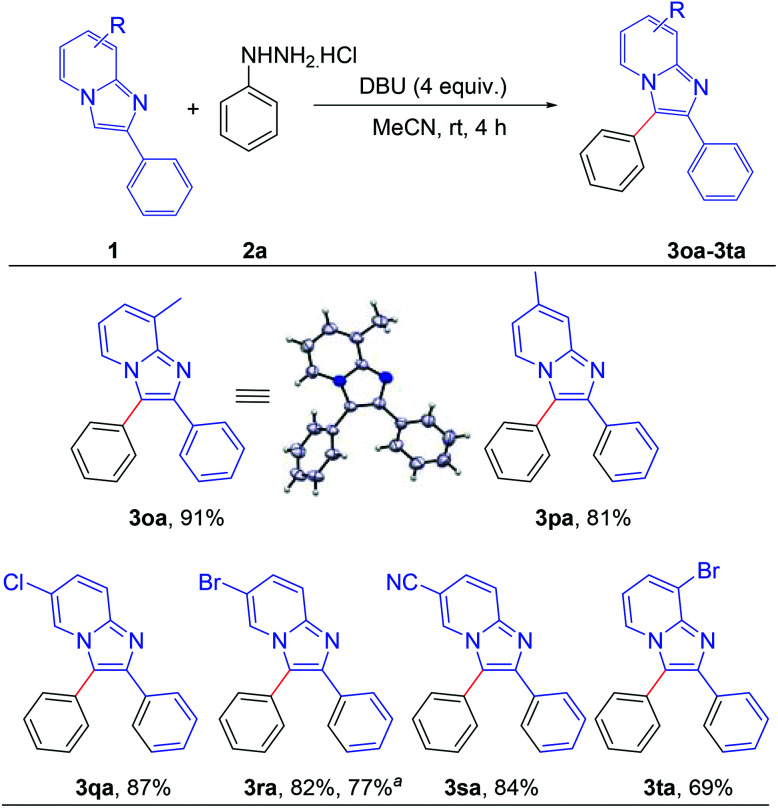
Scope of substrates: variation of substituents on imidazo[1,2-*a*]pyridine at pyridine rings: reaction conditions: 0.2 mmol of 1, 1.3 equiv. of 2a and 4 equiv. of DBU in 3.0 mL MeCN at rt (27 °C) for 4 h. ^a^ Reaction at 5.0 mmol scale.

Next, the effect of the substituent on the pyridine ring of the imidazo[1,2-*a*]pyridine moiety was tested (Scheme 3). Imidazo[1,2-*a*]pyridines bearing different substituents such as –Me, –Cl, –Br and –CN on the pyridine rings successfully afforded the corresponding arylated products with high to excellent yields (3oa–3ta). The structure of 8-methyl-2,3-diphenylimidazo[1,2-*a*]pyridine (3oa) was confirmed by single crystal X-ray crystallography analysis.^10^ Applicability of the present methodology was also demonstrated on a gram scale. 6-Bromo-2,3-diphenylimidazo[1,2-*a*]pyridine reacted efficiently with phenyl hydrazine to afford the product 3ra without significant decrement of the yield (77%).

**Scheme 4:**
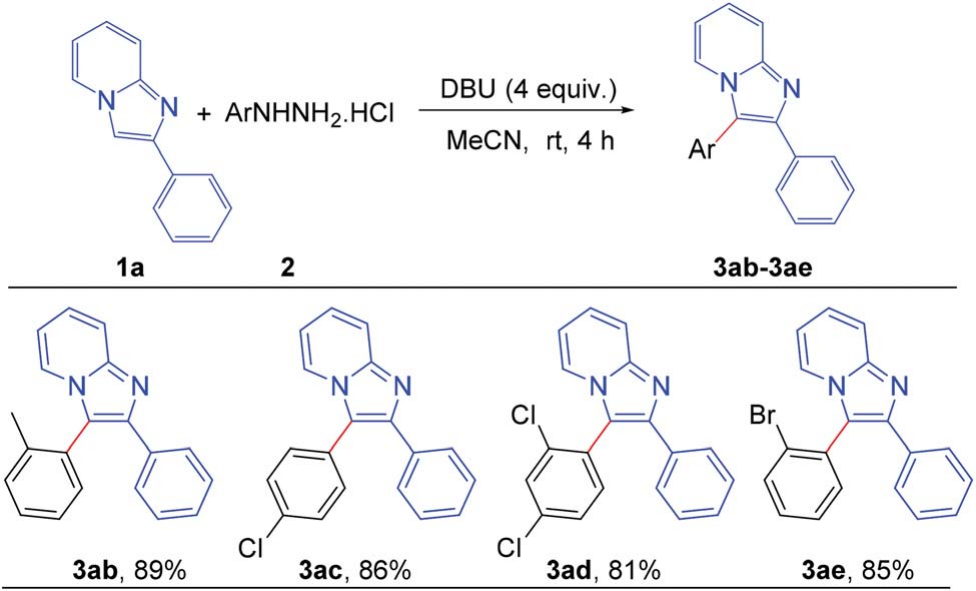
Scope of substrates: variation of substituents on arylhydrazine: reaction conditions: 0.2 mmol of 1a, 1.3 equiv. of 2 and 4 equiv. of DBU in 3.0 mL MeCN at rt (27 °C) for 4 h.

To assess the general applicability of the protocol, different arylhydrazine hydrochlorides were also reacted with 2-phenylimidazo[1,2-*a*]pyridine, as shown in Scheme 4. Different arylhydrazines bearing substituents such as –Me, –Cl, –Br afforded the corresponding arylated products (3ab–3ae) with good to excellent yields. However, arylhydrazines with strong electron-withdrawing groups such as –NO_2_ and –CN were unable to produce the desired products under the present reaction conditions.

**Scheme 5:**
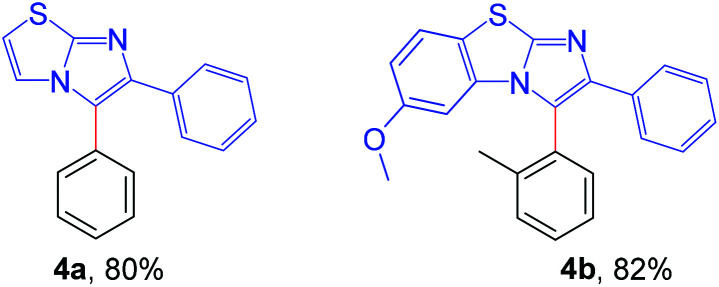
Substrate scope of imidazo[2,1-*b*]thiazole: reaction conditions: 0.2 mmol of imidazo[1,2-*a*]thiazole, 1.3 equiv. of 2 and 4 equiv. of DBU in 3.0 mL MeCN at rt (27 °C) for 4 h.

Next, the applicability of this arylation method was extended to other imidazoheterocycles such as imidazo[1,2-*a*]thiazole and benzo[*d*]imidazo[2,1-*b*]thiazole (Scheme 5). Gratifyingly, this methodology successfully afforded the corresponding arylated products (4a and 4b) without any difficulties.

**Scheme 6:**
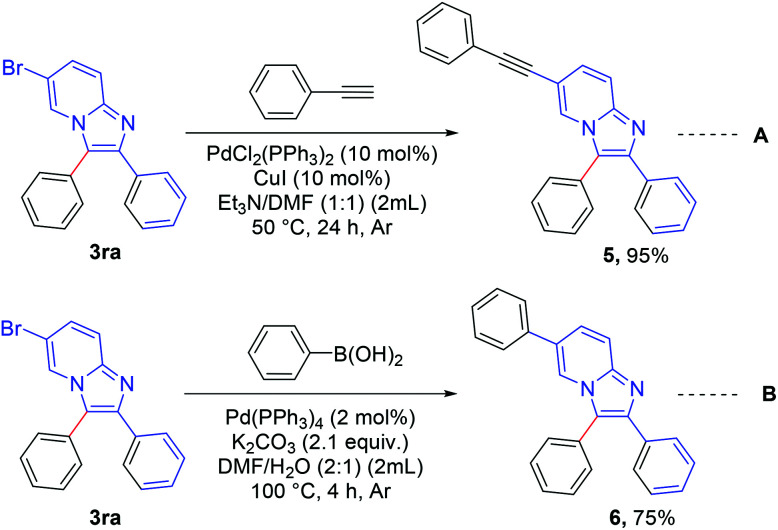
Synthetic application: reaction conditions (A): 0.2 mmol of 3ra and 0.2 mmol of phenyl acetylene with 10 mol% PdCl_2_(PPh_3_)_2_ and 10 mol% Cu i dissolved in 2 mL Et_3_N/DMF (1 : 1) under Ar atmosphere at 50 °C for 24 h. Reaction conditions (B): 0.2 mmol of 3ra and 1 equiv. of phenyl boronic acid with 2 mol% Pd(PPh_3_)_4_ and 2.1 equiv. of K_2_CO_3_ in 2 mL DMF/H_2_O (2 : 1) under Ar atmosphere at 100 °C for 4 h.

The synthesized product was further functionalized employing Sonogashira and Suzuki coupling reactions (Scheme 6).^8*c*^ 6-Bromo-2,3-diphenylimidazo[1,2-*a*]pyridine (3ra) was reacted with ethynylbenzene (Scheme 6, eqn A) to afford 2,3-diphenyl-6-(phenylethynyl)imidazo[1,2-*a*]pyridine (5) in excellent yield (95%). 2,3,6-Triphenylimidazo[1,2-*a*]pyridine (6) was synthesized in good yield (75%) *via* a Suzuki reaction between 6-bromo-2,3-diphenylimidazo[1,2-*a*]pyridine (3ra) and phenyl boronic acid (Scheme 6, eqn B).

**Scheme 7:**
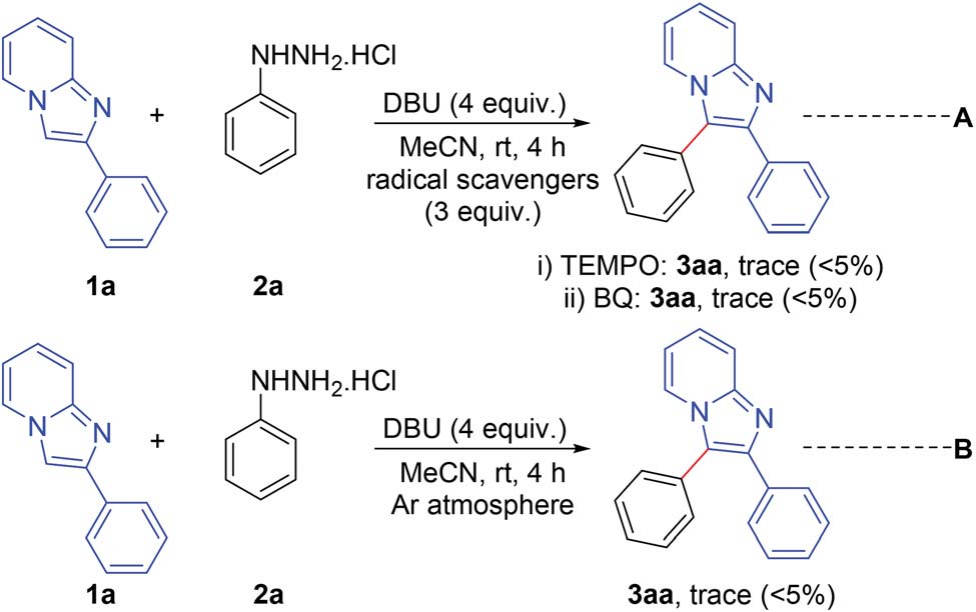
Control experiments.

A number of control experiments were performed to investigate the reaction pathway. The reactions were carried out in the presence of radical scavengers such as 2,2,6,6-tetramethylpiperidine-1-oxyl (TEMPO) and benzo-1,4-quinone (BQ). The formation of a trace amount only of the products indicates that the reaction probably proceeds through a radical pathway (Scheme 7, eqn (A)). Furthermore, the formation of a trace amount of the product in an argon atmosphere suggests that aerial oxygen plays a vital role in this reaction (Scheme 7, eqn (B)).

**Scheme 8:**
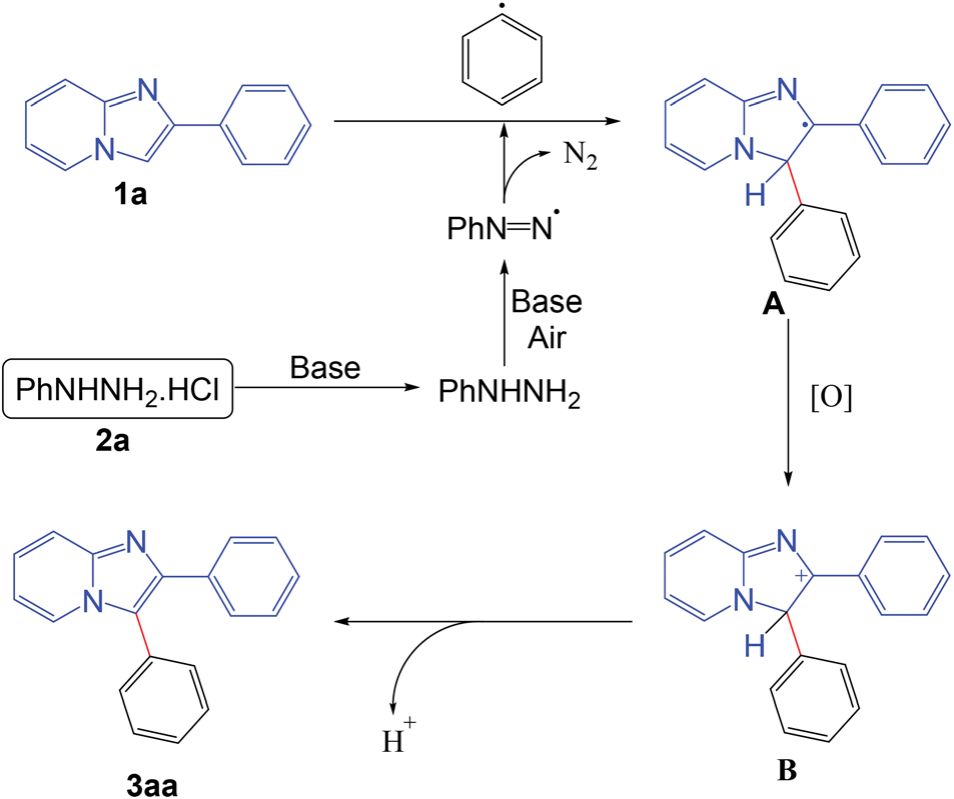
Plausible reaction pathway.

On the basis of the control experiments and literature reports,^5*b*,*f*,*i*^ the probable mechanism of the reaction is outlined in Scheme 8. Initially the phenyl radical is formed from the phenyl hydrazine in the presence of base under aerobic conditions. The generated phenyl radical reacts with the imidazo[1,2-*a*]pyridine moiety to afford the radical intermediate A. Intermediate A is oxidized into the intermediate B under aerobic conditions. Finally, the product is obtained from the intermediate B *via* elimination of a proton.

## Conclusions

In summary, we have developed a metal-free convenient methodology for the arylation of imidazo[1,2-*a*]pyridines employing arylhydrazine as an arylating agent at room temperature. The present methodology offers a practical route for the synthesis of various 2,3-disubstituted imidazo[1,2-*a*]pyridines with a wide range of functional groups. Imidazo[2,1-*b*]thiazole and benzo[*d*]imidazo[2,1-*b*]thiazole were also arylated under the present reaction conditions in good yields. We believe our present protocol for arylation will find useful applications in organic synthesis, the pharmaceutical industry, and material science.

## Experimental section

### General information

Reagents were purchased from commercial sources and used without further purification. ^1^H and ^13^C{^1^H} nuclear magnetic resonance (NMR) spectra were determined on a 400 MHz spectrometer. ^1^H NMR spectra were determined on a 400 MHz spectrometer as solutions in CDCl_3_. Chemical shifts are expressed in parts per million (*δ*) and the signals are reported as s (singlet), d (doublet), t (triplet), m (multiplet), dd (double doublet), and coupling constants (*J*) are given in Hz. ^13^C{^1^H} NMR spectra were recorded at 100 MHz in CDCl_3_ solution. Chemical shifts as internal standard are referenced to CDCl_3_ (*δ* = 7.26 for ^1^H and *δ* = 77.16 for ^13^C{^1^H} NMR) as the internal standard. Thin layer chromatography (TLC) was performed on a silica gel-coated glass slide. Commercially available solvents were freshly distilled before the reaction. All reactions involving moisture-sensitive reactants were executed using oven-dried glassware. X-ray single crystal data were collected using MoKα (*λ* = 0.71073 Å) radiation with Charged Coupled Device (CCD) area. All the imidazoheterocycles were prepared by our reported methods.^8*e*,*f*^

### General experimental procedure for the arylation of imidazo[1,2-*a*]pyridines (3)

A mixture of imidazo[1,2-*a*]pyridine (1, 0.20 mmol, 1 equiv.) and arylhydrazine hydrochloride (2, 0.26 mmol, 1.3 equiv.) was dissolved in 3 mL MeCN at room temperature (rt) in a reaction tube. Then, 4 equiv. of DBU (120 μL) was added to the reaction mixture and stirred in air for 4 h unless otherwise mentioned. After completion of the reaction, the reaction mixture was extracted with ethyl acetate and washed with water. The organic phase was dried over anhydrous Na_2_SO_4_. The crude residue was obtained after evaporating the solvent under reduced pressure and was finally purified by column chromatography on silica gel (60–120 mesh) using petroleum ether and ethylacetate as an eluent to afford the pure product.

#### 2,3-Diphenylimidazo[1,2-*a*]pyridine (3aa):^7*d*,8*e*^

White solid (45 mg, 83%), mp: 149–150 °C (lit. 149–150 °C); ^1^H NMR (CDCl_3_, 400 MHz): *δ* 7.96 (d, *J* = 6.8 Hz, 1H), 7.70–7.65 (m, 3H), 7.55–7.44 (m, 5H), 7.28–7.19 (m, 4H), 6.76–6.72 (m, 1H); ^13^C{^1^H} NMR (CDCl_3_, 100 MHz): *δ* 144.9, 142.5, 134.2, 130.8, 130.0, 129.7, 129.0, 128.4, 128.2, 127.6, 124.8, 123.4, 121.2, 117.6, 112.4.

#### 3-Phenyl-2-(*p*-tolyl)imidazo[1,2-*a*]pyridine (3ba)

White solid (51 mg, 89%), mp: 97–99 °C; ^1^H NMR (CDCl_3_, 400 MHz): *δ* 7.95 (d, *J* = 6.8 Hz, 1H), 7.68 (d, *J* = 9.2 Hz, 1H), 7.56–7.44 (m, 7H), 7.21–7.17 (m, 1H), 7.09 (d, *J* = 8.0 Hz, 2H), 6.72 (t, *J* = 6.8 Hz, 1H), 2.32 (s, 3H); ^13^C{^1^H} NMR (CDCl_3_, 100 MHz): *δ* 144.8, 142.5, 137.4, 131.3, 130.8, 130.1, 129.6, 129.1, 128.9, 128.1, 124.7, 123.3, 120.8, 117.5, 112.3, 21.3; anal. calcd for C_20_H_16_N_2_: C, 84.48; H, 5.67; N, 9.85; found C, 84.31; H, 5.78; N, 9.91%.

#### 2-(4-Methoxyphenyl)-3-phenylimidazo[1,2-*a*]pyridine (3ca):^7*d*^

White solid (52 mg, 86%), mp: 134–135 °C (lit. Mp 135–136 °C); ^1^H NMR (CDCl_3_, 400 MHz): *δ* 7.94 (d, *J* = 7.2 Hz, 1H), 7.67 (d, *J* = 8.8 Hz, 1H), 7.61–7.57 (m, 2H), 7.55–7.44 (m, 5H), 7.21–7.17 (m, 1H), 6.83–6.81 (m, 2H), 6.72 (t, *J* = 6.8 Hz, 1H), 3.79 (s, 3H); ^13^C{^1^H} NMR (CDCl_3_, 100 MHz): *δ* 159.3, 144.8, 130.9, 130.1, 129.6, 129.4, 129.2, 128.9, 126.7, 124.7, 123.3, 120.4, 117.4, 113.8, 112.3, 55.3.

#### 2-(4-Fluorophenyl)-3-phenylimidazo[1,2-*a*]pyridine (3da):^7*d*^

White solid (53 mg, 92%), mp: 104 °C; ^1^H NMR (CDCl_3_, 400 MHz): *δ* 7.95 (d, *J* = 6.8 Hz, 1H), 7.67–7.61 (m, 3H), 7.55–7.42 (m, 5H), 7.22–7.18 (m, 1H), 6.99–6.93 (m, 2H), 6.74 (t, *J* = 7.6 Hz, 1H); ^13^C{^1^H} NMR (CDCl_3_, 100 MHz): *δ* 162.5 (d, *J*_C–F_ = 245 Hz), 144.9, 141.7, 130.8, 130.4 (d, *J*_C–F_ = 3 Hz), 129.8 (d, *J*_C–F_ = 9 Hz), 129.7, 129.1, 124.9, 123.4, 120.9, 117.6, 115.7, 115.3 (d, *J*_C–F_ = 22 Hz), 112.5.

#### 2-(4-Chlorophenyl)-3-phenylimidazo[1,2-*a*]pyridine (3ea)

Yellow semi solid (55 mg, 91%); ^1^H NMR (CDCl_3_, 400 MHz): *δ* 7.97 (d, *J* = 7.2 Hz, 1H), 7.69 (d, *J* = 9.2 Hz, 1H), 7.62–7.60 (m, 2H), 7.56–7.52 (m, 3H), 7.47–7.45 (m, 2H), 7.28–7.21 (m, 3H), 6.79–6.75 (m, 1H); ^13^C{^1^H} NMR (CDCl_3_, 100 MHz): *δ* 145.0, 141.4, 133.4, 132.8, 130.8, 129.8, 129.7, 129.4, 129.2, 128.6, 125.0, 123.4, 121.3, 117.7, 112.5; anal. calcd for C_19_H_13_ClN_2_: C, 74.88; H, 4.30; N, 9.19; found C, 75.09; H, 4.40; N, 9.01%.

#### 2-(3-Bromophenyl)-3-phenylimidazo[1,2-*a*]pyridine (3fa)

Yellow solid (58 mg, 83%), mp: 122–123 °C; ^1^H NMR (CDCl_3_, 400 MHz): *δ* 7.95–7.91 (m, 2H), 7.67 (d, *J* = 9.2 Hz, 1H), 7.57–7.43 (m, 6H), 7.36 (d, *J* = 8.8 Hz, 1H), 7.24–7.20 (m, 1H), 7.10 (t, *J* = 8.0 Hz, 1H), 6.75 (t, *J* = 6.8 Hz, 1H); ^13^C{^1^H} NMR (CDCl_3_, 100 MHz): *δ* 144.9, 140.9, 136.4, 131.0, 130.8, 130.5, 129.8, 129.4, 129.3, 129.2, 126.5, 125.1, 123.5, 122.6, 121.7, 117.7, 112.6; anal. calcd for C_19_H_13_BrN_2_: C, 65.35; H, 3.75; N, 8.02; found C, 65.52; H, 3.83; N, 8.09%.

#### 3-Phenyl-2-(4-(trifluoromethyl)phenyl)imidazo[1,2-*a*]pyridine (3ga)

White solid (58 mg, 85%), mp: 67–68 °C; ^1^H NMR (CDCl_3_, 400 MHz): *δ* 7.94 (d, *J* = 6.8 Hz, 1H), 7.77 (d, *J* = 8.0 Hz, 2H), 7.69 (d, *J* = 9.2 Hz, 1H), 7.58–7.51 (m, 5H), 7.45–7.43 (m, 2H), 7.25–7.21 (m, 1H), 6.78–6.74 (m, 1H); ^13^C{^1^H} NMR (CDCl_3_, 100 MHz): *δ* 145.0, 140.9, 137.9, 130.8, 130.0, 129.9, 129.4, 129.0 (q, *J*_C–F_ = 37 Hz), 128.2, 127.1 (q, *J*_C–F_ = 270 Hz), 126.2, 125.3 (q, *J*_C–F_ = 6 Hz), 123.5, 122.1, 117.8, 112.7; HRMS (ESI-TOF) *m*/*z*: [M + H]^+^ calcd for C_20_H_14_F_3_N_2_: 339.1104; found: 339.1107.

#### 2-(3-Nitrophenyl)-3-phenylimidazo[1,2-*a*]pyridine (3ha)

Brown solid (49 mg, 78%), mp: 147–148 °C; ^1^H NMR (CDCl_3_, 400 MHz): *δ* 8.32 (t, *J* = 2.0 Hz, 1H), 7.88–7.86 (m, 1H), 7.78–7.73 (m, 2H), 7.49 (d, *J* = 9.2 Hz, 1H), 7.39–7.33 (m, 3H), 7.26–7.20 (m, 3H), 7.07–7.03 (m, 1H), 6.60–6.56 (m, 1H); ^13^C{^1^H} NMR (CDCl_3_, 100 MHz): *δ* 148.5, 145.0, 140.0, 136.2, 133.7, 131.1, 130.7, 130.0, 129.7, 129.3, 129.0, 125.6, 123.7, 122.8, 122.2, 117.8, 112.9; anal. calcd for C_19_H_13_N_3_O_2_: C, 72.37; H, 4.16; N, 13.33; found C, 72.51; H, 4.07; N, 13.24%.

#### 2-(3-Phenylimidazo[1,2-*a*]pyridin-2-yl)phenol (3ia)

White solid (50 mg, 88%), mp: 84–86 °C; ^1^H NMR (CDCl_3_, 400 MHz): *δ* 13.04 (brs, 1H), 7.84 (d, *J* = 6.8 Hz, 1H), 7.65–7.55 (m, 4H), 7.50–7.48 (m, 2H), 7.28–7.24 (m, 1H), 7.16–7.12 (m, 2H), 7.03 (d, *J* = 8.4 Hz, 1H), 6.81–6.77 (m, 1H), 6.58–6.54 (m, 1H); ^13^C{^1^H} NMR (CDCl_3_, 100 MHz): *δ* 157.9, 142.5, 140.9, 131.3, 130.0, 129.8, 129.7, 129.6, 129.4, 127.2, 125.4, 123.2, 118.5, 117.7, 116.9, 116.7, 113.0; HRMS (ESI-TOF) *m*/*z*: [M + H]^+^ calcd for C_19_H_15_N_2_O: 287.1179; found: 287.1179.

#### 2-(4-(Methylsulfonyl)phenyl)-3-phenylimidazo[1,2-*a*]pyridine (3ja)

Yellow liquid (61 mg, 87%); ^1^H NMR (CDCl_3_, 400 MHz): *δ* 7.94 (d, *J* = 6.8 Hz, 1H), 7.87–7.81 (m, 4H), 7.70 (d, *J* = 9.2 Hz, 1H), 7.59–7.54 (m, 3H), 7.45–7.43 (m, 2H), 7.244–7.241 (m, 1H), 6.80–6.77 (m, 1H), 3.04 (s, 3H); ^13^C{^1^H} NMR (CDCl_3_, 100 MHz): *δ* 145.1, 140.0, 138.9, 130.7, 130.0, 129.6, 128.6, 128.0, 127.5, 127.3, 126.7, 125.6, 123.6, 117.9, 113.0, 44.6; HRMS (ESI-TOF) *m*/*z*: [M + H]^+^ calcd for C_20_H_17_N_2_O_2_S: 349.1005; found: 349.0998.

#### 2-(Naphthalen-2-yl)-3-phenylimidazo[1,2-*a*]pyridine (3ka)

White solid (60 mg, 94%), mp: 117–118 °C; ^1^H NMR (CDCl_3_, 400 MHz): *δ* 8.25 (s, 1H), 8.00 (d, *J* = 7.2 Hz, 1H), 7.78–7.67 (m, 5H), 7.54–7.48 (m, 5H), 7.44–7.40 (m, 2H), 7.25–7.21 (m, 1H), 6.77–6.74 (m, 1H); ^13^C{^1^H} NMR (CDCl_3_, 100 MHz): *δ* 145.1, 142.4, 133.6, 132.9, 131.7, 130.9, 130.0, 129.7, 129.1, 128.5, 127.8, 127.6, 127.3, 126.1, 126.09, 126.06, 124.9, 123.4, 121.6, 117.6, 112.5; HRMS (ESI-TOF) *m*/*z*: [M + H]^+^ calcd for C_23_H_17_N_2_: 321.1386; found: 321.1386.

#### 3-Phenyl-2-(thiophen-2-yl)imidazo[1,2-*a*]pyridine (3la)

White solid (44 mg, 79%), mp: 65–67 °C; ^1^H NMR (CDCl_3_, 400 MHz): *δ* 7.83 (d, *J* = 6.8 Hz, 1H), 7.65 (d, *J* = 9.2 Hz, 1H), 7.59–7.50 (m, 5H), 7.22–7.16 (m, 2H), 7.09 (dd, *J* = 3.6 Hz, 0.8 Hz, 1H), 6.93–6.91 (m, 1H), 6.71 (t, *J* = 6.8 Hz, 1H); ^13^C{^1^H} NMR (CDCl_3_, 100 MHz): *δ* 144.7, 137.7, 137.6, 131.1, 129.7, 129.5, 129.1, 127.5, 125.3, 125.0, 124.6, 123.3, 120.0, 117.3, 112.5; anal. calcd for C_17_H_12_N_2_S: C, 73.89; H, 4.38; N, 10.14; found C, 74.13; H, 4.31; N, 10.02%.

#### 2-Isobutyl-3-phenylimidazo[1,2-*a*]pyridine (3ma)

Yellow liquid (36 mg, 72%); ^1^H NMR (CDCl_3_, 400 MHz): *δ* 8.00 (d, *J* = 7.2 Hz, 1H), 7.58 (d, *J* = 8.8 Hz, 1H), 7.54–7.51 (m, 2H), 7.45–7.41 (m, 3H), 7.16–7.12 (m, 1H), 6.71–6.68 (m, 1H), 2.64 (d, *J* = 7.2 Hz, 2H), 2.25–2.14 (m, 1H), 0.89 (d, *J* = 6.8 Hz, 6H); ^13^C{^1^H} NMR (CDCl_3_, 100 MHz): *δ* 144.6, 130.1, 129.7, 129.3, 128.9, 128.3, 126.8, 124.1, 123.2, 117.2, 111.8, 36.9, 29.1, 22.7; anal. calcd for C_17_H_18_N_2_: C, 81.56; H, 7.25; N, 11.19; found C, 81.21; H, 7.41; N, 11.38%.

#### 3-Phenylimidazo[1,2-*a*]pyridine (3na)^9*d*^

Colorless liquid (27 mg, 70%); ^1^H NMR (CDCl_3_, 400 MHz): *δ* 8.33 (d, *J* = 6.8 Hz, 1H), 7.69 (s, 1H), 7.67 (d, *J* = 8.8 Hz, 1H), 7.57–7.49 (m, 4H), 7.43–7.38 (m, 1H), 7.21–7.16 (m, 1H), 6.81–6.78 (m, 1H); ^13^C{^1^H} NMR (CDCl_3_, 100 MHz): *δ* 146.2, 132.6, 129.4, 129.3, 128.3, 128.1, 125.8, 124.3, 123.4, 118.3, 112.6.

#### 8-Methyl-2,3-diphenylimidazo[1,2-*a*]pyridine (3oa)^8*d*^

White solid (52 mg, 91%), mp: 104–106 °C; ^1^H NMR (CDCl_3_, 400 MHz): *δ* 7.82 (d, *J* = 6.8 Hz, 1H), 7.67–7.64 (m, 2H), 7.51–7.41 (m, 5H), 7.28–7.19 (m, 3H), 6.98–6.96 (m, 1H), 6.63 (t, *J* = 6.8 Hz, 1H), 2.69 (s, 3H); ^13^C{^1^H} NMR (CDCl_3_, 100 MHz): *δ* 145.4, 142.1, 134.6, 130.8, 130.3, 129.5, 128.8, 128.4, 128.3, 127.6, 127.4, 123.5, 121.6, 121.3, 112.4, 17.3.

#### 7-Methyl-2,3-diphenylimidazo[1,2-*a*]pyridine (3pa)^8*e*^

White solid (46 mg, 81%), mp: 131–133 °C; ^1^H NMR (CDCl_3_, 400 MHz): *δ* 7.84 (d, *J* = 6.8 Hz, 1H), 7.66–7.63 (m, 2H), 7.53–7.42 (m, 6H), 7.29–7.22 (m, 3H), 6.56 (dd, *J* = 7.2 Hz, 1.6 Hz, 1H), 2.41 (s, 3H); ^13^C{^1^H} NMR (CDCl_3_, 100 MHz): *δ* 145.3, 142.0, 135.9, 134.3, 130.8, 130.1, 129.6, 128.8, 128.3, 128.1, 127.4, 122.6, 120.6, 115.9, 115.1, 21.4.

#### 6-Chloro-2,3-diphenylimidazo[1,2-*a*]pyridine (3qa)

Yellow solid (53 mg, 87%), mp: 142–144 °C; ^1^H NMR (CDCl_3_, 400 MHz): *δ* 7.968–7.961 (m, 1H), 7.65–7.61 (m, 3H), 7.58–7.51 (m, 3H), 7.45–7.43 (m, 2H), 7.30–7.25 (m, 3H), 7.16 (dd, *J* = 9.6 Hz, 2.0 Hz, 1H); ^13^C{^1^H} NMR (CDCl_3_, 100 MHz): *δ* 143.5, 143.2, 133.8, 130.7, 129.8, 129.4, 129.3, 128.4, 128.1, 127.9, 126.1, 121.2, 120.7, 118.0, 115.6; anal. calcd for C_19_H_13_ClN_2_: C, 74.88; H, 4.30; N, 9.19; found C, 75.06; H, 4.36; N, 9.26%.

#### 6-Bromo-2,3-diphenylimidazo[1,2-*a*]pyridine (3ra)

White solid (57 mg, 82%), mp: 201–202 °C; ^1^H NMR (CDCl_3_, 400 MHz): *δ* 8.06 (s, 1H), 7.65–7.63 (m, 2H), 7.59–7.52 (m, 4H), 7.46–7.44 (m, 2H), 7.29–7.25 (m, 4H); ^13^C{^1^H} NMR (CDCl_3_, 100 MHz): *δ* 143.3, 142.0, 133.8, 130.7, 129.9, 129.4, 129.3, 128.4, 128.3, 128.1, 127.9, 123.4, 121.6, 118.3, 107.2; anal. calcd for C_19_H_13_BrN_2_: C, 65.35; H, 3.75; N, 8.02; found C, 65.18; H, 3.67; N, 8.11%.

#### 2,3-Diphenylimidazo[1,2-*a*]pyridine-6-carbonitrile (3sa)

White solid (50 mg, 84%), mp: 151–153 °C; ^1^H NMR (CDCl_3_, 400 MHz): *δ* 8.34 (s, 1H), 7.74 (d, *J* = 10.0 Hz, 1H), 7.68–7.65 (m, 2H), 7.62–7.56 (m, 3H), 7.45–7.43 (m, 2H), 7.32–7.28 (m, 4H); ^13^C{^1^H} NMR (CDCl_3_, 100 MHz): *δ* 144.7, 143.9, 133.0, 130.7, 130.1, 130.0, 129.7, 128.6, 128.5, 128.3, 128.2, 124.4, 122.2, 118.5, 116.9, 98.6; HRMS (ESI-TOF) *m*/*z*: [M + H]^+^ calcd for C_20_H_14_N_3_: 296.1182; found: 296.1166.

#### 8-Bromo-2,3-diphenylimidazo[1,2-*a*]pyridine (3ta)

White solid (48 mg, 69%), mp: 164–167 °C; ^1^H NMR (CDCl_3_, 400 MHz): *δ* 7.93 (dd, *J* = 6.8 Hz, 0.8 Hz, 1H), 7.70–7.67 (m, 2H), 7.55–7.42 (m, 7H), 7.30–7.26 (m, 2H), 6.62 (t, *J* = 7.2 Hz, 1H); ^13^C{^1^H} NMR (CDCl_3_, 100 MHz): *δ* 152.7, 146.9, 134.0, 133.8, 130.8, 129.7, 129.3, 128.53, 128.50, 128.4, 127.8, 127.1, 122.8, 113.0, 112.3; anal. calcd for C_19_H_13_BrN_2_: C, 65.35; H, 3.75; N, 8.02; found C, 65.49; H, 3.68; N, 7.94%.

#### 2-Phenyl-3-(*o*-tolyl)imidazo[1,2-*a*]pyridine (3ab)^7*e*^

White solid (51 mg, 89%), mp: 124–126 °C (lit. 124–126 °C); ^1^H NMR (CDCl_3_, 400 MHz): *δ* 7.71–7.65 (m, 3H), 7.56 (d, *J* = 6.8 Hz, 1H), 7.45–7.43 (m, 2H), 7.36–7.34 (m, 2H), 7.26–7.18 (m, 4H), 6.74–6.70 (m, 1H), 2.01 (s, 3H); ^13^C{^1^H} NMR (CDCl_3_, 100 MHz): *δ* 144.9, 139.3, 132.4, 131.9, 131.1, 129.8, 129.3, 129.0, 128.5, 127.5, 127.3, 127.1, 124.6, 123.5, 120.1, 117.5, 112.3, 19.5.

#### 3-(4-Chlorophenyl)-2-phenylimidazo[1,2-*a*]pyridine (3ac)^9*f*^

White solid (52 mg, 86%), mp: 171–173 °C (lit. 173–175 °C); ^1^H NMR (CDCl_3_, 400 MHz): *δ* 7.94 (d, *J* = 7.2 Hz, 1H), 7.68 (d, *J* = 9.2 Hz, 1H), 7.64–7.62 (m, 2H), 7.50 (d, *J* = 8.4 Hz, 2H), 7.39 (d, *J* = 8.8 Hz, 2H), 7.32–7.20 (m, 4H), 6.76 (t, *J* = 6.8 Hz, 1H); ^13^C{^1^H} NMR (CDCl_3_, 100 MHz): *δ* 145.1, 142.9, 135.0, 133.9, 132.1, 130.0, 129.4, 128.5, 128.3, 127.8, 125.1, 123.2, 117.7, 117.1, 112.7.

#### 3-(2,4-Dichlorophenyl)-2-phenylimidazo[1,2-*a*]pyridine (3ad)

Yellow liquid (55 mg, 81%); ^1^H NMR (CDCl_3_, 400 MHz): *δ* 7.72 (d, *J* = 8.8 Hz, 1H), 7.67 (d, *J* = 2.0 Hz, 1H), 7.61–7.59 (m, 3H), 7.37 (dd, *J* = 8.0 Hz, 2.0 Hz, 1H), 7.32–7.26 (m, 5H), 6.79 (t, *J* = 6.8 Hz, 1H); ^13^C{^1^H} NMR (CDCl_3_, 100 MHz): *δ* 145.3, 143.7, 136.9, 136.5, 134.8, 133.8, 130.5, 129.0, 128.6, 128.4, 127.9, 127.8, 125.2, 123.8, 117.7, 114.7, 112.6; anal. calcd for C_19_H_12_Cl_2_N_2_: C, 67.27; H, 3.57; N, 8.26; found C, 67.54; H, 3.58; N, 8.09%.

#### 3-(2-Bromophenyl)-2-phenylimidazo[1,2-*a*]pyridine (3ae)

Semi solid (59 mg, 85%); ^1^H NMR (CDCl_3_, 400 MHz): *δ* 7.84 (d, *J* = 7.2 Hz, 1H), 7.72 (d, *J* = 8.8 Hz, 1H), 7.64–7.58 (m, 3H), 7.46–7.36 (m, 3H), 7.30–7.22 (m, 4H), 6.79–6.76 (m, 1H); ^13^C{^1^H} NMR (CDCl_3_, 100 MHz): *δ* 145.0, 143.0, 134.2, 134.1, 133.7, 131.4, 131.2, 128.56, 128.51, 128.0, 127.7, 126.5, 125.0, 124.0, 120.2, 117.6, 112.3; anal. calcd for C_19_H_13_BrN_2_: C, 65.35; H, 3.75; N, 8.02; found C, 65.11; H, 3.83; N, 8.14%.

#### General experimental procedure for the arylation of imidazo[2,1-*b*]thiazole (4a and 4b)

A mixture of imidazo[2,1-*b*]thiazole (0.20 mmol, 1 equiv.) and arylhydrazine hydrochloride (2, 0.26 mmol, 1.3 equiv.) was dissolved in 3 mL MeCN at room temperature in a reaction tube. Then 4 equiv. of DBU (120 μL) was added to the reaction mixture and stirred in air for 4 h. After completion of the reaction, the reaction mixture was extracted with ethyl acetate and washed with water (3 times). The organic phase was evaporated, and crude product was purified by column chromatography on silica gel (60–120 mesh) using petroleum ether and ethylacetate as an eluent to afford the pure product.

#### 5,6-Diphenylimidazo[2,1-*b*]thiazole (4a)

Yellow liquid (44 mg, 80%); ^1^H NMR (CDCl_3_, 400 MHz): *δ* 7.62–7.59 (m, 2H), 7.46–7.43 (m, 4H), 7.42–7.40 (m, 1H), 7.37 (d, *J* = 4.4 Hz, 1H), 7.29–7.27 (m, 2H), 7.24–7.20 (m, 1H), 6.81 (d, *J* = 4.8 Hz, 1H); ^13^C{^1^H} NMR (CDCl_3_, 100 MHz): *δ* 149.1, 134.6, 130.6, 129.38, 129.35, 128.49, 128.43, 127.7, 127.2, 117.6, 112.5; anal. calcd for C_17_H_12_N_2_S: C, 73.89; H, 4.38; N, 10.14; found C, 73.98; H, 4.31; N, 10.13%.

#### 6-Methoxy-2-phenyl-3-(*o*-tolyl)benzo[*d*]imidazo[2,1-*b*]thiazole (4b)

Semi solid (61 mg, 82%); ^1^H NMR (CDCl_3_, 400 MHz): *δ* 7.56–7.54 (m, 2H), 7.52–7.48 (m, 1H), 7.43 (dd, *J* = 7.6 Hz, 1.6 Hz, 2H), 7.36 (t, *J* = 8.0 Hz, 1H), 7.25–7.16 (m, 4H), 6.68 (dd, *J* = 8.8 Hz, 2.4 Hz, 1H), 6.47 (d, *J* = 9.2 Hz, 1H), 3.80 (s, 3H), 2.12 (s, 3H); ^13^C{^1^H} NMR (CDCl_3_, 100 MHz): *δ* 156.9, 146.5, 142.5, 139.4, 134.6, 131.9, 130.8, 130.1, 130.0, 128.4, 127.3, 127.2, 127.0, 126.9, 126.1, 123.2, 113.3, 113.1, 108.8, 55.9, 19.9; HRMS (ESI-TOF) *m*/*z*: [M + H]^+^ calcd for C_23_H_19_N_2_OS: 371.1213; found: 371.1203.

#### Experimental procedure for the synthesis of 2,3-diphenyl-6-(phenylethynyl)imidazo[1,2-*a*]pyridine (5)

A mixture of 6-bromo-2,3-diphenylimidazo[1,2-*a*]pyridine (3ra, 0.2 mmol, 70 mg), phenylacetylene (0.2 mmol, 22 μL), bis(triphenylphosphine)palladium(ii) dichloride (10 mol%, 14 mg) and copper iodide (10 mol%, 3.8 mg) was taken in a reaction vessel in 2 mL of a solution of DMF/Et_3_N (1/1, v/v) under argon and it was stirred at 50 °C temperature for 24 h. After cooling, the reaction mixture was extracted with ethyl acetate. The organic layer was evaporated, and crude product was purified by column chromatography on silica gel (60–120 mesh) using petroleum ether and ethylacetate as an eluent to afford the pure product. Pure product was obtained as a white solid (70 mg, 95%), mp: 189–191 °C; ^1^H NMR (CDCl_3_, 400 MHz): *δ* 8.13 (m, 1H), 7.68–7.63 (m, 3H), 7.57–7.44 (m, 7H), 7.36–7.24 (m, 7H); ^13^C{^1^H} NMR (CDCl_3_, 100 MHz): *δ* 143.7, 143.3, 133.9, 131.6, 130.9, 129.8, 129.4, 129.3, 128.7, 128.5, 128.4, 128.1, 127.8, 127.7, 126.2, 122.6, 121.4, 117.3, 109.2, 90.6, 85.8; anal. calcd for C_27_H_18_N_2_: C, 87.54; H, 4.90; N, 7.56; found C, 87.84; H, 4.72; N, 7.44%.

#### Experimental procedure for the synthesis of 2,3,6-triphenylimidazo[1,2-*a*]pyridine (6)

A mixture of 6-bromo-2,3-diphenylimidazo[1,2-*a*]pyridine (3ra, 0.2 mmol, 70 mg), phenyl boronic acid (0.2 mmol, 24 mg), tetrakis(triphenylphosphine) palladium(0) (2 mol%, 4.6 mg) and potassium carbonate (2.1 equiv. 58 mg) was taken in a reaction vessel in 2 mL of a solution of DMF/H_2_O (2/1, v/v) under argon and it was stirred at 100 °C temperature for 4 h. After completion of the reaction, the reaction mixture was cooled to room temperature, quenched with water, and extracted with dichloromethane. The organic layer was evaporated, and crude product was purified by column chromatography on silica gel (60–120 mesh) using petroleum ether and ethylacetate as an eluent to afford the pure product. Pure product was obtained as a white solid (52 mg, 75%), mp: 165–167 °C; ^1^H NMR (CDCl_3_, 400 MHz): *δ* 8.11 (s, 1H), 7.75 (d, *J* = 9.6 Hz, 1H), 7.69–7.67 (m, 2H), 7.56–7.46 (m, 8H), 7.44–7.41 (m, 2H), 7.38–7.34 (m, 1H), 7.31–7.25 (m, 3H); ^13^C{^1^H} NMR (CDCl_3_, 100 MHz): *δ* 144.2, 143.1, 137.7, 134.2, 130.8, 129.9, 129.7, 129.18, 129.12, 128.4, 128.2, 127.9, 127.6, 127.09, 127.00, 125.6, 121.6, 120.6, 117.5; anal. calcd for C_25_H_18_N_2_: C, 86.68; H, 5.24; N, 8.09; found C, 86.90; H, 5.13; N, 7.97%.

## Conflicts of interest

There are no conflicts to declare.

## Supplementary Material

RA-008-C8RA01474D-s001

RA-008-C8RA01474D-s002
