# Supplementary material for: Metal-free C–H arylation of imidazoheterocycles with aryl hydrazines
Source: RSC Adv. 2018 Apr 3;8(22):12360–7. doi: 10.1039/c8ra01474d (PMC9079221; doi:10.1039/c8ra01474d)
Supplement: RA-008-C8RA01474D-s001 [file RA-008-C8RA01474D-s001.pdf]

Electronic Supplementary Material (ESI) for RSC Advances.
This journal is © The Royal Society of Chemistry 2018

Supporting Information

Metal-free C-H arylation of imidazoheterocycles with aryl hydrazines

Sourav Jana,† Sadhanendu Samanta,† Avik K. Bagdi,‡ Valerii Z. Shirinian,§ and Alakananda Hajra*†

†*Department of Chemistry, Visva-Bharati (A Central University), Santiniketan 731235, India*

‡*Department of Chemistry, TDB College, Raniganj, Burdwan 713347, India*

§*Zelinsky Institute of Organic Chemistry RAS, 47 Leninsky prosp., 119991, Moscow, Russian Federation.*

Contents

Sl. No.	Topics	Page No.
1.	Structure Determination (X-ray crystallographic data **3oa**)	S2
2.	NMR spectra [^{1}H and ^{13}C{^{1}H}] of synthesized products	S3-S59

1. Structure Determination (X-ray crystallographic data for 3oa):

The Colourless crystals of **3oa** was obtained by crystallization from a solution in ethanol/hexane after purification by column chromatography. Chemical formula $C_{20}H_{16}N_2$.

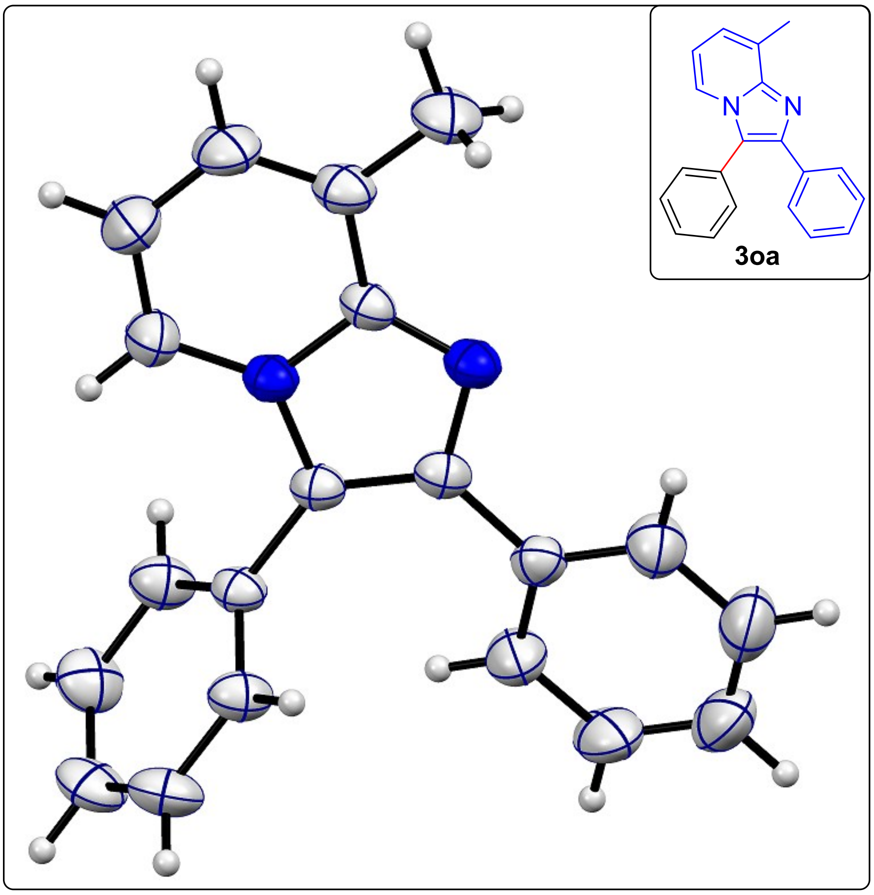

ORTEP (with 50% probability) diagram for the structure 8-methyl-2,3-diphenylimidazo[1,2-*a*]pyridine **(3oa)**.

Wavelength	0.71073 Å	
Formula	$C_{20}H_{16}N_2$	
Crystal system	triclinic	
Space group	P b c a	
Unit cell dimensions	a = 9.7875 (12) Å	α = 73.134(6) °
	b = 10.5334 (12) Å	β = 82.652(6) °
	c = 15.8394 (18) Å	γ = 75.019(6)°
Volume	1507.1(3) $Å^3$	
Z	4	
R-factor (%)	7.13	

The crystallographic data have been deposited with the Cambridge Crystallographic Data Centreas supplementary publication with a CCDC reference number CCDC **1589816**.

2. NMR spectra [^{1}H and $^{13}C\{1H\}$] of synthesized products:

1H of VBSJ-371 2nd

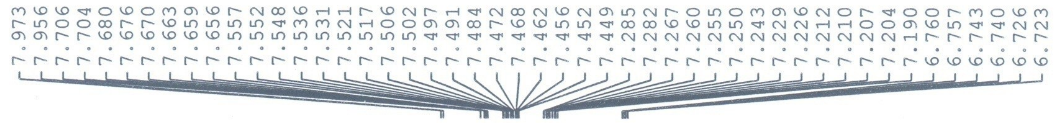

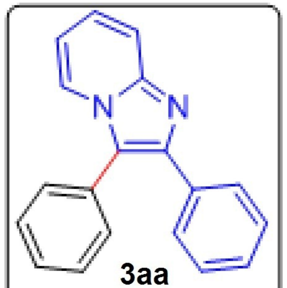

3aa

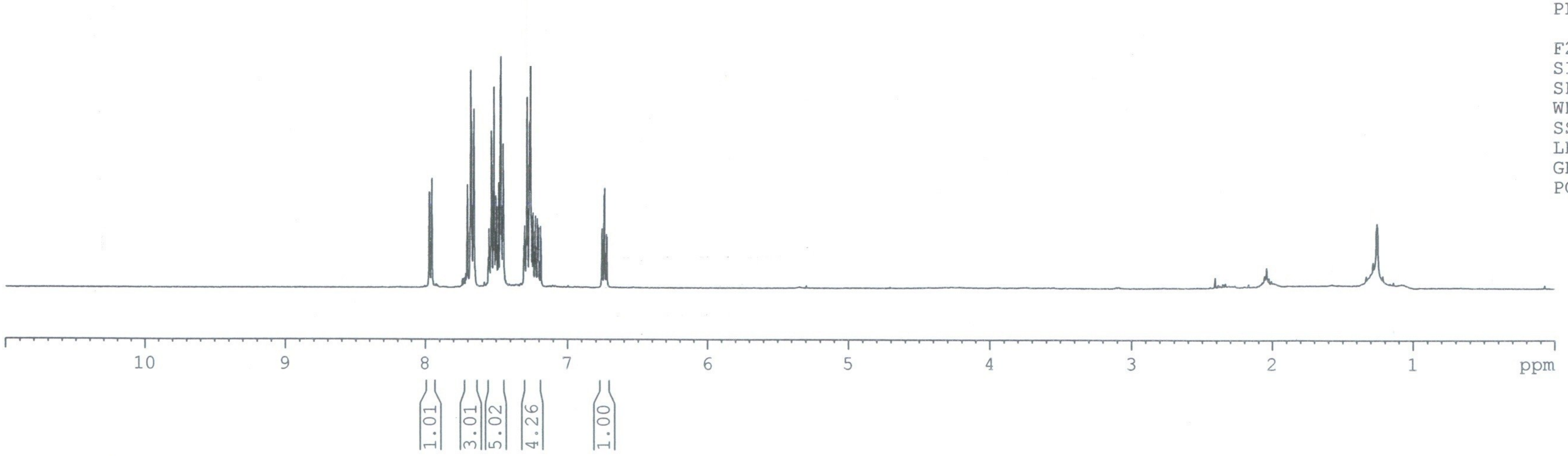

BRUKER

Current Data Parameters
NAME Dr.A.HAJRA 2018
EXPNO 299
PROCNO 1

F2 - Acquisition Parameters
Date_ 20180214
Time 20.21
INSTRUM spect
PROBHD 5 mm PABBO BB/
PULPROG zg30
TD 32768
SOLVENT CDCl3
NS 16
DS 1
SWH 8223.685 Hz
FIDRES 0.250967 Hz
AQ 1.9922944 sec
RG 186.42
DW 60.800 usec
DE 6.50 usec
TE 296.0 K
D1 1.00000000 sec
TD0 1

======== CHANNEL f1 ========
SFO1 400.1524711 MHz
NUC1 1H
P1 14.75 usec
PLW1 12.00000000 W

F2 - Processing parameters
SI 16384
SF 400.1500369 MHz
WDW EM
SSB 0
LB 0.30 Hz
GB 0
PC 1.00

13C of VBSJ-371

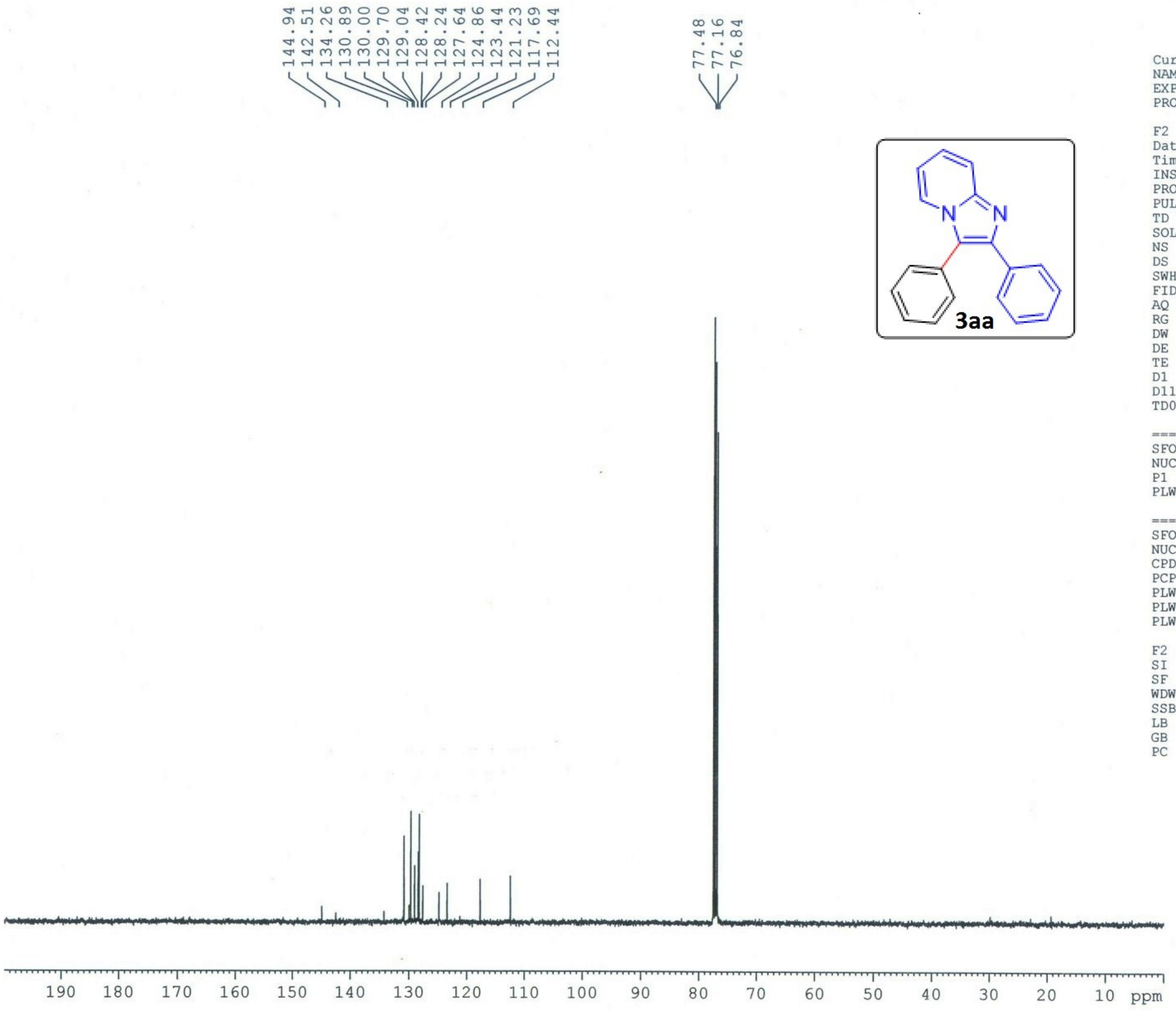

BRUKER

Current Data Parameters
NAME Dr.A.HAJRA 2017
EXPNO 2058
PROCNO 1

F2 - Acquisition Parameters
Date_ 20171205
Time 12.32
INSTRUM spect
PROBHD 5 mm PABBO BB/
PULPROG zgpg30
TD 32768
SOLVENT CDCl3
NS 1024
DS 2
SWH 24038.461 Hz
FIDRES 0.733596 Hz
AQ 0.6815744 sec
RG 168.31
DW 20.800 usec
DE 6.50 usec
TE 294.6 K
D1 2.00000000 sec
D11 0.03000000 sec
TD0 1

======== CHANNEL f1 ========
SFO1 100.6278588 MHz
NUC1 13C
P1 8.90 usec
PLW1 54.00000000 W

======== CHANNEL f2 ========
SFO2 400.1516006 MHz
NUC2 1H
CPDPRG[2 waltz16
PCPD2 90.00 usec
PLW2 12.00000000 W
PLW12 0.32231000 W
PLW13 0.16212000 W

F2 - Processing parameters
SI 16384
SF 100.6177839 MHz
WDW EM
SSB 0
LB 1.00 Hz
GB 0
PC 1.00

1H of VBSJ-380

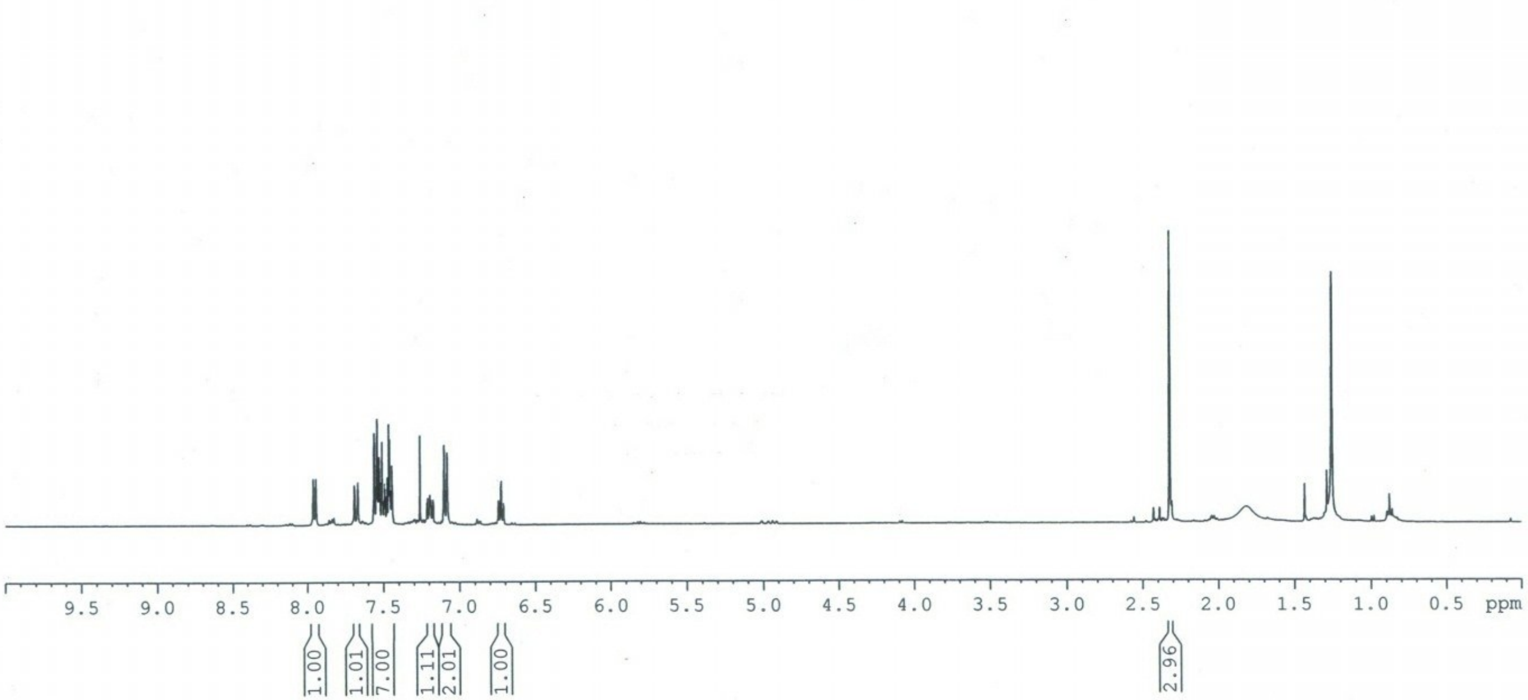

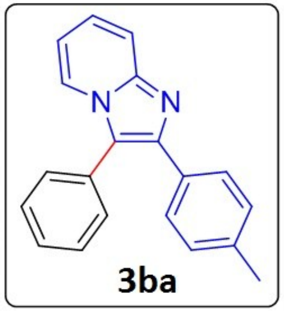

BRUKER

Current Data Parameters
NAME Dr.A.HAJRA 2017
EXPNO 1602
PROCNO 1

F2 - Acquisition Parameters
Date_ 20170920
Time 17.44
INSTRUM spect
PROBHD 5 mm PABBO BB/
PULPROG zg30
TD 32768
SOLVENT CDCl3
NS 32
DS 1
SWH 8223.685 Hz
FIDRES 0.250967 Hz
AQ 1.9922944 sec
RG 120.16
DW 60.800 usec
DE 6.50 usec
TE 297.5 K
D1 1.00000000 sec
TD0 1

======== CHANNEL f1 ========
SFO1 400.1524711 MHz
NUC1 1H
P1 14.75 usec
PLW1 12.00000000 W

F2 - Processing parameters
SI 16384
SF 400.1500095 MHz
WDW EM
SSB 0
LB 0.30 Hz
GB 0
PC 1.00

13C of VBSJ-380

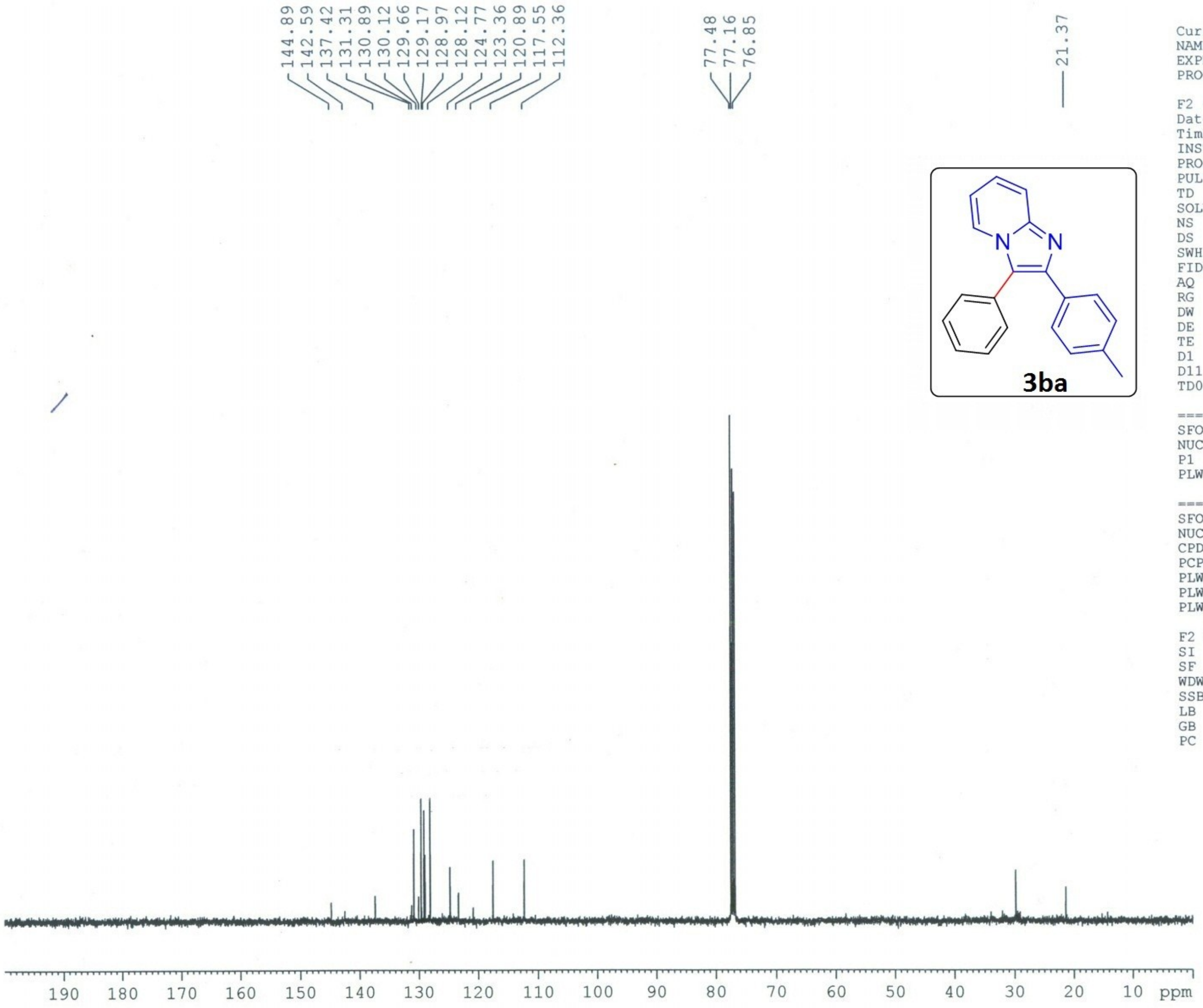

BRUKER

Current Data Parameters
NAME Dr.A.HAJRA 2017
EXPNO 1603
PROCNO 1

F2 - Acquisition Parameters
Date_ 20170920
Time 18.09
INSTRUM spect
PROBHD 5 mm PABBO BB/
PULPROG zgpg30
TD 32768
SOLVENT CDCl3
NS 380
DS 2
SWH 24038.461 Hz
FIDRES 0.733596 Hz
AQ 0.6815744 sec
RG 120.16
DW 20.800 usec
DE 6.50 usec
TE 298.2 K
D1 2.00000000 sec
D11 0.03000000 sec
TD0 1

======== CHANNEL f1 ========
SFO1 100.6278588 MHz
NUC1 13C
P1 8.90 usec
PLW1 54.00000000 W

======== CHANNEL f2 ========
SFO2 400.1516006 MHz
NUC2 1H
CPDPRG[2 waltz16
PCPD2 90.00 usec
PLW2 12.00000000 W
PLW12 0.32231000 W
PLW13 0.16212000 W

F2 - Processing parameters
SI 16384
SF 100.6177839 MHz
WDW EM
SSB 0
LB 1.00 Hz
GB 0
PC 1.20

1H of VBSJ-361

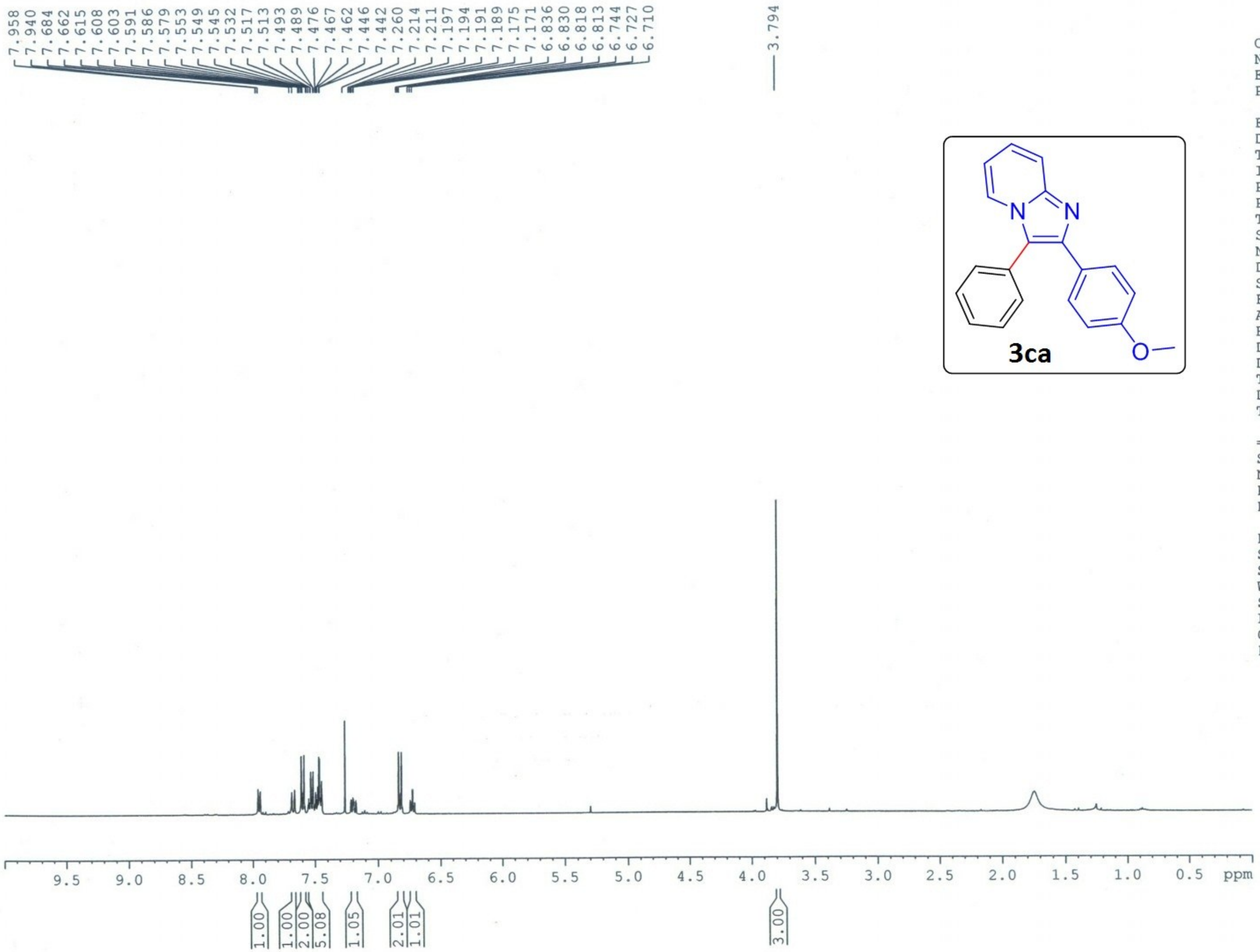

3ca

BRUKER

Current Data Parameters
NAME Dr.A.HAJRA 2017
EXPNO 1472
PROCNO 1

F2 - Acquisition Parameters
Date_ 20170825
Time 22.11
INSTRUM spect
PROBHD 5 mm PABBO BB/
PULPROG zg30
TD 32768
SOLVENT CDCl3
NS 12
DS 1
SWH 8223.685 Hz
FIDRES 0.250967 Hz
AQ 1.9922944 sec
RG 186.42
DW 60.800 usec
DE 6.50 usec
TE 298.1 K
D1 1.00000000 sec
TD0 1

======== CHANNEL f1 ========
SFO1 400.1524711 MHz
NUC1 1H
P1 14.75 usec
PLW1 12.00000000 W

F2 - Processing parameters
SI 16384
SF 400.1500095 MHz
WDW EM
SSB 0
LB 0.30 Hz
GB 0
PC 1.00

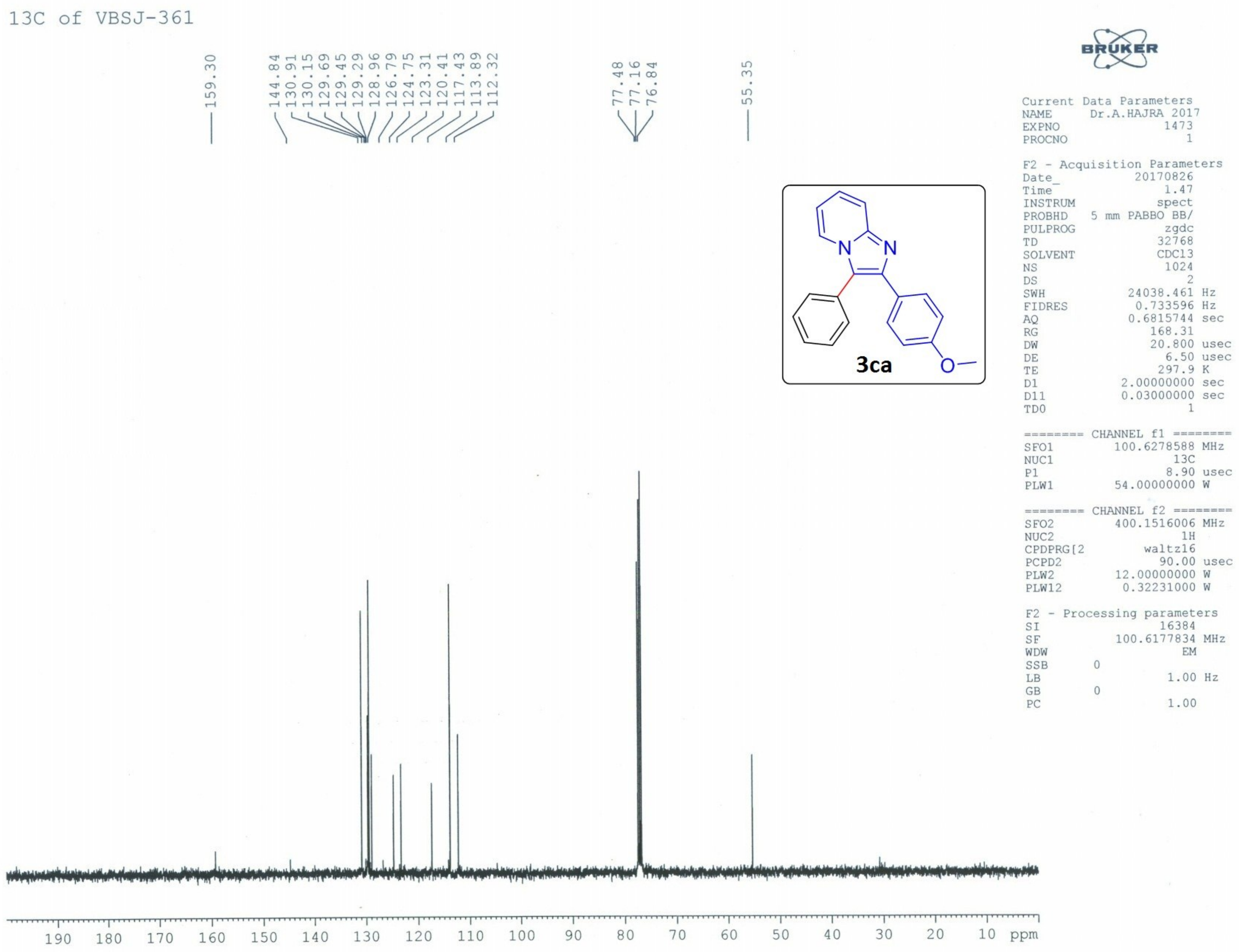
13C of VBSJ-361
159.30
144.84
130.91
130.15
129.69
129.45
129.29
128.96
126.79
124.75
123.31
120.41
117.43
113.89
112.32
77.48
77.16
76.84
55.35
3ca
N
N
O
BRUKER
Current Data Parameters
NAME Dr.A.HAJRA 2017
EXPNO 1473
PROCNO 1
F2 - Acquisition Parameters
Date_ 20170826
Time 1.47
INSTRUM spect
PROBHD 5 mm PABBO BB/
PULPROG zgdc
TD 32768
SOLVENT CDCl3
NS 1024
DS 2
SWH 24038.461 Hz
FIDRES 0.733596 Hz
AQ 0.6815744 sec
RG 168.31
DW 20.800 usec
DE 6.50 usec
TE 297.9 K
D1 2.00000000 sec
D11 0.03000000 sec
TD0 1
======== CHANNEL f1 ========
SFO1 100.6278588 MHz
NUC1 13C
P1 8.90 usec
PLW1 54.00000000 W
======== CHANNEL f2 ========
SFO2 400.1516006 MHz
NUC2 1H
CPDPRG[2 waltz16
PCPD2 90.00 usec
PLW2 12.00000000 W
PLW12 0.32231000 W
F2 - Processing parameters
SI 16384
SF 100.6177834 MHz
WDW EM
SSB 0
LB 1.00 Hz
GB 0
PC 1.00
190 180 170 160 150 140 130 120 110 100 90 80 70 60 50 40 30 20 10 ppm

1H of VBSJ-367

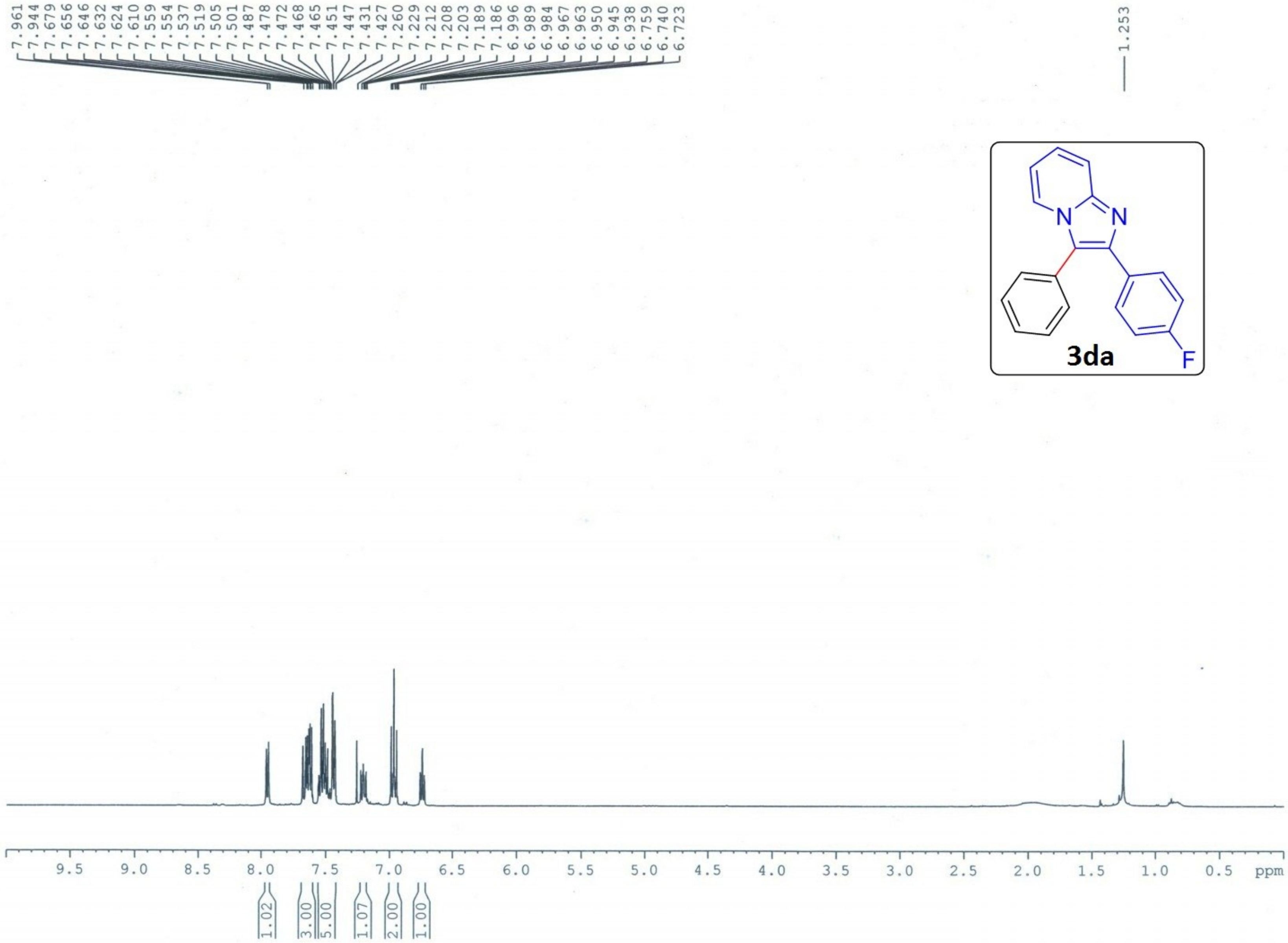

BRUKER

Current Data Parameters

NAME	Dr.A.HAJRA 2017
EXPNO	1554
PROCNO	1

F2 - Acquisition Parameters

Date_	20170912
Time	17.34
INSTRUM	spect
PROBHD	5 mm PABBO BB/
PULPROG	zg30
TD	32768
SOLVENT	CDCl3
NS	24
DS	1
SWH	8223.685 Hz
FIDRES	0.250967 Hz
AQ	1.9922944 sec
RG	135.7
DW	60.800 usec
DE	6.50 usec
TE	297.7 K
D1	1.00000000 sec
TD0	1

======== CHANNEL f1 ========

SFO1	400.1524711 MHz
NUC1	1H
P1	14.75 usec
PLW1	12.00000000 W

F2 - Processing parameters

SI	16384
SF	400.1500095 MHz
WDW	EM
SSB	0
LB	0.30 Hz
GB	0
PC	1.00

13C of VBSJ-367

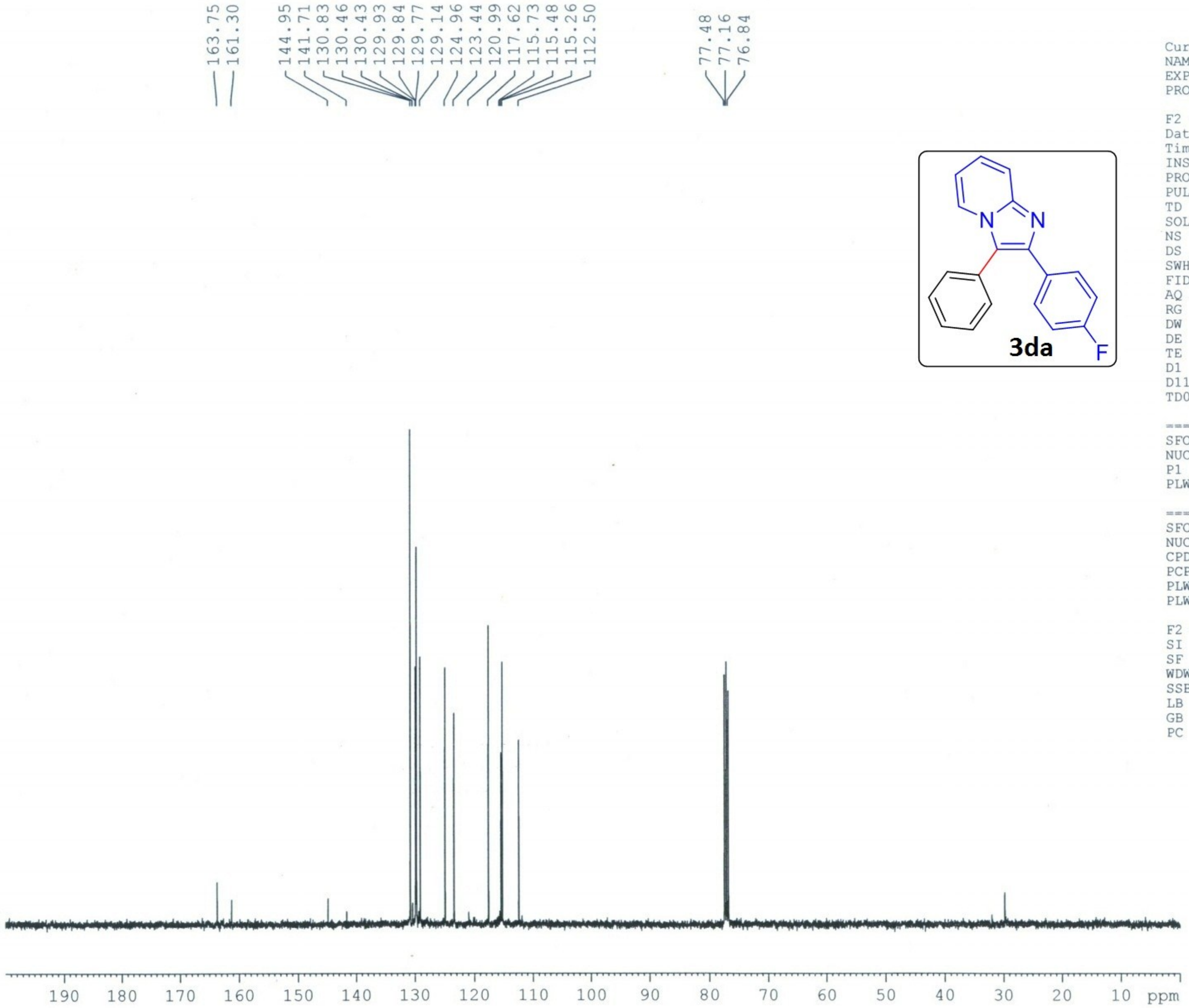


```
Current Data Parameters
NAME       Dr.A.HAJRA 2017
EXPNO                 1555
PROCNO                   1

F2 - Acquisition Parameters
Date_             20170912
Time                 18.14
INSTRUM              spect
PROBHD    5 mm PABBO BB/
PULPROG               zgdc
TD                   32768
SOLVENT              CDC13
NS                     800
DS                       2
SWH              24038.461 Hz
FIDRES            0.733596 Hz
AQ               0.6815744 sec
RG                   135.7
DW                  20.800 usec
DE                    6.50 usec
TE                   299.1 K
D1              2.00000000 sec
D11             0.03000000 sec
TD0                      1

======== CHANNEL f1 ========
SFO1          100.6278588 MHz
NUC1                  13C
P1                   8.90 usec
PLW1          54.00000000 W

======== CHANNEL f2 ========
SFO2          400.1516006 MHz
NUC2                   1H
CPDPRG[2          waltz16
PCPD2               90.00 usec
PLW2          12.00000000 W
PLW12          0.32231000 W

F2 - Processing parameters
SI                  16384
SF            100.6177837 MHz
WDW                    EM
SSB       0
LB                   1.00 Hz
GB        0
PC                   1.40
```

1H of VBSJ-378 2nd

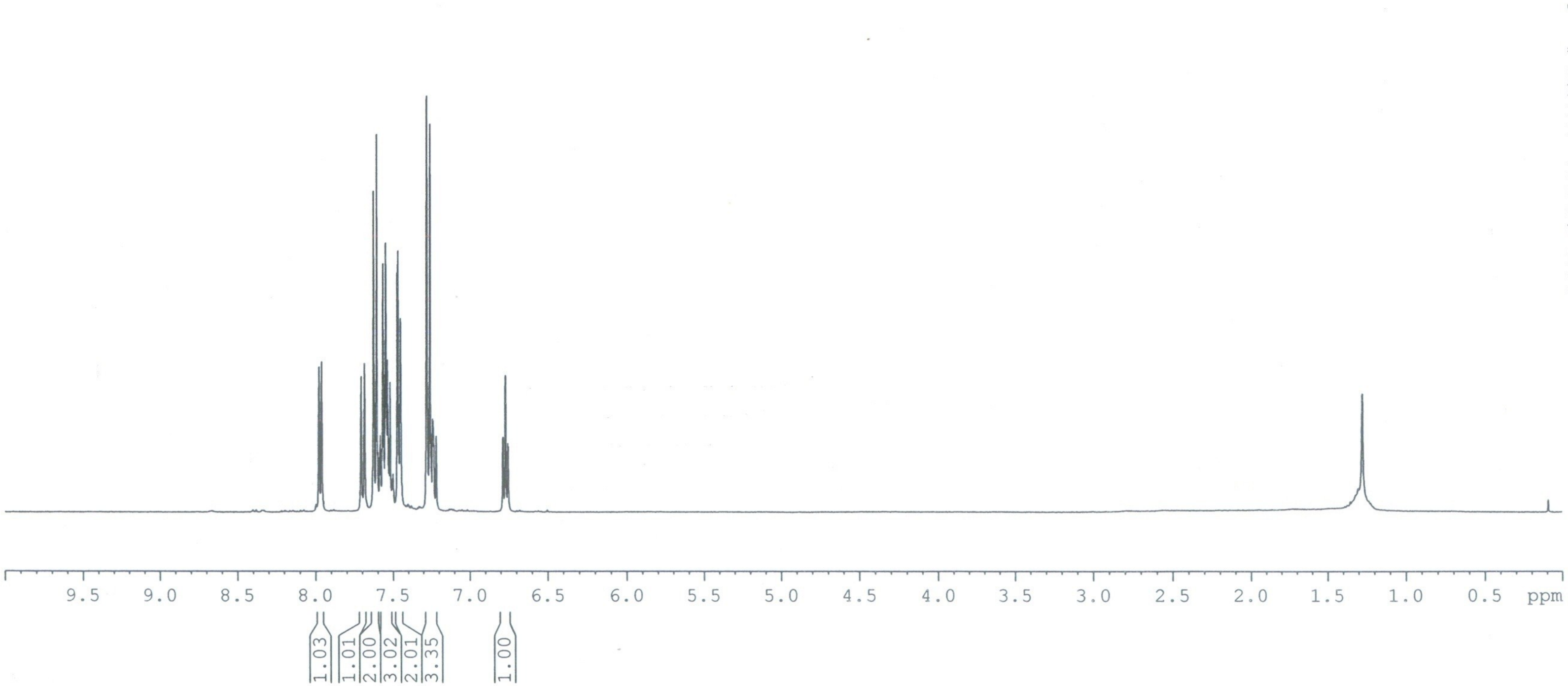

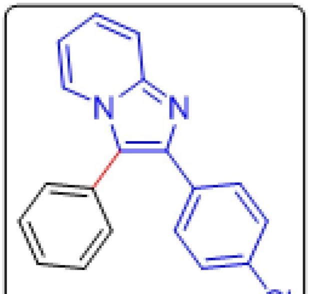

3ea

BRUKER

Current Data Parameters
NAME Dr.A.HAJRA 2018
EXPNO 296
PROCNO 1

F2 - Acquisition Parameters
Date_ 20180214
Time 19.35
INSTRUM spect
PROBHD 5 mm PABBO BB/
PULPROG zg30
TD 32768
SOLVENT CDCl3
NS 16
DS 1
SWH 8223.685 Hz
FIDRES 0.250967 Hz
AQ 1.9922944 sec
RG 120.16
DW 60.800 usec
DE 6.50 usec
TE 296.0 K
D1 1.00000000 sec
TD0 1

======== CHANNEL f1 ========
SFO1 400.1524711 MHz
NUC1 1H
P1 14.75 usec
PLW1 12.00000000 W

F2 - Processing parameters
SI 16384
SF 400.1500000 MHz
WDW EM
SSB 0
LB 0.30 Hz
GB 0
PC 1.00

13C of VBSJ-378

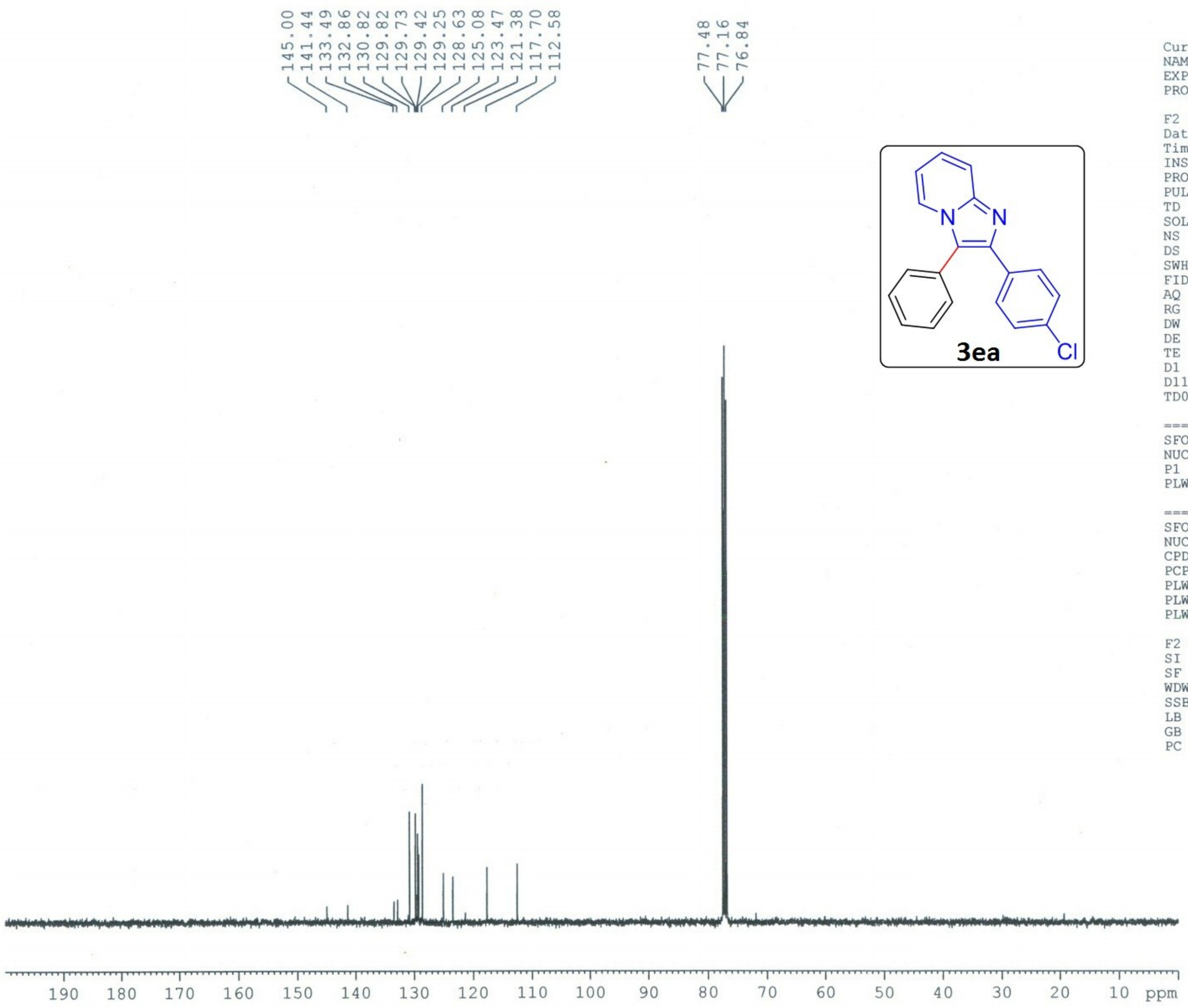

3ea

```
Current Data Parameters
NAME      Dr.A.HAJRA 2017
EXPNO               1734
PROCNO                 1

F2 - Acquisition Parameters
Date_           20171026
Time               20.24
INSTRUM            spect
PROBHD   5 mm PABBO BB/
PULPROG          zgpg30
TD                 32768
SOLVENT            CDCl3
NS                   512
DS                     2
SWH            24038.461 Hz
FIDRES          0.733596 Hz
AQ             0.6815744 sec
RG                168.31
DW                20.800 usec
DE                  6.50 usec
TE                 297.8 K
D1            2.00000000 sec
D11           0.03000000 sec
TD0                    1

======== CHANNEL f1 ========
SFO1         100.6278588 MHz
NUC1                 13C
P1                  8.90 usec
PLW1         54.00000000 W

======== CHANNEL f2 ========
SFO2         400.1516006 MHz
NUC2                  1H
CPDPRG[2         waltz16
PCPD2              90.00 usec
PLW2         12.00000000 W
PLW12         0.32231000 W
PLW13         0.16212000 W

F2 - Processing parameters
SI                 16384
SF           100.6177836 MHz
WDW                   EM
SSB      0
LB                  1.00 Hz
GB       0
PC                  1.20
```

1H of VBSJ-386

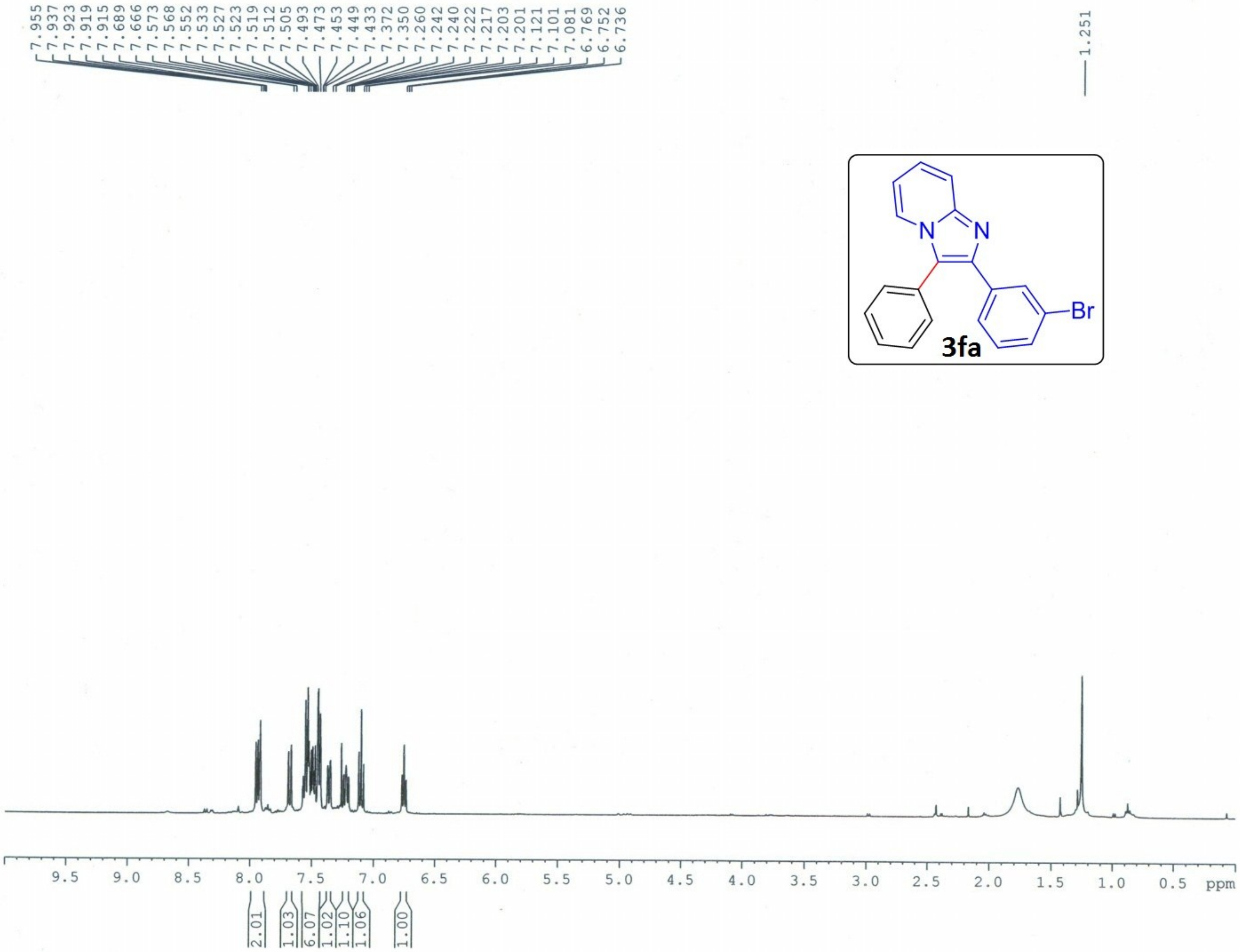

Current Data Parameters
NAME Dr.A.HAJRA 2017
EXPNO 1620
PROCNO 1

F2 - Acquisition Parameters
Date_ 20170924
Time 10.19
INSTRUM spect
PROBHD 5 mm PABBO BB/
PULPROG zg30
TD 32768
SOLVENT CDCl3
NS 24
DS 1
SWH 8223.685 Hz
FIDRES 0.250967 Hz
AQ 1.9922944 sec
RG 106.66
DW 60.800 usec
DE 6.50 usec
TE 296.6 K
D1 1.00000000 sec
TD0 1

======== CHANNEL f1 ========
SFO1 400.1524711 MHz
NUC1 1H
P1 14.75 usec
PLW1 12.00000000 W

F2 - Processing parameters
SI 16384
SF 400.1500096 MHz
WDW EM
SSB 0
LB 0.30 Hz
GB 0
PC 1.00

13C of VBSJ-386

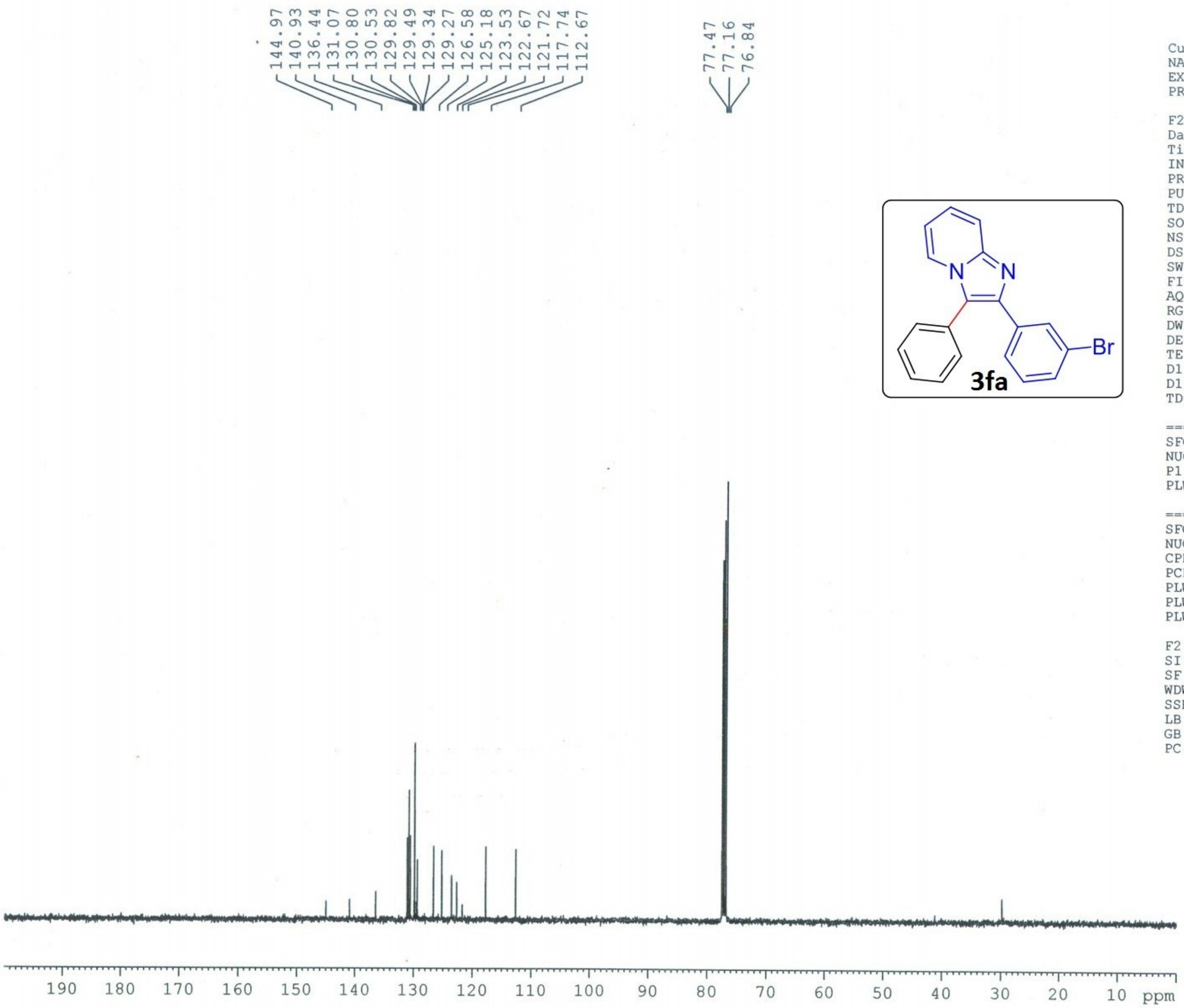

3fa

Current Data Parameters
NAME Dr.A.HAJRA 2017
EXPNO 1621
PROCNO 1

F2 - Acquisition Parameters
Date_ 20170924
Time 10.45
INSTRUM spect
PROBHD 5 mm PABBO BB/
PULPROG zgpg30
TD 32768
SOLVENT CDCl3
NS 512
DS 2
SWH 24038.461 Hz
FIDRES 0.733596 Hz
AQ 0.6815744 sec
RG 106.66
DW 20.800 usec
DE 6.50 usec
TE 297.6 K
D1 2.00000000 sec
D11 0.03000000 sec
TD0 1

======== CHANNEL f1 ========
SFO1 100.6278588 MHz
NUC1 13C
P1 8.90 usec
PLW1 54.00000000 W

======== CHANNEL f2 ========
SFO2 400.1516006 MHz
NUC2 1H
CPDPRG[2 waltz16
PCPD2 90.00 usec
PLW2 12.00000000 W
PLW12 0.32231000 W
PLW13 0.16212000 W

F2 - Processing parameters
SI 16384
SF 100.6177848 MHz
WDW EM
SSB 0
LB 1.00 Hz
GB 0
PC 0.50

1H of VBSJ-382

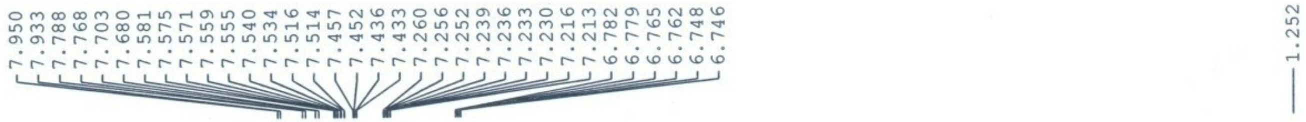

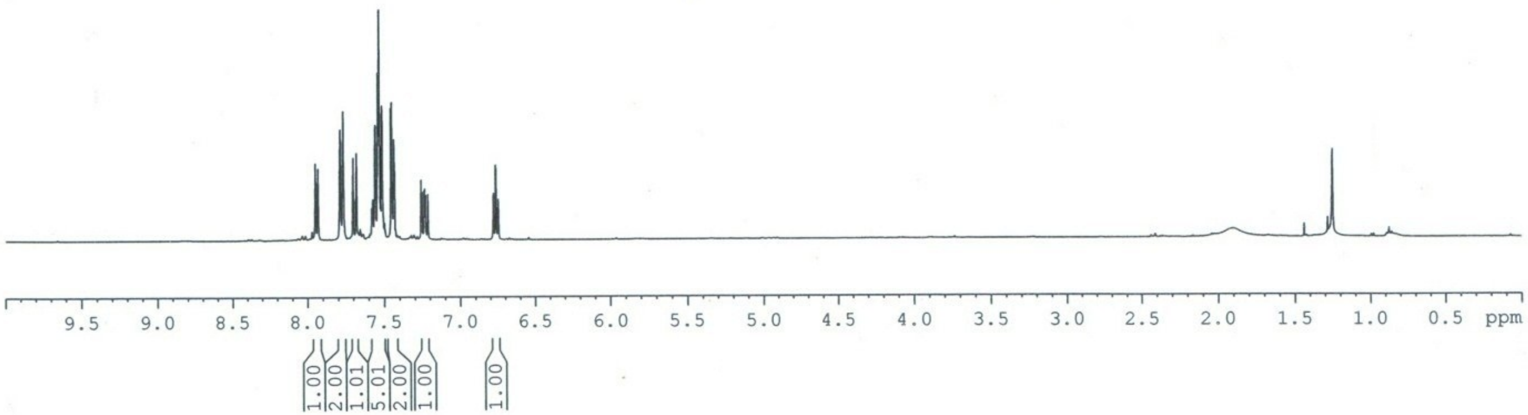

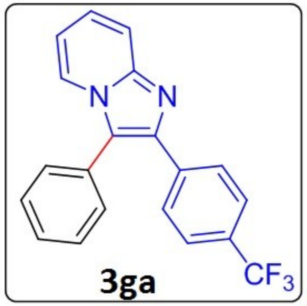

Current Data Parameters
NAME Dr.A.HAJRA 2017
EXPNO 1614
PROCNO 1

F2 - Acquisition Parameters
Date_ 20170923
Time 9.44
INSTRUM spect
PROBHD 5 mm PABBO BB/
PULPROG zg30
TD 32768
SOLVENT CDCl3
NS 32
DS 1
SWH 8223.685 Hz
FIDRES 0.250967 Hz
AQ 1.9922944 sec
RG 106.66
DW 60.800 usec
DE 6.50 usec
TE 297.3 K
D1 1.00000000 sec
TD0 1

======== CHANNEL f1 ========
SFO1 400.1524711 MHz
NUC1 1H
P1 14.75 usec
PLW1 12.00000000 W

F2 - Processing parameters
SI 16384
SF 400.1500095 MHz
WDW EM
SSB 0
LB 0.30 Hz
GB 0
PC 1.00

13C of VBSJ-382

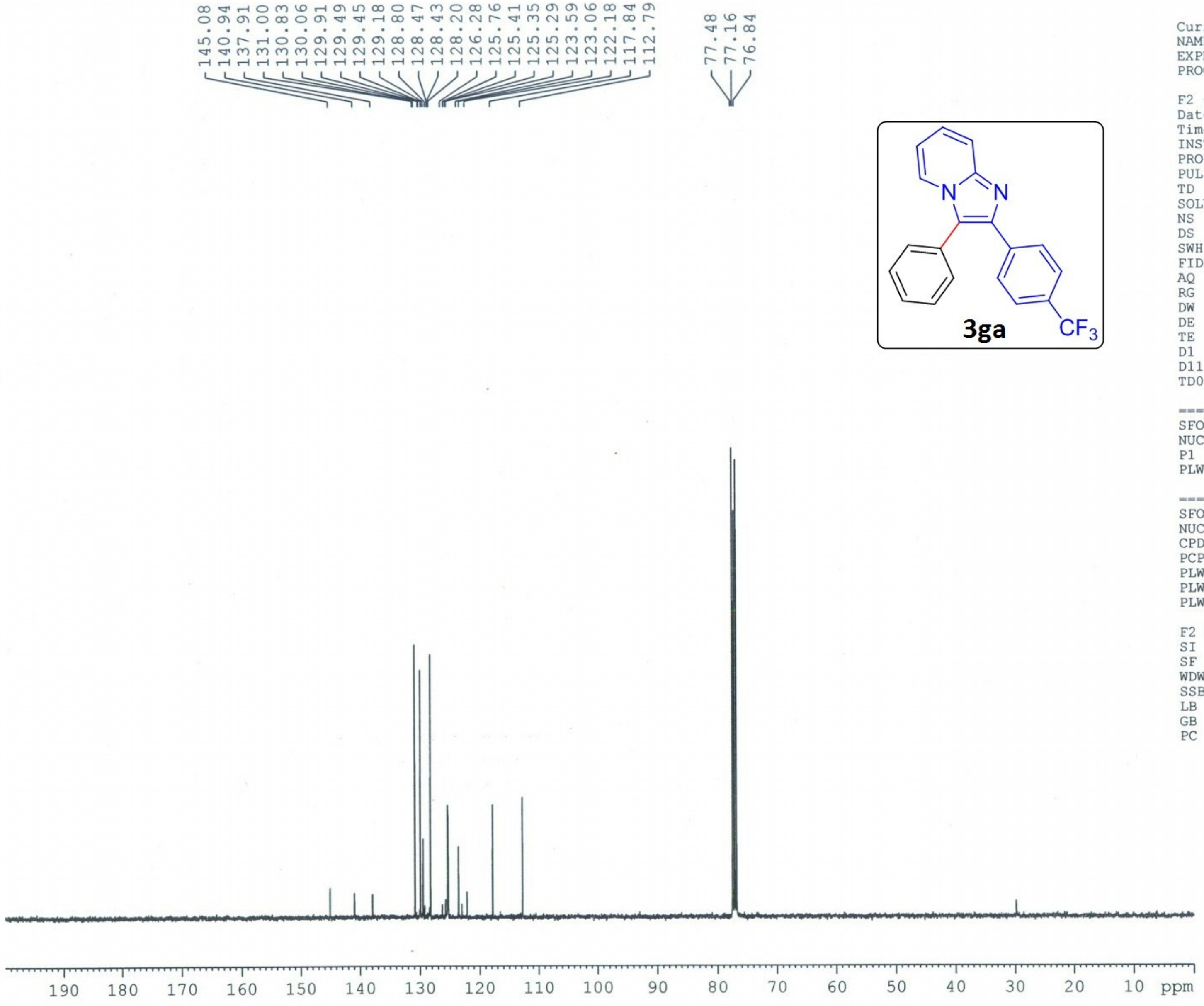

Current Data Parameters
NAME Dr.A.HAJRA 2017
EXPNO 1615
PROCNO 1

F2 - Acquisition Parameters
Date_ 20170923
Time 10.03
INSTRUM spect
PROBHD 5 mm PABBO BB/
PULPROG zgpg30
TD 32768
SOLVENT CDCl3
NS 1024
DS 2
SWH 24038.461 Hz
FIDRES 0.733596 Hz
AQ 0.6815744 sec
RG 106.66
DW 20.800 usec
DE 6.50 usec
TE 298.0 K
D1 2.00000000 sec
D11 0.03000000 sec
TD0 1

======== CHANNEL f1 ========
SFO1 100.6278588 MHz
NUC1 13C
P1 8.90 usec
PLW1 54.00000000 W

======== CHANNEL f2 ========
SFO2 400.1516006 MHz
NUC2 1H
CPDPRG[2 waltz16
PCPD2 90.00 usec
PLW2 12.00000000 W
PLW12 0.32231000 W
PLW13 0.16212000 W

F2 - Processing parameters
SI 16384
SF 100.6177842 MHz
WDW EM
SSB 0
LB 1.00 Hz
GB 0
PC 0.50

1H of VBSJ-393

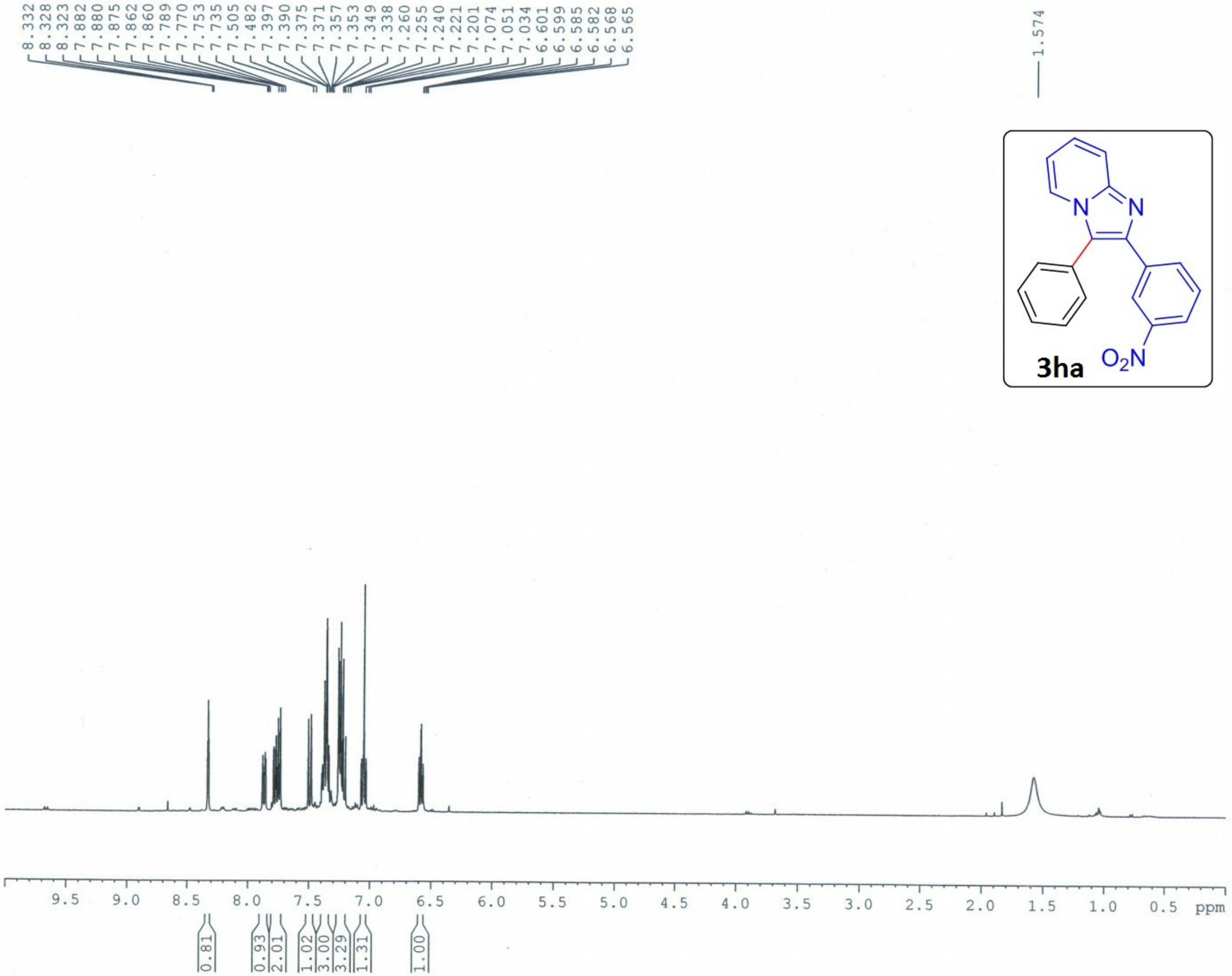

3ha

Current Data Parameters
NAME Dr.A.HAJRA 2017
EXPNO 1666
PROCNO 1

F2 - Acquisition Parameters
Date_ 20171019
Time 8.54
INSTRUM spect
PROBHD 5 mm PABBO BB/
PULPROG zg30
TD 32768
SOLVENT CDCl3
NS 32
DS 1
SWH 8223.685 Hz
FIDRES 0.250967 Hz
AQ 1.9922944 sec
RG 186.42
DW 60.800 usec
DE 6.50 usec
TE 295.2 K
D1 1.00000000 sec
TD0 1

======== CHANNEL f1 ========
SFO1 400.1524711 MHz
NUC1 1H
P1 14.75 usec
PLW1 12.00000000 W

F2 - Processing parameters
SI 16384
SF 400.1500932 MHz
WDW EM
SSB 0
LB 0.30 Hz
GB 0
PC 1.00

13C of VBSJ-393

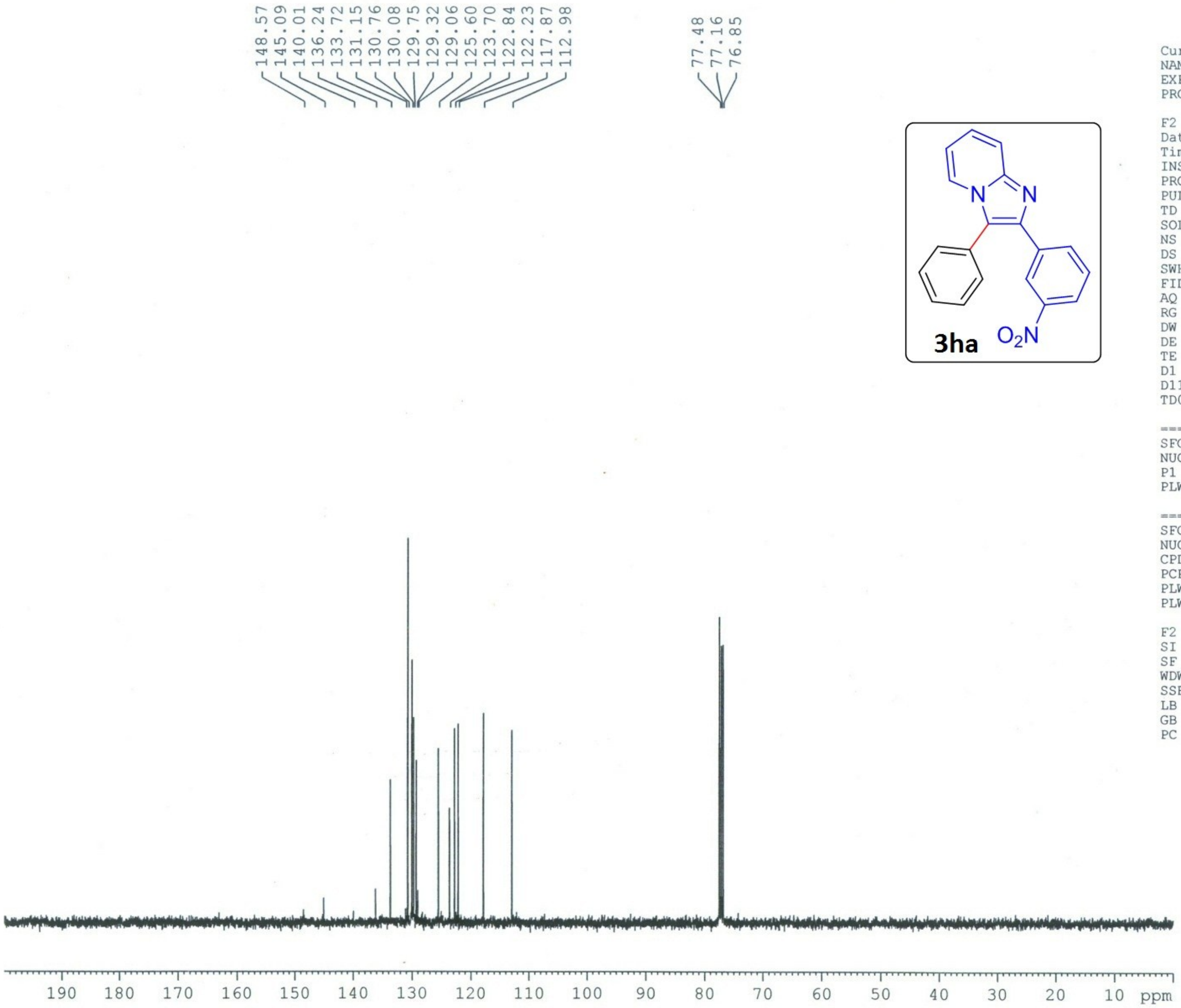

BRUKER

```
Current Data Parameters
NAME        Dr.A.HAJRA 2017
EXPNO                  1695
PROCNO                    1

F2 - Acquisition Parameters
Date_              20171019
Time                   9.20
INSTRUM               spect
PROBHD     5 mm PABBO BB/
PULPROG                zgdc
TD                    32768
SOLVENT               CDCl3
NS                      540
DS                        2
SWH               24038.461 Hz
FIDRES             0.733596 Hz
AQ                0.6815744 sec
RG                   148.91
DW                   20.800 usec
DE                     6.50 usec
TE                    296.7 K
D1               2.00000000 sec
D11              0.03000000 sec
TD0                       1

======== CHANNEL f1 ========
SFO1         100.6278588 MHz
NUC1                    13C
P1                     8.90 usec
PLW1          54.00000000 W

======== CHANNEL f2 ========
SFO2         400.1516006 MHz
NUC2                     1H
CPDPRG[2            waltz16
PCPD2                 90.00 usec
PLW2          12.00000000 W
PLW12          0.32231000 W

F2 - Processing parameters
SI                    16384
SF           100.6177840 MHz
WDW                      EM
SSB      0
LB                     1.00 Hz
GB       0
PC                     1.00
```

1H of of VBSJ-377

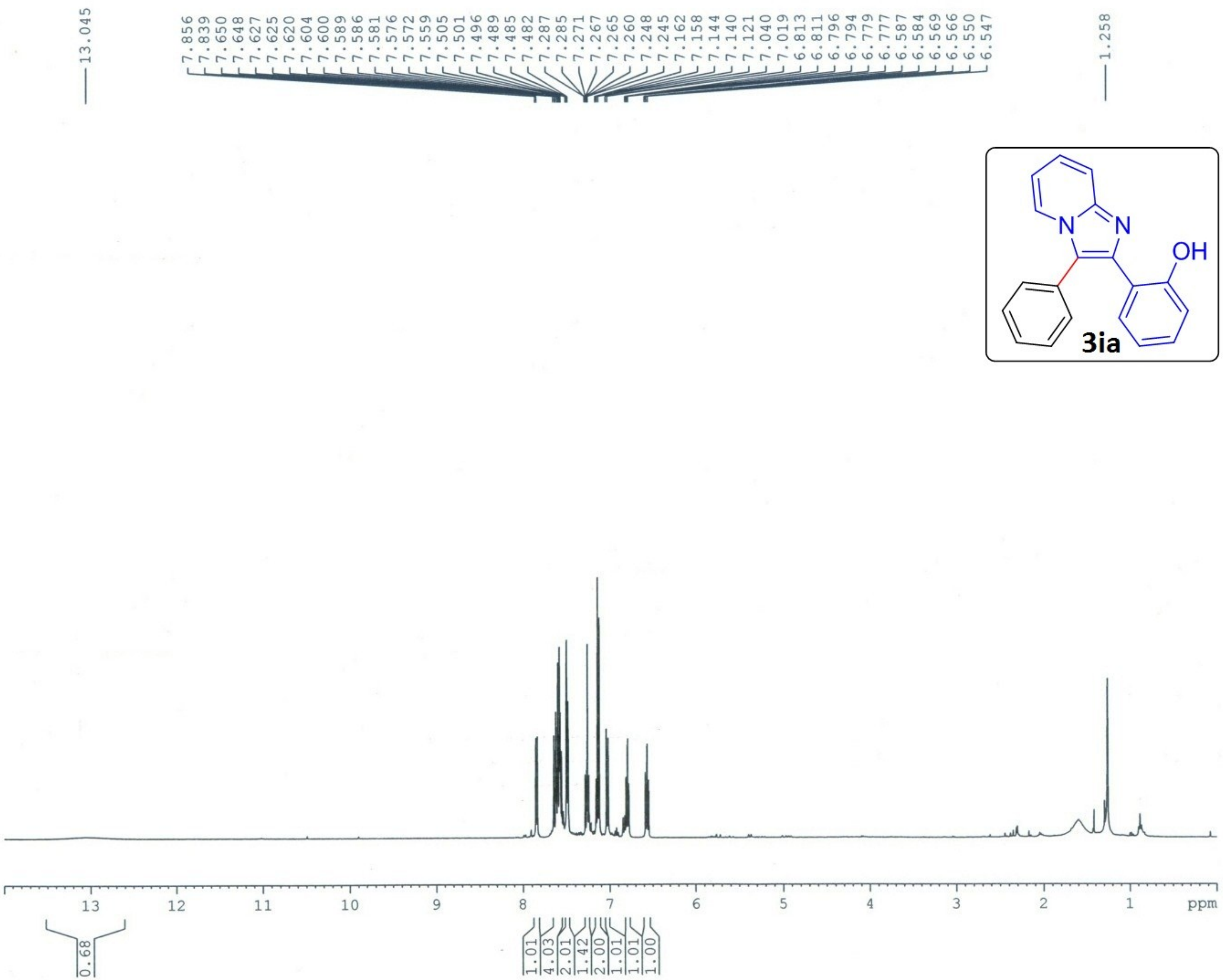

Current Data Parameters
NAME Dr.A.HAJRA 2017
EXPNO 1578
PROCNO 1

F2 - Acquisition Parameters
Date_ 20170917
Time 17.46
INSTRUM spect
PROBHD 5 mm PABBO BB/
PULPROG zg30
TD 32768
SOLVENT CDCl3
NS 32
DS 1
SWH 8223.685 Hz
FIDRES 0.250967 Hz
AQ 1.9922944 sec
RG 135.7
DW 60.800 usec
DE 6.50 usec
TE 298.1 K
D1 1.00000000 sec
TD0 1

======== CHANNEL f1 ========
SFO1 400.1524711 MHz
NUC1 1H
P1 14.75 usec
PLW1 12.00000000 W

F2 - Processing parameters
SI 16384
SF 400.1500095 MHz
WDW EM
SSB 0
LB 0.30 Hz
GB 0
PC 1.00

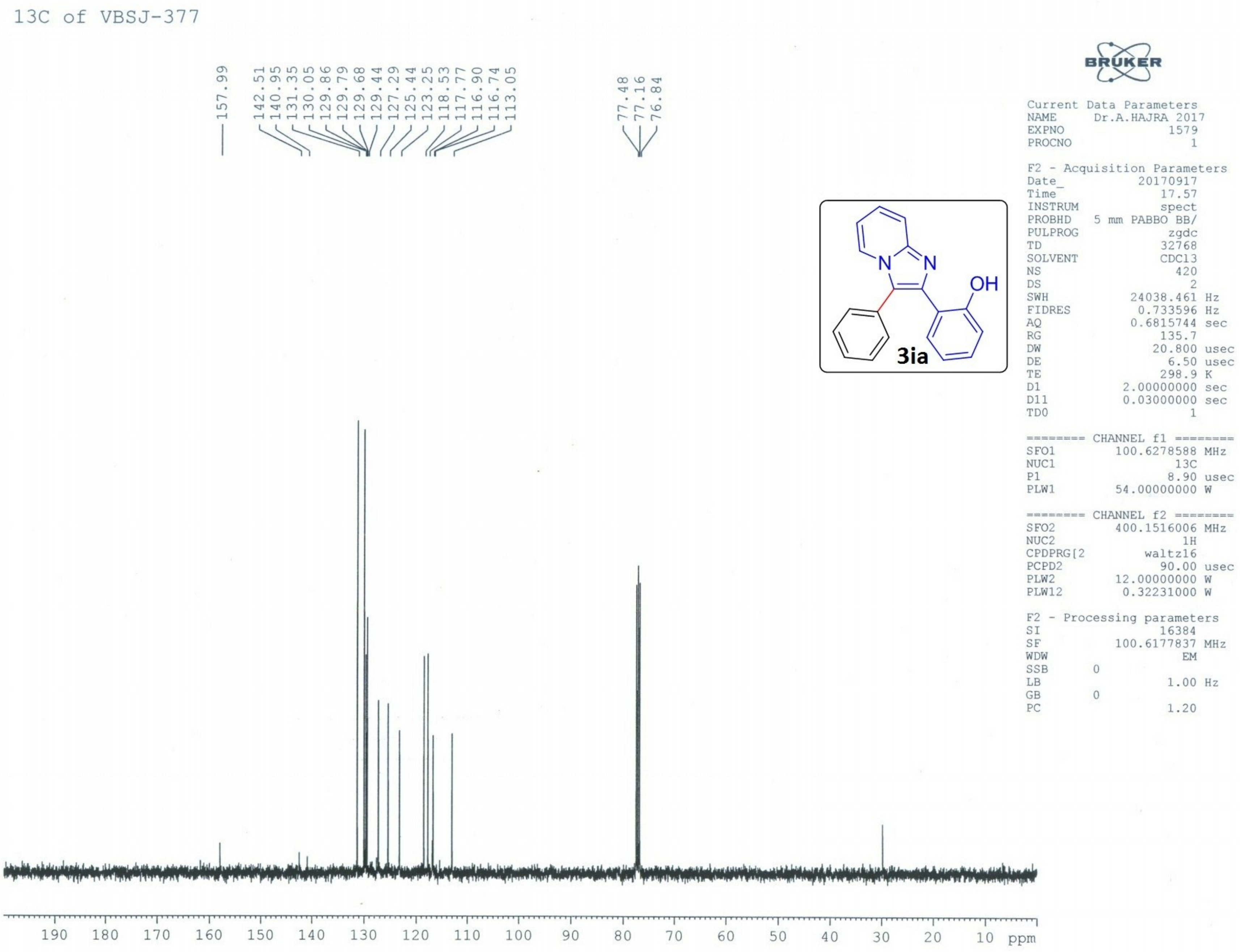
13C of VBSJ-377
157.99
142.51
140.95
131.35
130.05
129.86
129.79
129.68
129.44
127.29
125.44
123.25
118.53
117.77
116.90
116.74
113.05
77.48
77.16
76.84
OH
N
N
3ia
190 180 170 160 150 140 130 120 110 100 90 80 70 60 50 40 30 20 10 ppm
BRUKER
Current Data Parameters
NAME Dr.A.HAJRA 2017
EXPNO 1579
PROCNO 1
F2 - Acquisition Parameters
Date_ 20170917
Time 17.57
INSTRUM spect
PROBHD 5 mm PABBO BB/
PULPROG zgdc
TD 32768
SOLVENT CDCl3
NS 420
DS 2
SWH 24038.461 Hz
FIDRES 0.733596 Hz
AQ 0.6815744 sec
RG 135.7
DW 20.800 usec
DE 6.50 usec
TE 298.9 K
D1 2.00000000 sec
D11 0.03000000 sec
TD0 1
======== CHANNEL f1 ========
SFO1 100.6278588 MHz
NUC1 13C
P1 8.90 usec
PLW1 54.00000000 W
======== CHANNEL f2 ========
SFO2 400.1516006 MHz
NUC2 1H
CPDPRG[2 waltz16
PCPD2 90.00 usec
PLW2 12.00000000 W
PLW12 0.32231000 W
F2 - Processing parameters
SI 16384
SF 100.6177837 MHz
WDW EM
SSB 0
LB 1.00 Hz
GB 0
PC 1.20

1H of VBSJ-370 2nd

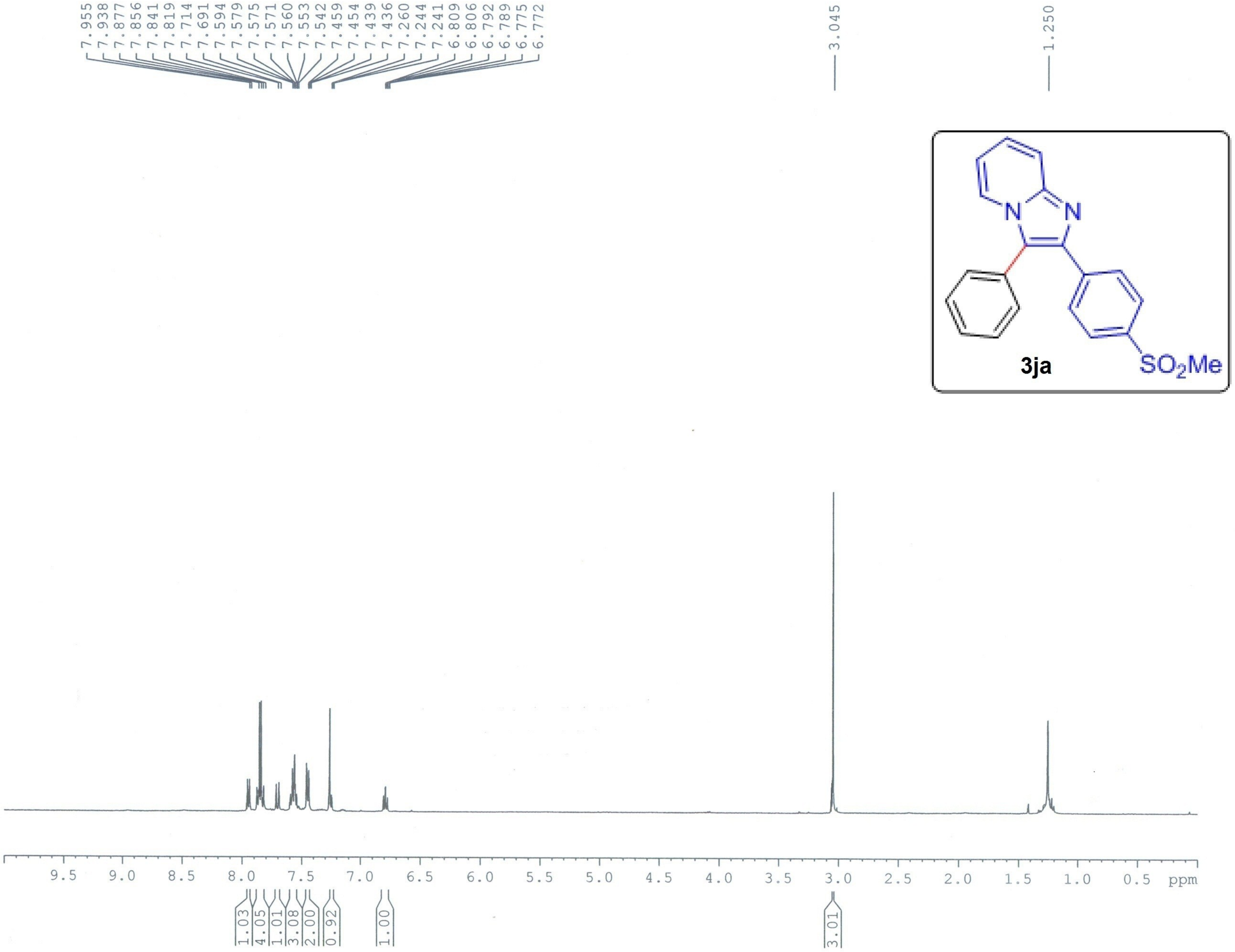

BRUKER

Current Data Parameters
NAME Dr.A.HAJRA 2018
EXPNO 297
PROCNO 1

F2 - Acquisition Parameters
Date_ 20180214
Time 19.41
INSTRUM spect
PROBHD 5 mm PABBO BB/
PULPROG zg30
TD 32768
SOLVENT CDCl3
NS 24
DS 1
SWH 8223.685 Hz
FIDRES 0.250967 Hz
AQ 1.9922944 sec
RG 186.42
DW 60.800 usec
DE 6.50 usec
TE 296.0 K
D1 1.00000000 sec
TD0 1

======== CHANNEL f1 ========
SFO1 400.1524711 MHz
NUC1 1H
P1 14.75 usec
PLW1 12.00000000 W

F2 - Processing parameters
SI 16384
SF 400.1500095 MHz
WDW EM
SSB 0
LB 0.30 Hz
GB 0
PC 1.00

13C of VBSJ-370 2nd

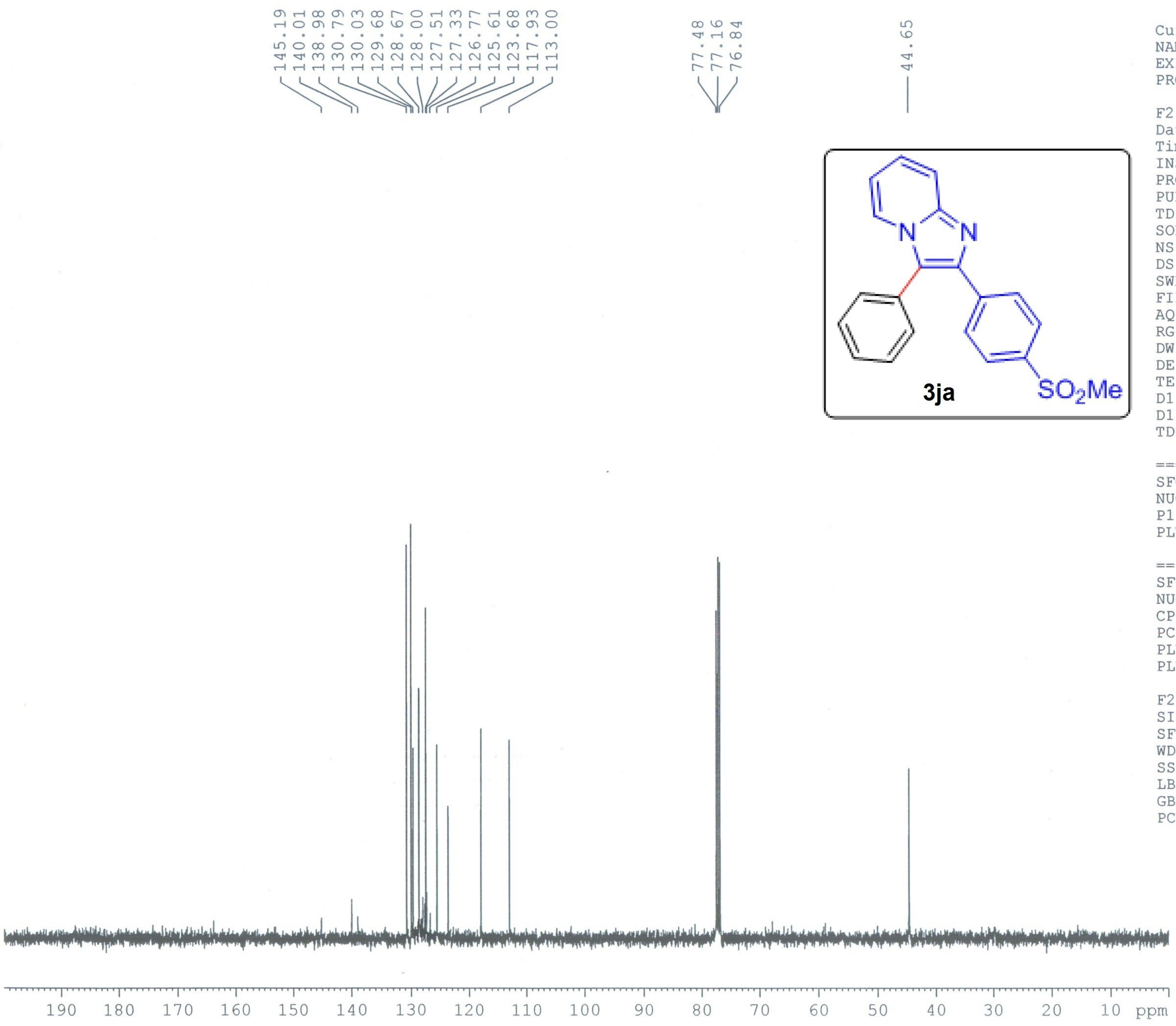

BRUKER

Current Data Parameters
NAME Dr.A.HAJRA 2018
EXPNO 302
PROCNO 1

F2 - Acquisition Parameters
Date_ 20180215
Time 11.14
INSTRUM spect
PROBHD 5 mm PABBO BB/
PULPROG zgdc
TD 32768
SOLVENT CDCl3
NS 640
DS 2
SWH 24038.461 Hz
FIDRES 0.733596 Hz
AQ 0.6815744 sec
RG 135.7
DW 20.800 usec
DE 6.50 usec
TE 294.8 K
D1 2.00000000 sec
D11 0.03000000 sec
TD0 1

======== CHANNEL f1 ========
SFO1 100.6278588 MHz
NUC1 13C
P1 8.90 usec
PLW1 54.00000000 W

======== CHANNEL f2 ========
SFO2 400.1516006 MHz
NUC2 1H
CPDPRG[2 waltz16
PCPD2 90.00 usec
PLW2 12.00000000 W
PLW12 0.32231000 W

F2 - Processing parameters
SI 16384
SF 100.6177843 MHz
WDW EM
SSB 0
LB 1.00 Hz
GB 0
PC 1.00

1H of VBSJ-381

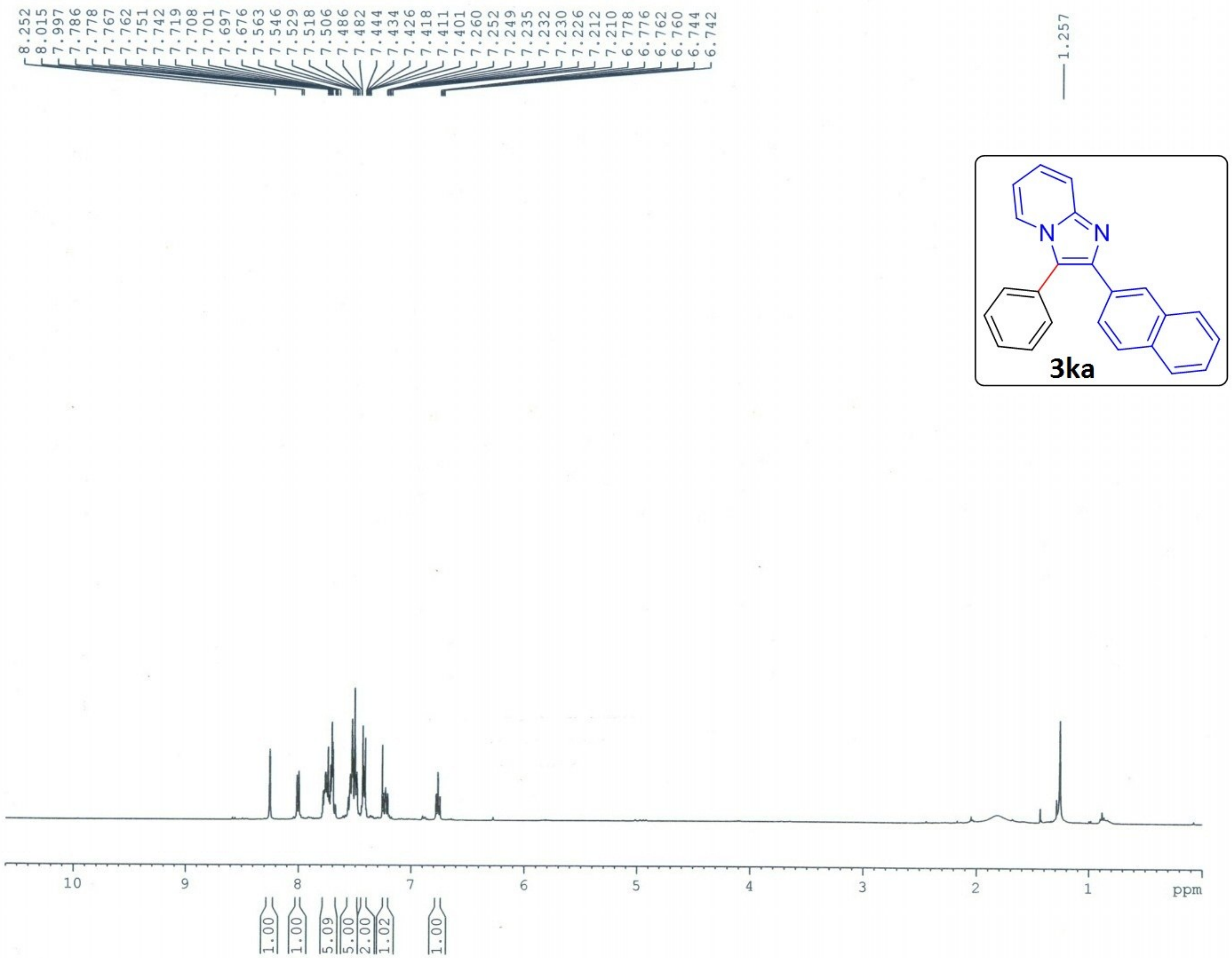

3ka

Current Data Parameters
NAME Dr.A.HAJRA 2017
EXPNO 1611
PROCNO 1

F2 - Acquisition Parameters
Date_ 20170922
Time 10.44
INSTRUM spect
PROBHD 5 mm PABBO BB/
PULPROG zg30
TD 32768
SOLVENT CDCl3
NS 24
DS 1
SWH 8223.685 Hz
FIDRES 0.250967 Hz
AQ 1.9922944 sec
RG 135.7
DW 60.800 usec
DE 6.50 usec
TE 297.4 K
D1 1.00000000 sec
TD0 1

======== CHANNEL f1 ========
SFO1 400.1524711 MHz
NUC1 1H
P1 14.75 usec
PLW1 12.00000000 W

F2 - Processing parameters
SI 16384
SF 400.1500095 MHz
WDW EM
SSB 0
LB 0.30 Hz
GB 0
PC 1.00

13C of VBSJ-381

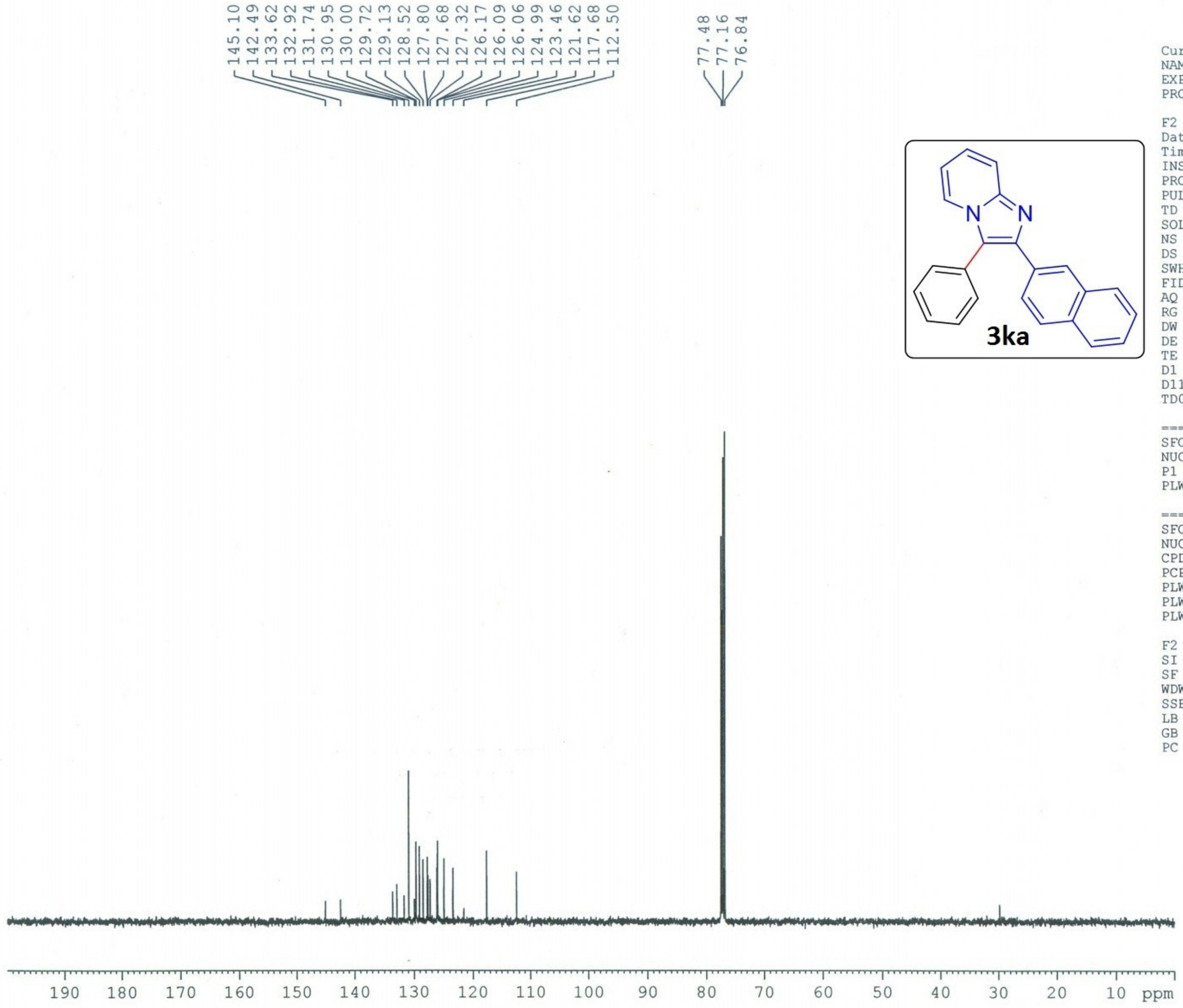

3ka

```
Current Data Parameters
NAME       Dr.A.HAJRA 2017
EXPNO                1612
PROCNO                  1

F2 - Acquisition Parameters
Date_            20170922
Time                11.09
INSTRUM             spect
PROBHD    5 mm PABBO BB/
PULPROG           zgpg30
TD                  32768
SOLVENT             CDCl3
NS                    400
DS                      2
SWH             24038.461 Hz
FIDRES           0.733596 Hz
AQ              0.6815744 sec
RG                  135.7
DW                 20.800 usec
DE                   6.50 usec
TE                  298.2 K
D1             2.00000000 sec
D11            0.03000000 sec
TD0                     1

======== CHANNEL f1 ========
SFO1          100.6278588 MHz
NUC1                  13C
P1                   8.90 usec
PLW1          54.00000000 W

======== CHANNEL f2 ========
SFO2          400.1516006 MHz
NUC2                   1H
CPDPRG[2         waltz16
PCPD2               90.00 usec
PLW2          12.00000000 W
PLW12          0.32231000 W
PLW13          0.16212000 W

F2 - Processing parameters
SI                  16384
SF            100.6177849 MHz
WDW                    EM
SSB       0
LB                   1.00 Hz
GB        0
PC                   1.20
```

1H of VBSJ-387

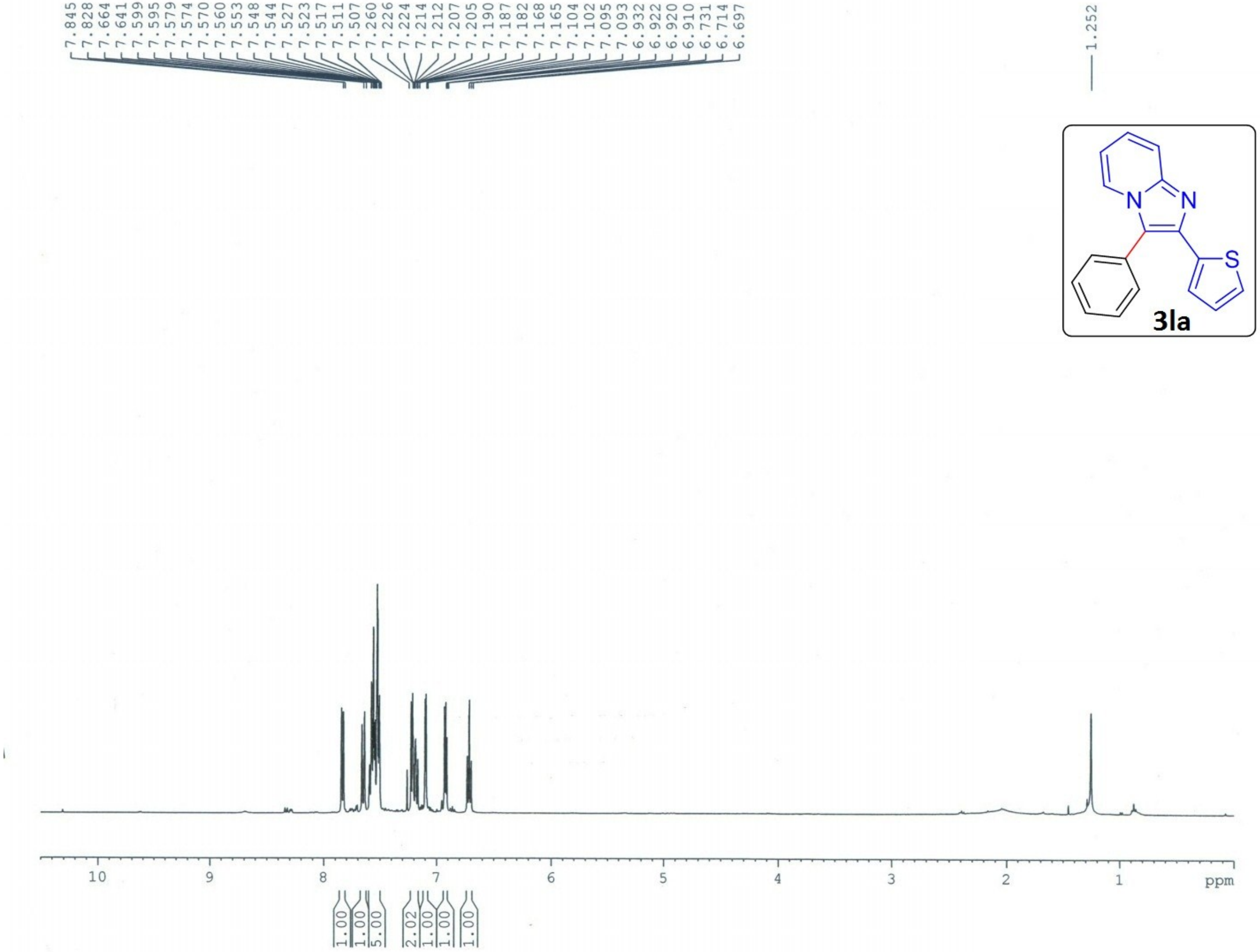

3la

BRUKER

Current Data Parameters
NAME Dr.A.HAJRA 2017
EXPNO 1634
PROCNO 1

F2 - Acquisition Parameters
Date_ 20171008
Time 10.09
INSTRUM spect
PROBHD 5 mm PABBO BB/
PULPROG zg30
TD 32768
SOLVENT CDCl3
NS 20
DS 1
SWH 8223.685 Hz
FIDRES 0.250967 Hz
AQ 1.9922944 sec
RG 106.66
DW 60.800 usec
DE 6.50 usec
TE 296.0 K
D1 1.00000000 sec
TD0 1

======== CHANNEL f1 ========
SFO1 400.1524711 MHz
NUC1 1H
P1 14.75 usec
PLW1 12.00000000 W

F2 - Processing parameters
SI 16384
SF 400.1500096 MHz
WDW EM
SSB 0
LB 0.30 Hz
GB 0
PC 1.00

13C of VBSJ-387

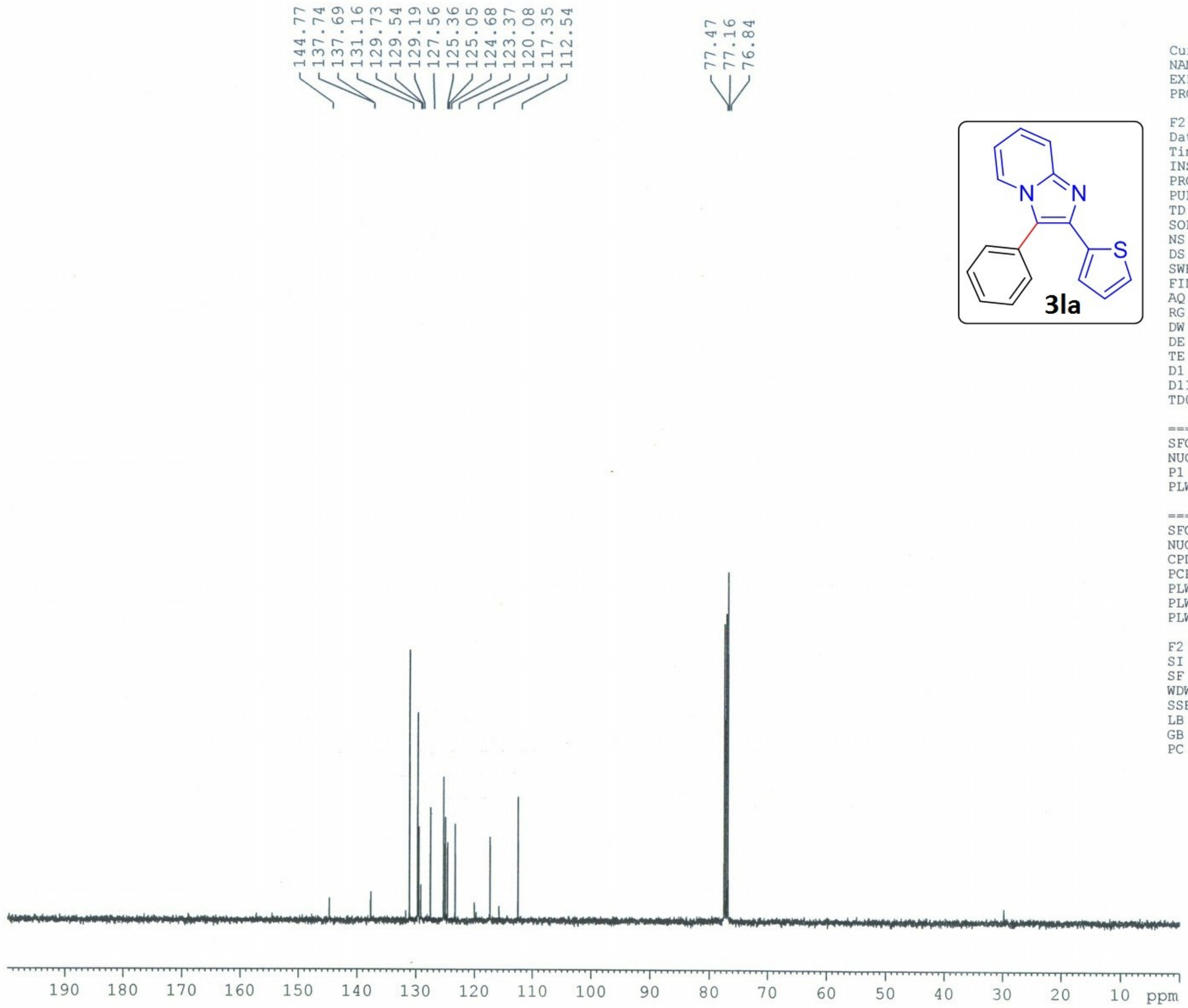

3la

BRUKER

Current Data Parameters
NAME Dr.A.HAJRA 2017
EXPNO 1635
PROCNO 1

F2 - Acquisition Parameters
Date_ 20171008
Time 11.58
INSTRUM spect
PROBHD 5 mm PABBO BB/
PULPROG zgpg30
TD 32768
SOLVENT CDCl3
NS 200
DS 2
SWH 24038.461 Hz
FIDRES 0.733596 Hz
AQ 0.6815744 sec
RG 106.66
DW 20.800 usec
DE 6.50 usec
TE 296.4 K
D1 2.00000000 sec
D11 0.03000000 sec
TD0 1

======== CHANNEL f1 ========
SFO1 100.6278588 MHz
NUC1 13C
P1 8.90 usec
PLW1 54.00000000 W

======== CHANNEL f2 ========
SFO2 400.1516006 MHz
NUC2 1H
CPDPRG[2 waltz16
PCPD2 90.00 usec
PLW2 12.00000000 W
PLW12 0.32231000 W
PLW13 0.16212000 W

F2 - Processing parameters
SI 16384
SF 100.6177876 MHz
WDW EM
SSB 0
LB 1.00 Hz
GB 0
PC 1.40

1H of VBSJ-400

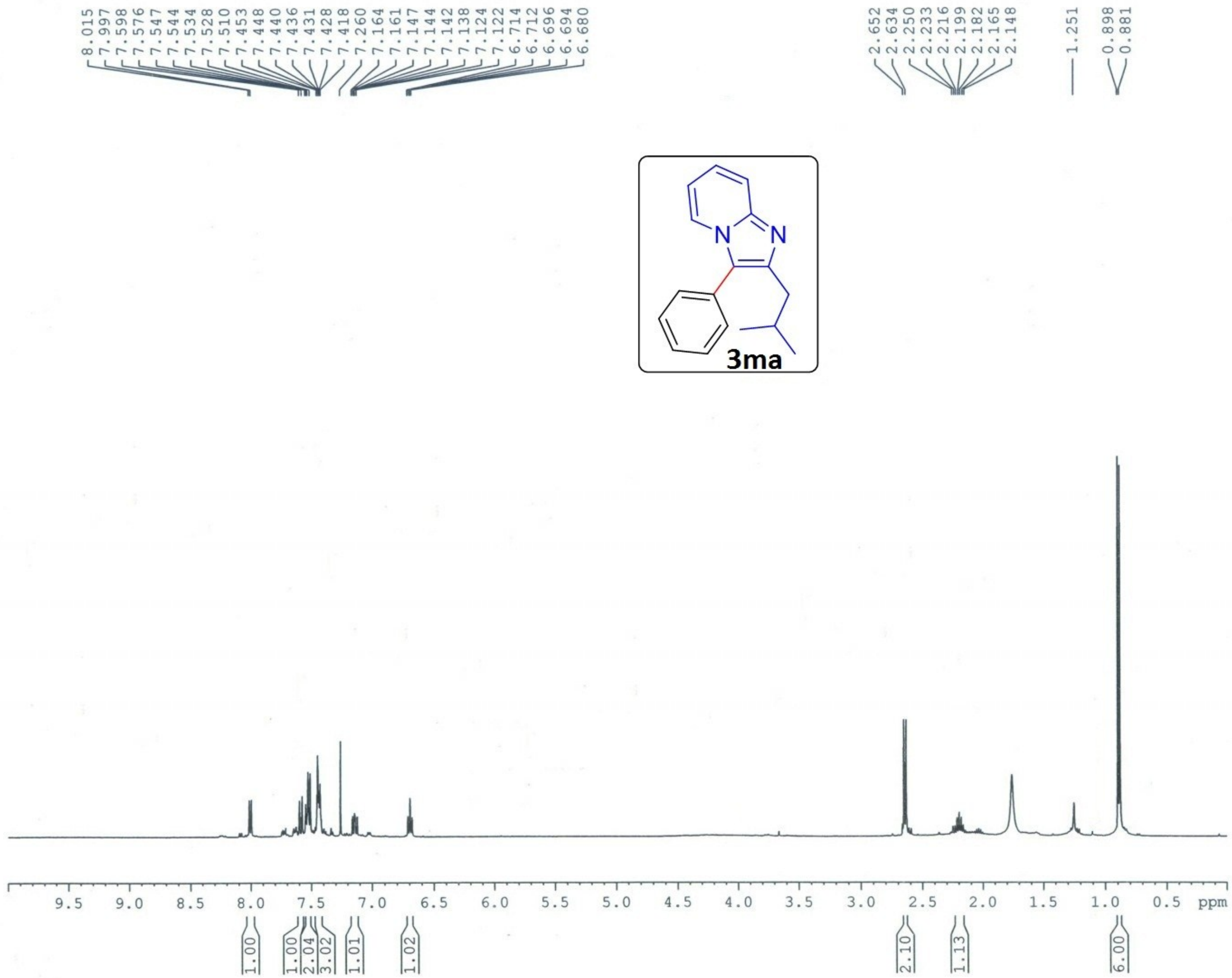

Current Data Parameters
NAME Dr.A.HAJRA 2017
EXPNO 1727
PROCNO 1

F2 - Acquisition Parameters
Date_ 20171026
Time 16.14
INSTRUM spect
PROBHD 5 mm PABBO BB/
PULPROG zg30
TD 32768
SOLVENT CDCl3
NS 32
DS 1
SWH 8223.685 Hz
FIDRES 0.250967 Hz
AQ 1.9922944 sec
RG 168.31
DW 60.800 usec
DE 6.50 usec
TE 296.7 K
D1 1.00000000 sec
TD0 1

======== CHANNEL f1 ========
SFO1 400.1524711 MHz
NUC1 1H
P1 14.75 usec
PLW1 12.00000000 W

F2 - Processing parameters
SI 16384
SF 400.1500095 MHz
WDW EM
SSB 0
LB 0.30 Hz
GB 0
PC 1.00

13C of VBSJ-400

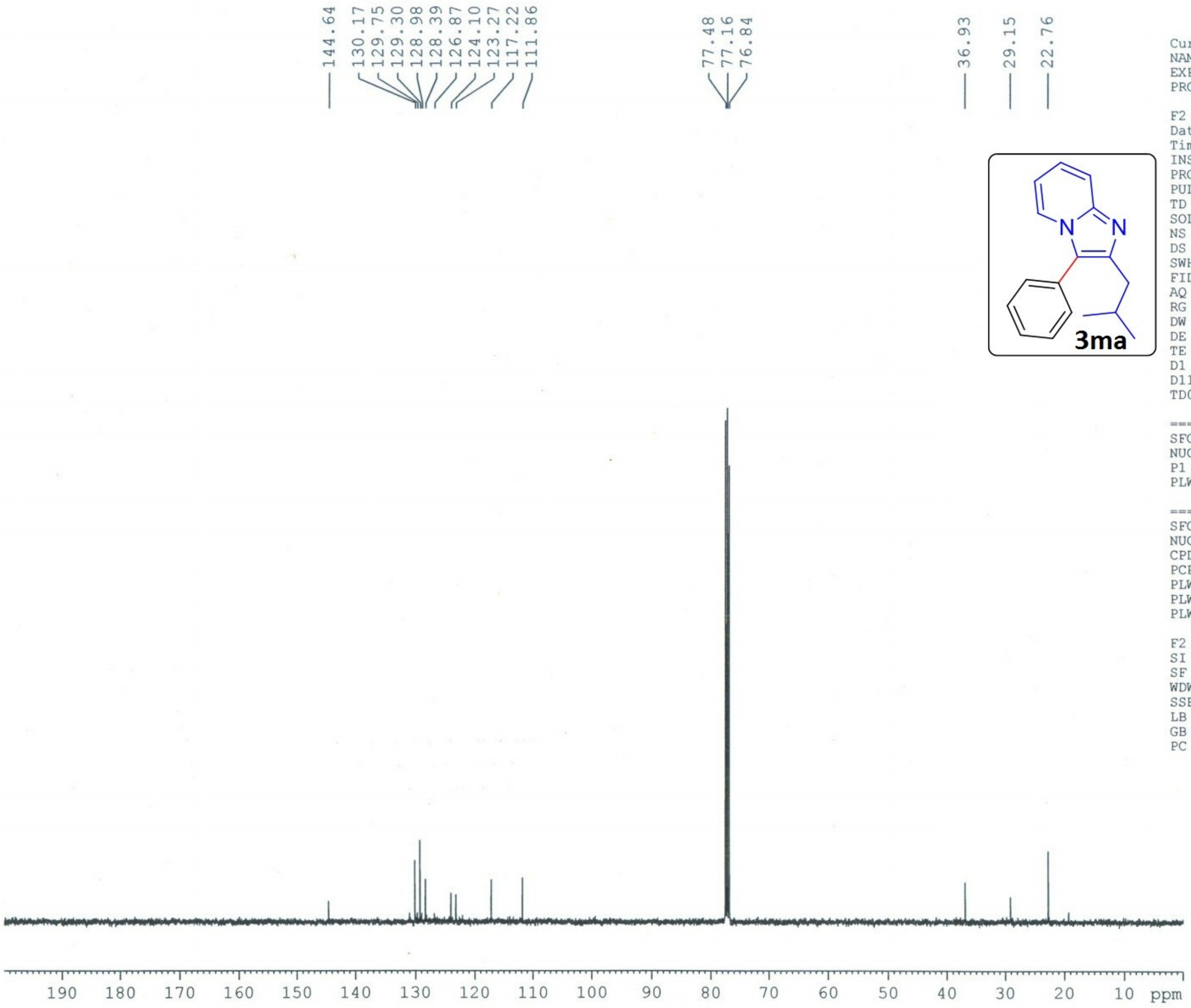

BRUKER

Current Data Parameters
NAME Dr.A.HAJRA 2017
EXPNO 1730
PROCNO 1

F2 - Acquisition Parameters
Date_ 20171026
Time 16.30
INSTRUM spect
PROBHD 5 mm PABBO BB/
PULPROG zgpg30
TD 32768
SOLVENT CDCl3
NS 640
DS 2
SWH 24038.461 Hz
FIDRES 0.733596 Hz
AQ 0.6815744 sec
RG 168.31
DW 20.800 usec
DE 6.50 usec
TE 297.1 K
D1 2.00000000 sec
D11 0.03000000 sec
TD0 1

======== CHANNEL f1 ========
SFO1 100.6278588 MHz
NUC1 13C
P1 8.90 usec
PLW1 54.00000000 W

======== CHANNEL f2 ========
SFO2 400.1516006 MHz
NUC2 1H
CPDPRG[2 waltz16
PCPD2 90.00 usec
PLW2 12.00000000 W
PLW12 0.32231000 W
PLW13 0.16212000 W

F2 - Processing parameters
SI 16384
SF 100.6177837 MHz
WDW EM
SSB 0
LB 1.00 Hz
GB 0
PC 1.20

1H of VBSJ-3Phenyl

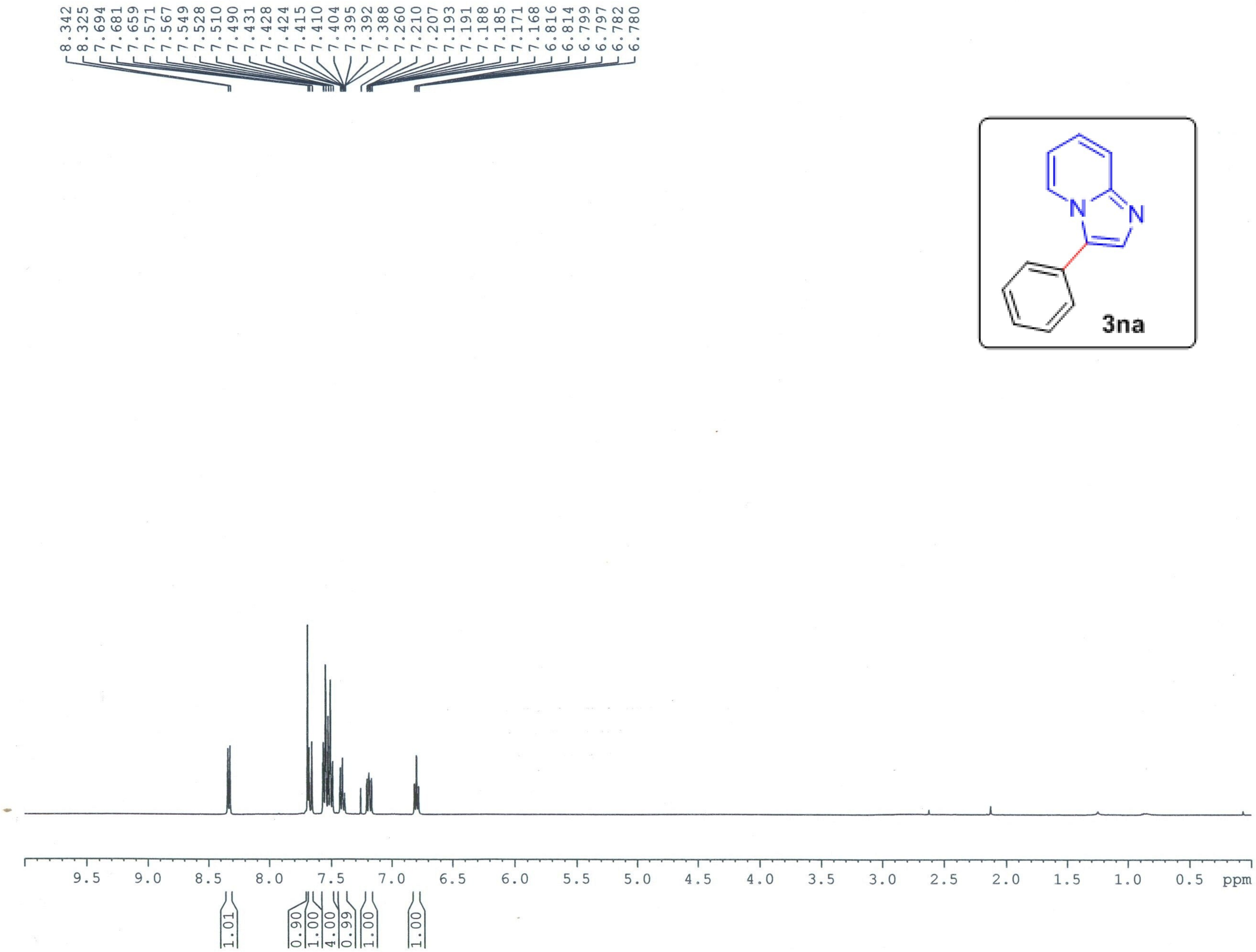

3na

Current Data Parameters
NAME Dr. A HAJRA 2018-2nd
EXPNO 64
PROCNO 1

F2 - Acquisition Parameters
Date_ 20180313
Time 17.25
INSTRUM spect
PROBHD 5 mm PABBO BB/
PULPROG zg30
TD 32768
SOLVENT CDCl3
NS 8
DS 2
SWH 8223.685 Hz
FIDRES 0.250967 Hz
AQ 1.9922944 sec
RG 87.66
DW 60.800 usec
DE 6.50 usec
TE 296.8 K
D1 1.00000000 sec
TD0 1

======== CHANNEL f1 ========
SFO1 400.1524711 MHz
NUC1 1H
P1 14.75 usec
PLW1 12.00000000 W

F2 - Processing parameters
SI 16384
SF 400.1500096 MHz
WDW EM
SSB 0
LB 0.30 Hz
GB 0
PC 1.00

13C of VBSJ- 3Phenyl

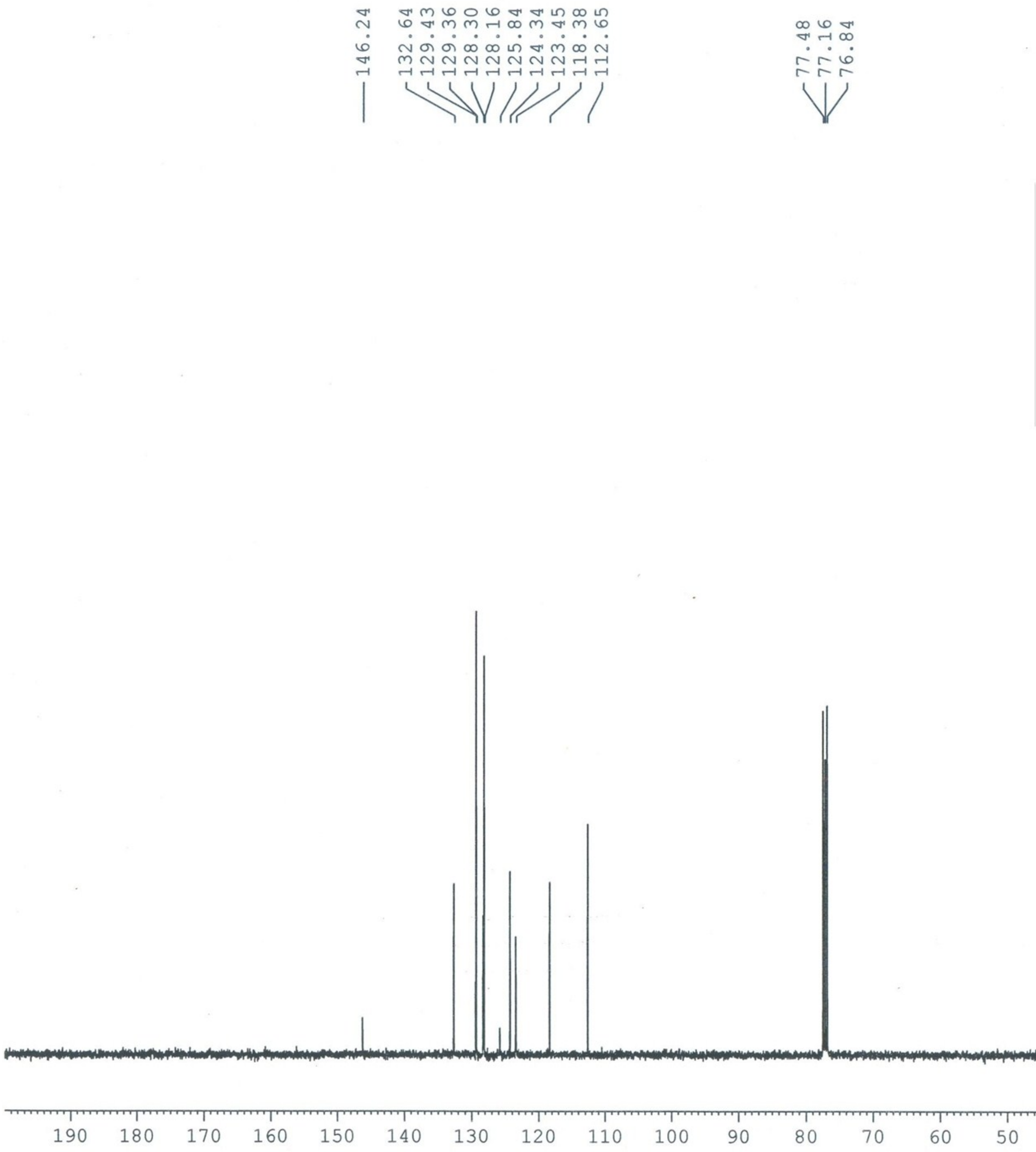

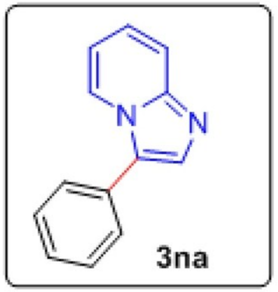

BRUKER

```
Current Data Parameters
NAME      Dr. A HAJRA 2018-2nd
EXPNO                       65
PROCNO                       1

F2 - Acquisition Parameters
Date_                 20180313
Time                     17.35
INSTRUM                  spect
PROBHD    5 mm PABBO BB/
PULPROG                zgpg30
TD                       32768
SOLVENT                  CDCl3
NS                         160
DS                           2
SWH              24038.461 Hz
FIDRES            0.733596 Hz
AQ               0.6815744 sec
RG                       87.66
DW                  20.800 usec
DE                    6.50 usec
TE                     297.3 K
D1              2.00000000 sec
D11             0.03000000 sec
TD0                          1

======== CHANNEL f1 ========
SFO1         100.6278588 MHz
NUC1                     13C
P1                    8.90 usec
PLW1           54.00000000 W

======== CHANNEL f2 ========
SFO2         400.1516006 MHz
NUC2                      1H
CPDPRG[2             waltz16
PCPD2                 90.00 usec
PLW2           12.00000000 W
PLW12           0.32231000 W
PLW13           0.16212000 W

F2 - Processing parameters
SI                     16384
SF           100.6177873 MHz
WDW                       EM
SSB       0
LB                    1.00 Hz
GB        0
PC                      1.40
```

1H of of VBSJ-350

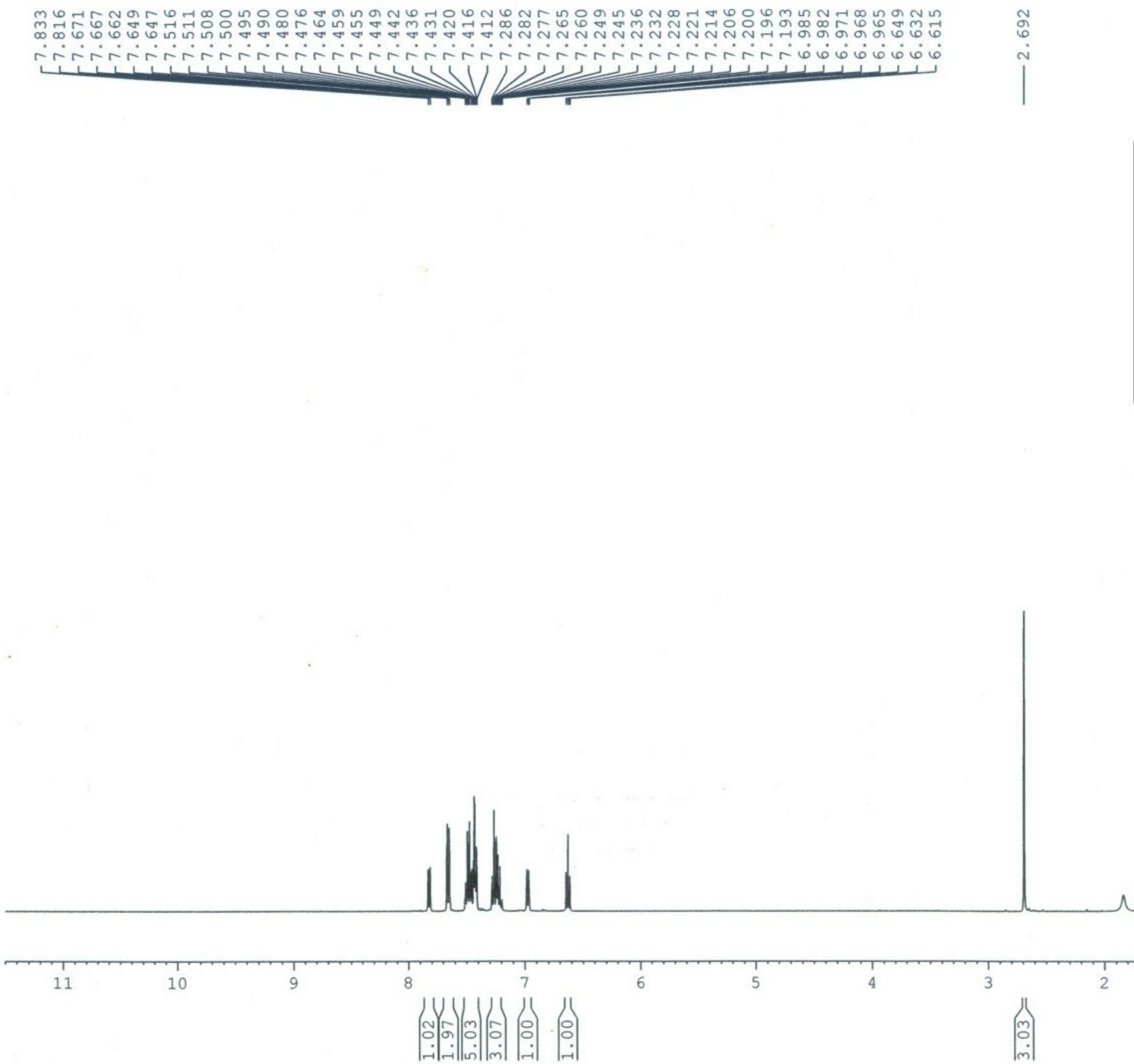

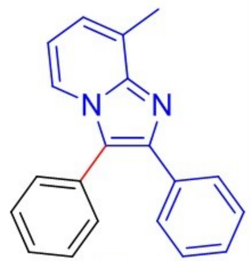

3oa

BRUKER

Current Data Parameters

NAME	Dr.A.HAJRA 2017
EXPNO	1383
PROCNO	1

F2 - Acquisition Parameters

Date_	20170810
Time	19.45
INSTRUM	spect
PROBHD	5 mm PABBO BB/
PULPROG	zg30
TD	32768
SOLVENT	CDCl3
NS	16
DS	1
SWH	8223.685 Hz
FIDRES	0.250967 Hz
AQ	1.9922944 sec
RG	93.46
DW	60.800 usec
DE	6.50 usec
TE	299.2 K
D1	1.00000000 sec
TD0	1

======== CHANNEL f1 ========

SFO1	400.1524711 MHz
NUC1	1H
P1	14.75 usec
PLW1	12.00000000 W

F2 - Processing parameters

SI	16384
SF	400.1500163 MHz
WDW	EM
SSB	0
LB	0.30 Hz
GB	0
PC	1.00

13C of VBSJ-350

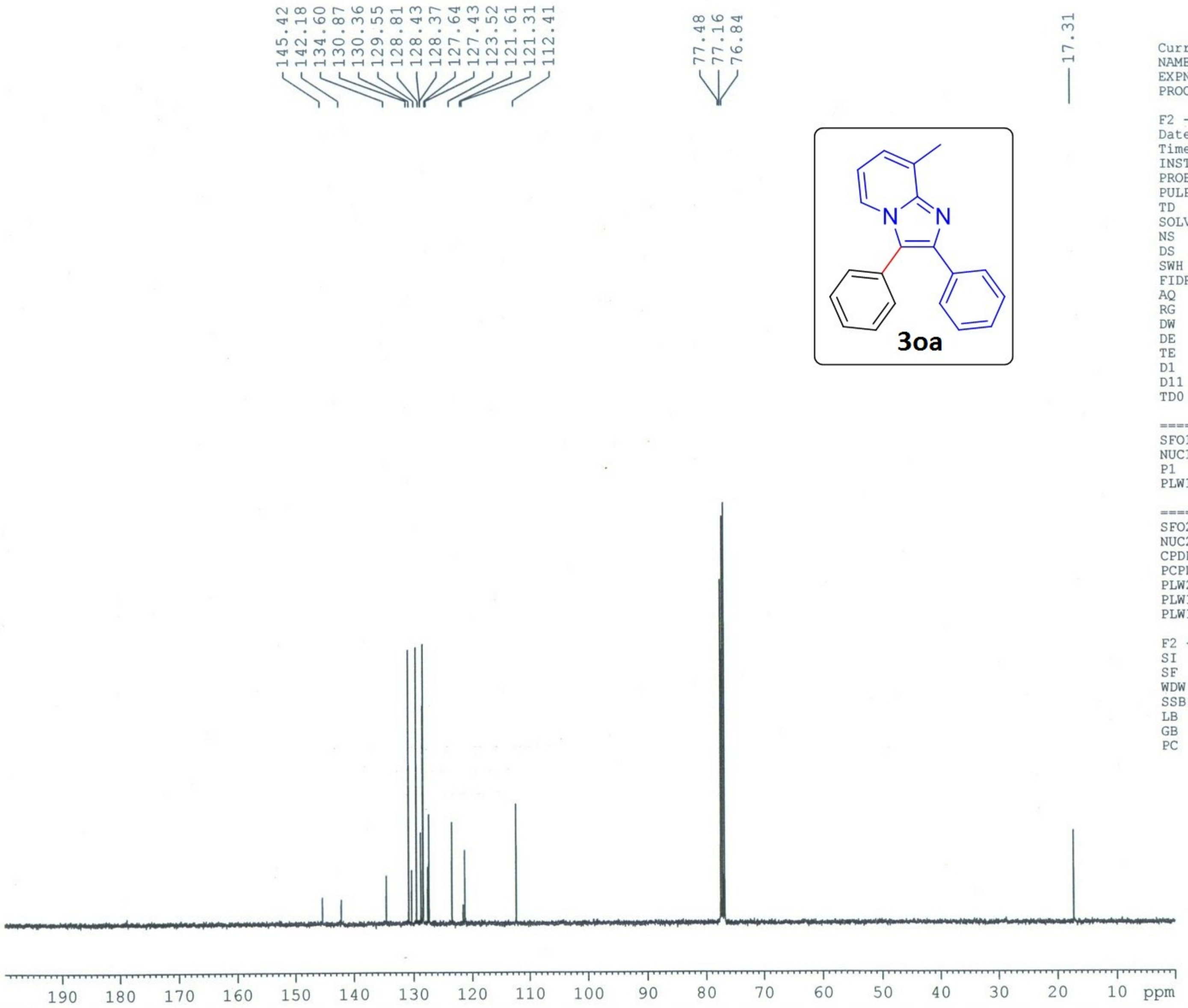

BRUKER

Current Data Parameters
NAME Dr.A.HAJRA 2017
EXPNO 1384
PROCNO 1

F2 - Acquisition Parameters
Date_ 20170810
Time 22.16
INSTRUM spect
PROBHD 5 mm PABBO BB/
PULPROG zgpg30
TD 32768
SOLVENT CDCl3
NS 640
DS 2
SWH 24038.461 Hz
FIDRES 0.733596 Hz
AQ 0.6815744 sec
RG 93.46
DW 20.800 usec
DE 6.50 usec
TE 298.8 K
D1 2.00000000 sec
D11 0.03000000 sec
TD0 1

======== CHANNEL f1 ========
SFO1 100.6278588 MHz
NUC1 13C
P1 8.90 usec
PLW1 54.00000000 W

======== CHANNEL f2 ========
SFO2 400.1516006 MHz
NUC2 1H
CPDPRG[2 waltz16
PCPD2 90.00 usec
PLW2 12.00000000 W
PLW12 0.32231000 W
PLW13 0.16212000 W

F2 - Processing parameters
SI 16384
SF 100.6177849 MHz
WDW EM
SSB 0
LB 1.00 Hz
GB 0
PC 1.40

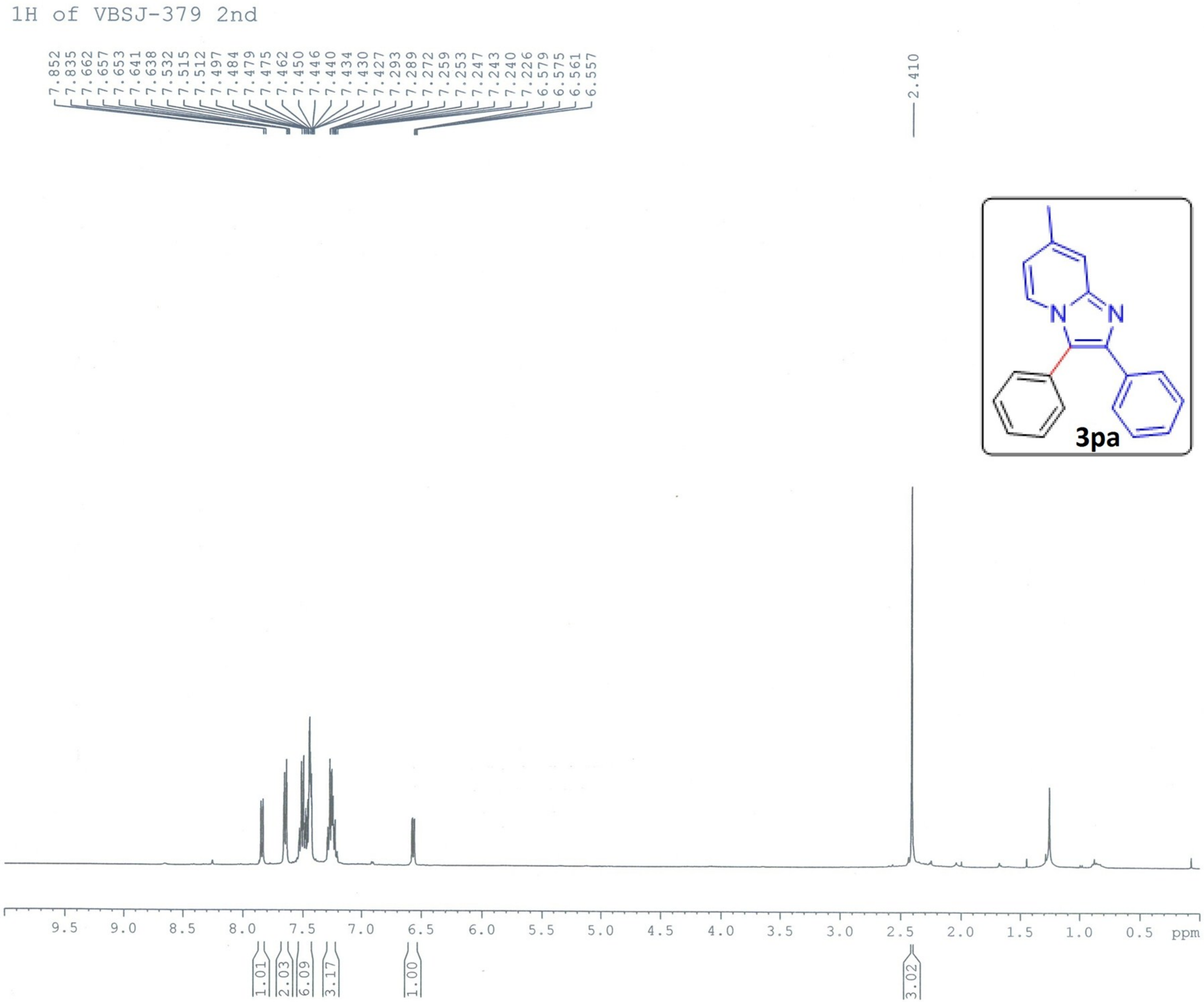

BRUKER

```
Current Data Parameters
NAME       Dr.A.HAJRA 2018
EXPNO                  300
PROCNO                   1

F2 - Acquisition Parameters
Date_             20180214
Time                 20.26
INSTRUM              spect
PROBHD   5 mm PABBO BB/
PULPROG               zg30
TD                   32768
SOLVENT              CDCl3
NS                       8
DS                       1
SWH               8223.685 Hz
FIDRES            0.250967 Hz
AQ               1.9922944 sec
RG                   93.46
DW                  60.800 usec
DE                    6.50 usec
TE                   296.0 K
D1              1.00000000 sec
TD0                      1

======== CHANNEL f1 ========
SFO1        400.1524711 MHz
NUC1                    1H
P1                   14.75 usec
PLW1           12.00000000 W

F2 - Processing parameters
SI                   16384
SF          400.1500097 MHz
WDW                     EM
SSB      0
LB                    0.30 Hz
GB       0
PC                    1.00
```

13C of VBSJ-379

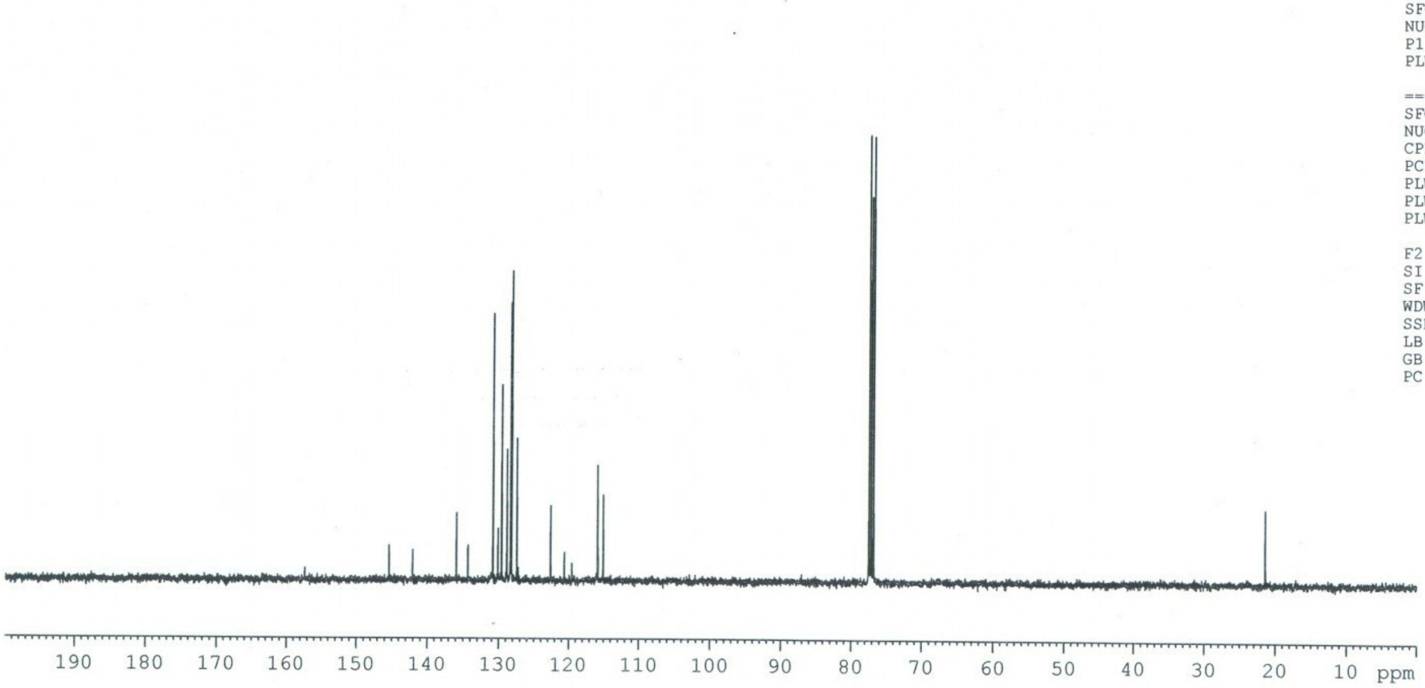

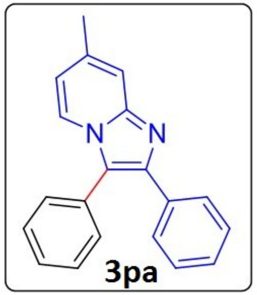

BRUKER

Current Data Parameters
NAME Dr.A.HAJRA 2017
EXPNO 1601
PROCNO 1

F2 - Acquisition Parameters
Date_ 20170920
Time 17.35
INSTRUM spect
PROBHD 5 mm PABBO BB/
PULPROG zgpg30
TD 32768
SOLVENT CDCl3
NS 352
DS 2
SWH 24038.461 Hz
FIDRES 0.733596 Hz
AQ 0.6815744 sec
RG 120.16
DW 20.800 usec
DE 6.50 usec
TE 298.0 K
D1 2.00000000 sec
D11 0.03000000 sec
TD0 1

======== CHANNEL f1 ========
SFO1 100.6278588 MHz
NUC1 13C
P1 8.90 usec
PLW1 54.00000000 W

======== CHANNEL f2 ========
SFO2 400.1516006 MHz
NUC2 1H
CPDPRG[2 waltz16
PCPD2 90.00 usec
PLW2 12.00000000 W
PLW12 0.32231000 W
PLW13 0.16212000 W

F2 - Processing parameters
SI 16384
SF 100.6177857 MHz
WDW EM
SSB 0
LB 1.00 Hz
GB 0
PC 1.20

1H of VBSJ-394

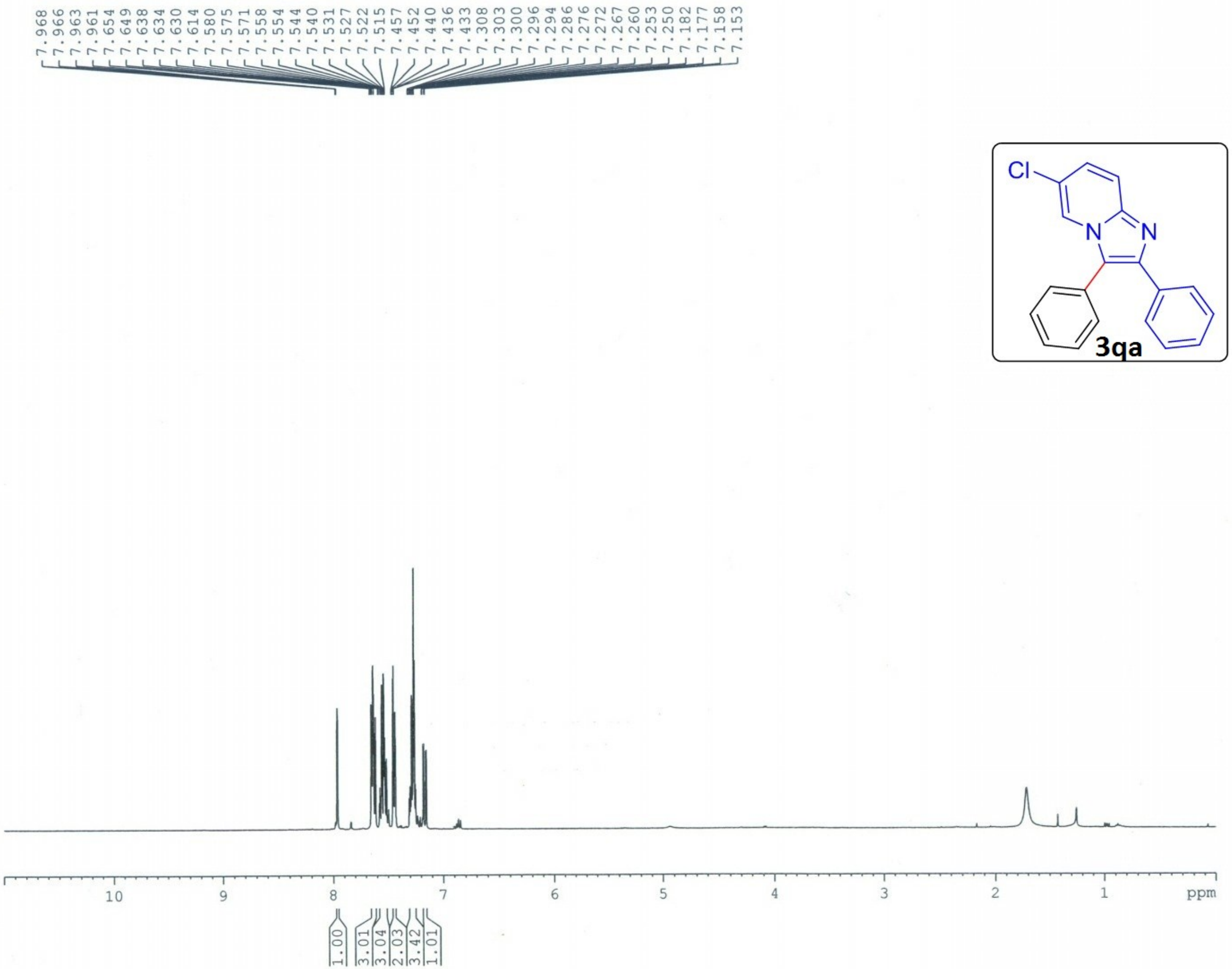

Current Data Parameters
NAME Dr.A.HAJRA 2017
EXPNO 1650
PROCNO 1

F2 - Acquisition Parameters
Date_ 20171009
Time 20.10
INSTRUM spect
PROBHD 5 mm PABBO BB/
PULPROG zg30
TD 32768
SOLVENT CDCl3
NS 32
DS 1
SWH 8223.685 Hz
FIDRES 0.250967 Hz
AQ 1.9922944 sec
RG 106.66
DW 60.800 usec
DE 6.50 usec
TE 297.8 K
D1 1.00000000 sec
TD0 1

======== CHANNEL f1 ========
SFO1 400.1524711 MHz
NUC1 1H
P1 14.75 usec
PLW1 12.00000000 W

F2 - Processing parameters
SI 16384
SF 400.1500095 MHz
WDW EM
SSB 0
LB 0.30 Hz
GB 0
PC 1.00

13C of VBSJ-394

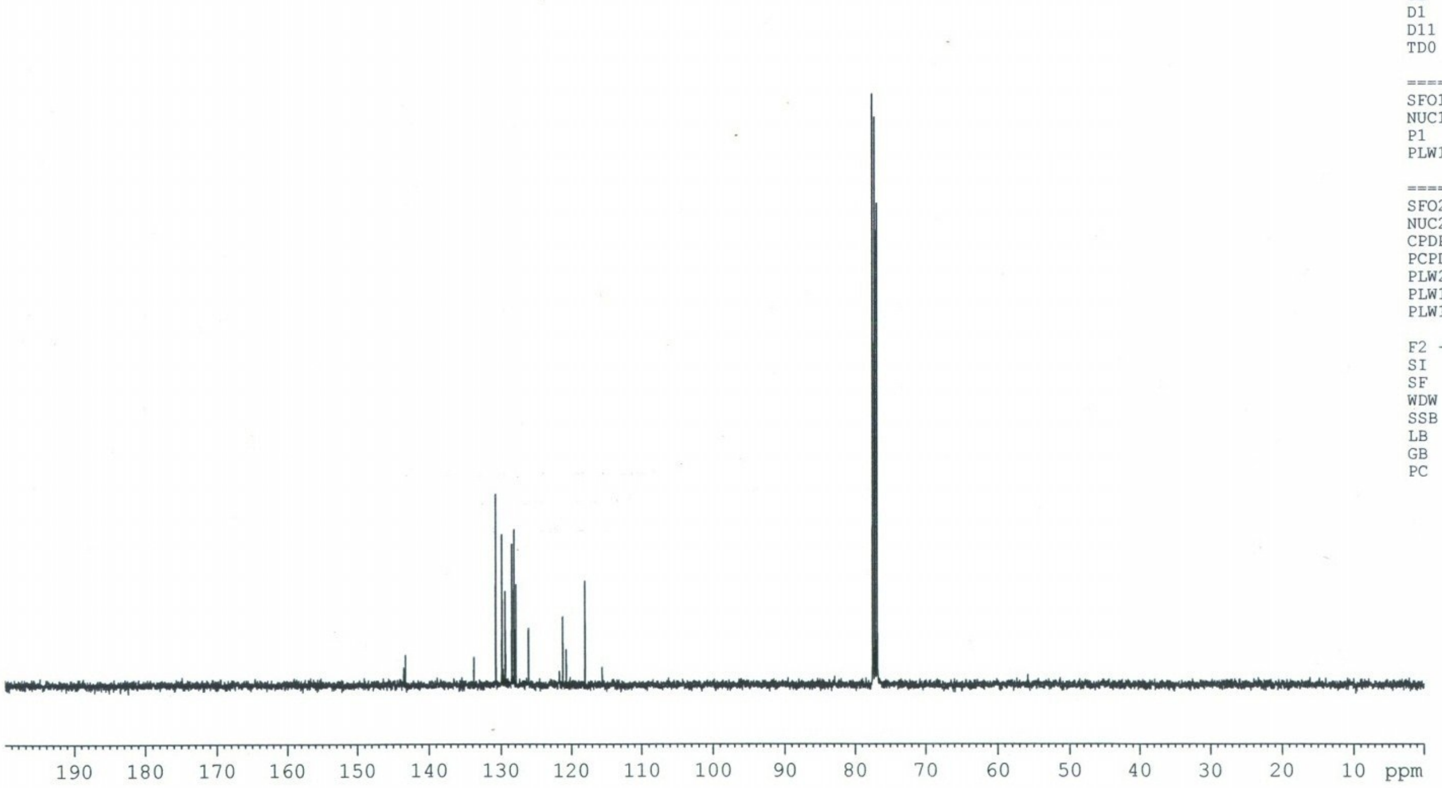

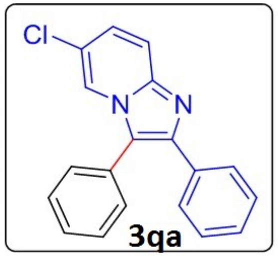

3qa

BRUKER

Current Data Parameters
NAME Dr.A.HAJRA 2017
EXPNO 1651
PROCNO 1

F2 - Acquisition Parameters
Date_ 20171009
Time 20.28
INSTRUM spect
PROBHD 5 mm PABBO BB/
PULPROG zgpg30
TD 32768
SOLVENT CDCl3
NS 400
DS 2
SWH 24038.461 Hz
FIDRES 0.733596 Hz
AQ 0.6815744 sec
RG 106.66
DW 20.800 usec
DE 6.50 usec
TE 298.3 K
D1 2.00000000 sec
D11 0.03000000 sec
TD0 1

======== CHANNEL f1 ========
SFO1 100.6278588 MHz
NUC1 13C
P1 8.90 usec
PLW1 54.00000000 W

======== CHANNEL f2 ========
SFO2 400.1516006 MHz
NUC2 1H
CPDPRG[2 waltz16
PCPD2 90.00 usec
PLW2 12.00000000 W
PLW12 0.32231000 W
PLW13 0.16212000 W

F2 - Processing parameters
SI 16384
SF 100.6177838 MHz
WDW EM
SSB 0
LB 1.00 Hz
GB 0
PC 1.40

1H of VBSJ-395

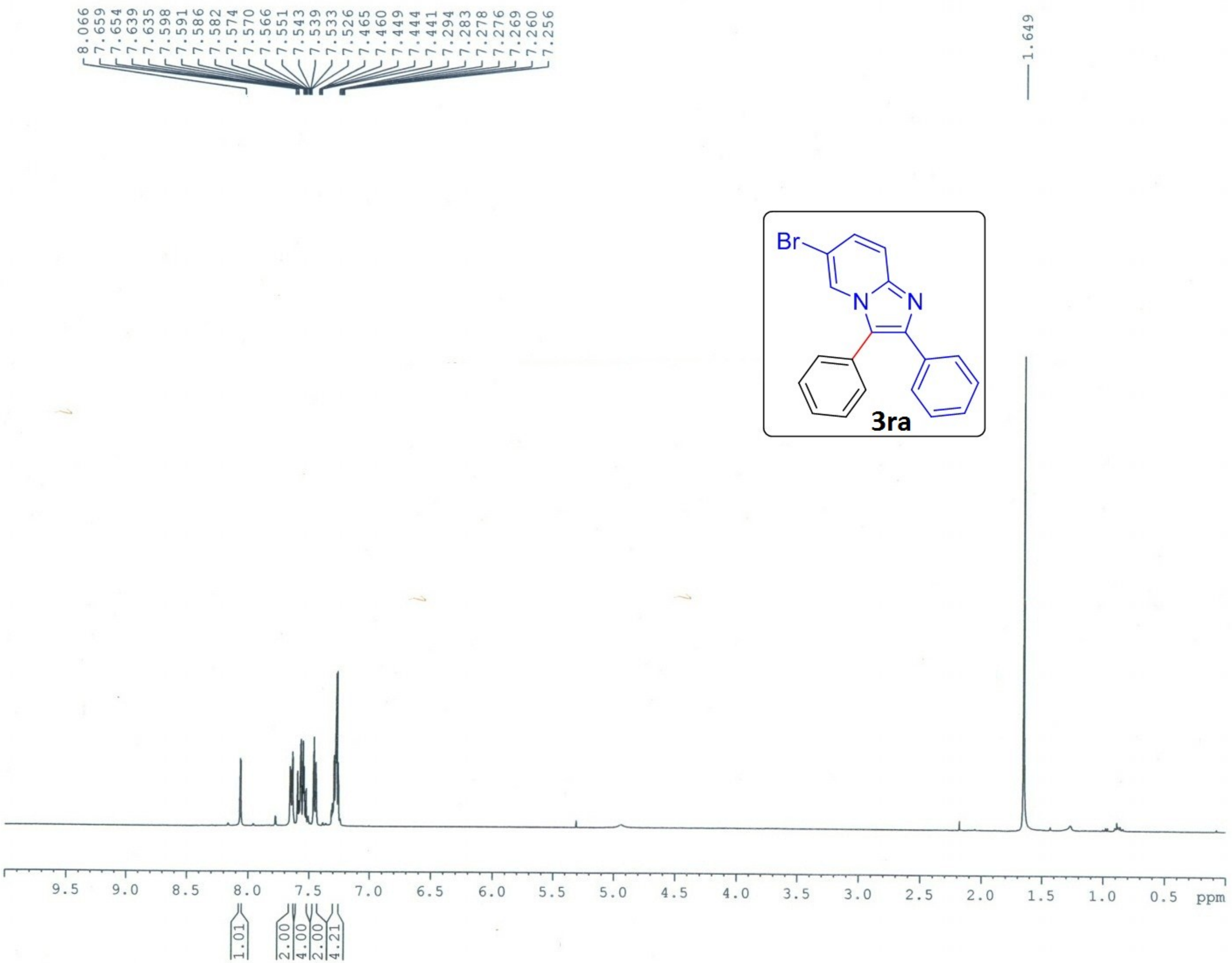

BRUKER

Current Data Parameters
NAME Dr.A.HAJRA 2017
EXPNO 1655
PROCNO 1

F2 - Acquisition Parameters
Date_ 20171011
Time 12.42
INSTRUM spect
PROBHD 5 mm PABBO BB/
PULPROG zg30
TD 32768
SOLVENT CDCl3
NS 32
DS 1
SWH 8223.685 Hz
FIDRES 0.250967 Hz
AQ 1.9922944 sec
RG 186.42
DW 60.800 usec
DE 6.50 usec
TE 296.0 K
D1 1.00000000 sec
TD0 1

======== CHANNEL f1 ========
SFO1 400.1524711 MHz
NUC1 1H
P1 14.75 usec
PLW1 12.00000000 W

F2 - Processing parameters
SI 16384
SF 400.1500061 MHz
WDW EM
SSB 0
LB 0.30 Hz
GB 0
PC 1.00

13C of VBSJ- 395

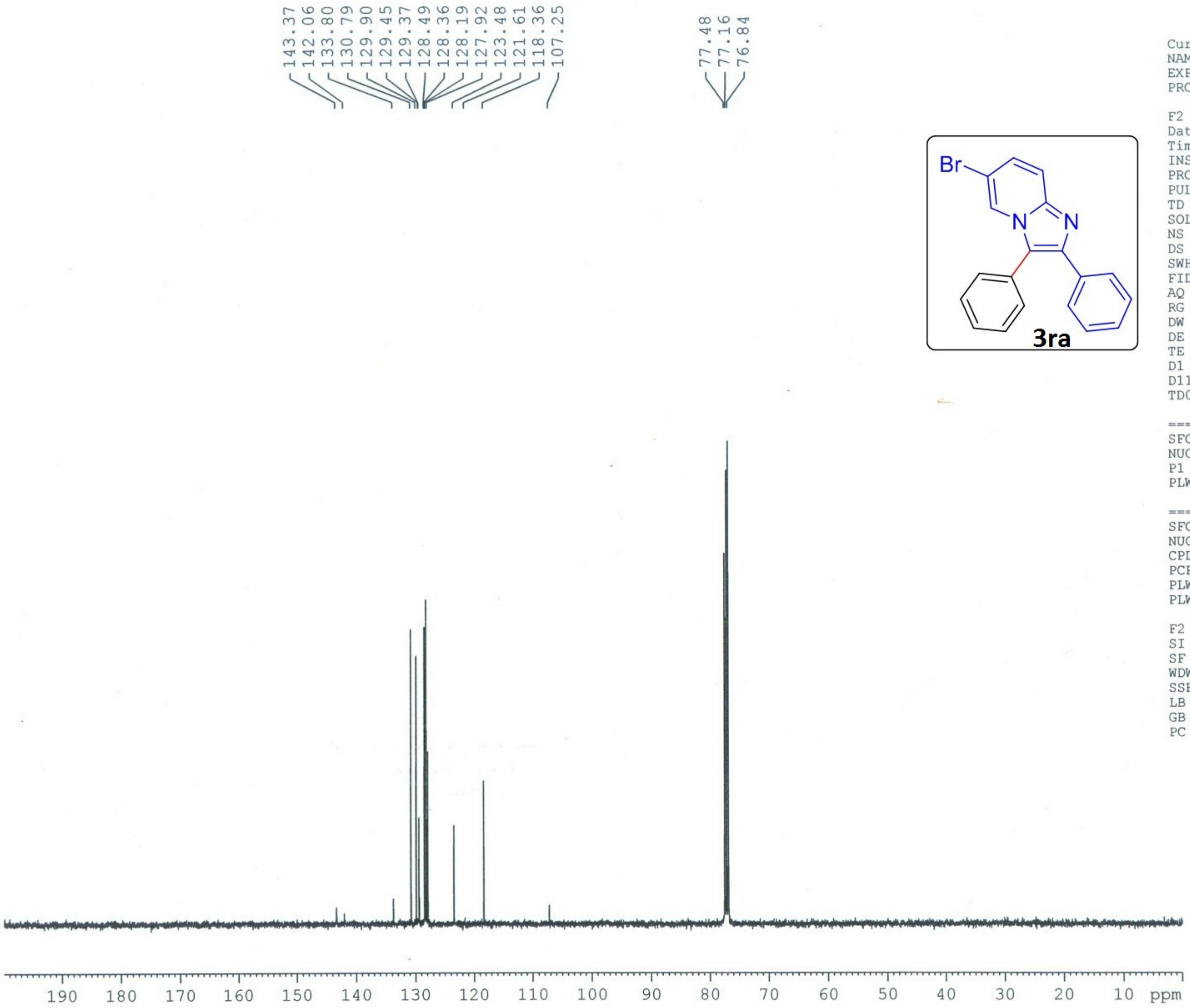

3ra

BRUKER

Current Data Parameters
NAME Dr.A.HAJRA 2017
EXPNO 1656
PROCNO 1

F2 - Acquisition Parameters
Date_ 20171011
Time 23.29
INSTRUM spect
PROBHD 5 mm PABBO BB/
PULPROG zgdc
TD 32768
SOLVENT CDCl3
NS 3072
DS 2
SWH 24038.461 Hz
FIDRES 0.733596 Hz
AQ 0.6815744 sec
RG 186.42
DW 20.800 usec
DE 6.50 usec
TE 297.4 K
D1 2.00000000 sec
D11 0.03000000 sec
TD0 1

======== CHANNEL f1 ========
SFO1 100.6278588 MHz
NUC1 13C
P1 8.90 usec
PLW1 54.00000000 W

======== CHANNEL f2 ========
SFO2 400.1516006 MHz
NUC2 1H
CPDPRG[2 waltz16
PCPD2 90.00 usec
PLW2 12.00000000 W
PLW12 0.32231000 W

F2 - Processing parameters
SI 16384
SF 100.6177834 MHz
WDW EM
SSB 0
LB 1.00 Hz
GB 0
PC 0.80

1H of VBSJ-390

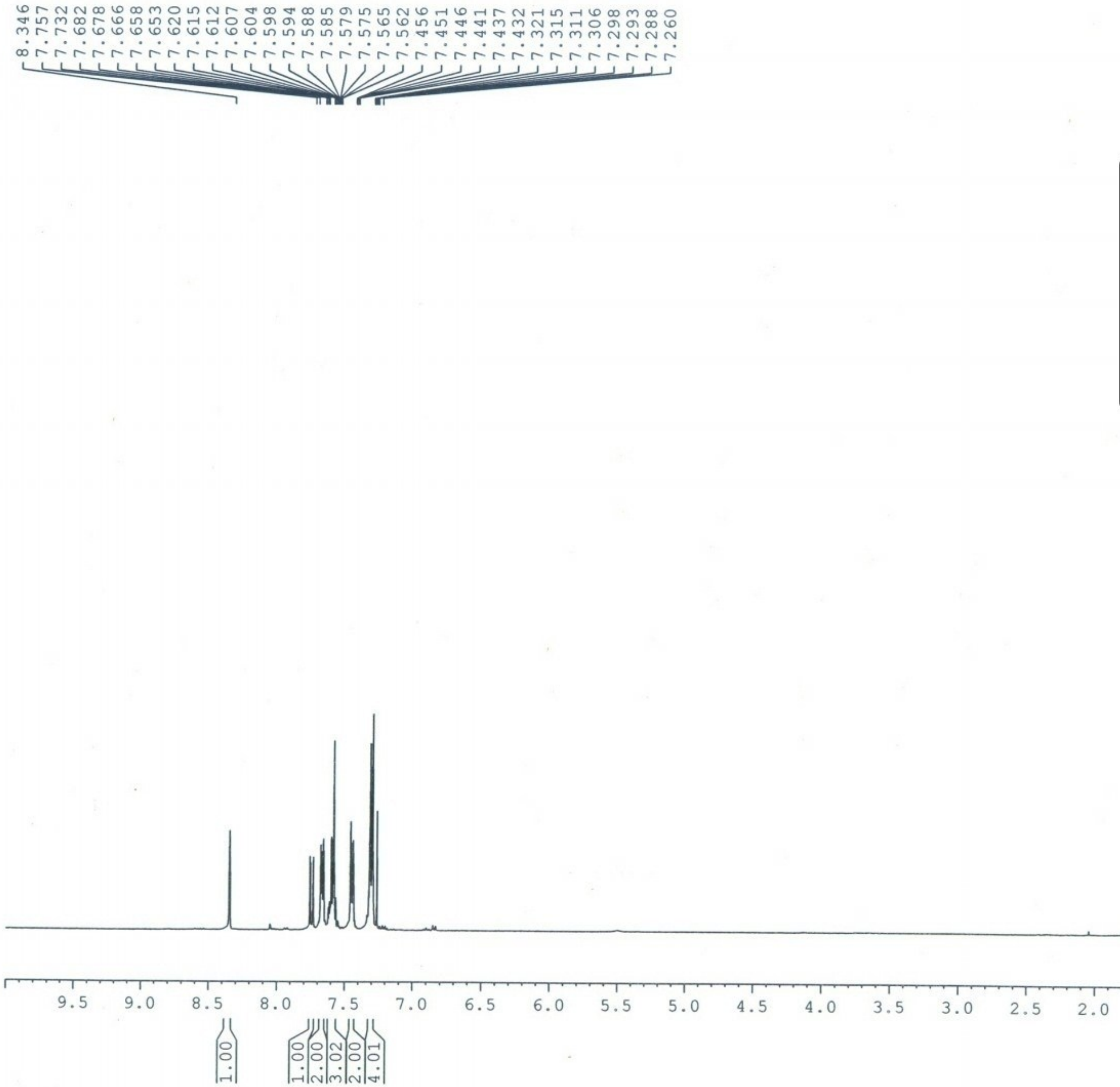

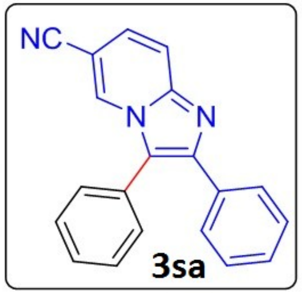

3sa

BRUKER

Current Data Parameters
NAME Dr.A.HAJRA 2017
EXPNO 1636
PROCNO 1

F2 - Acquisition Parameters
Date_ 20171008
Time 10.15
INSTRUM spect
PROBHD 5 mm PABBO BB/
PULPROG zg30
TD 32768
SOLVENT CDCl3
NS 20
DS 1
SWH 8223.685 Hz
FIDRES 0.250967 Hz
AQ 1.9922944 sec
RG 106.66
DW 60.800 usec
DE 6.50 usec
TE 296.0 K
D1 1.00000000 sec
TD0 1

======== CHANNEL f1 ========
SFO1 400.1524711 MHz
NUC1 1H
P1 14.75 usec
PLW1 12.00000000 W

F2 - Processing parameters
SI 16384
SF 400.1500095 MHz
WDW EM
SSB 0
LB 0.30 Hz
GB 0
PC 1.00

13C of VBSJ-390

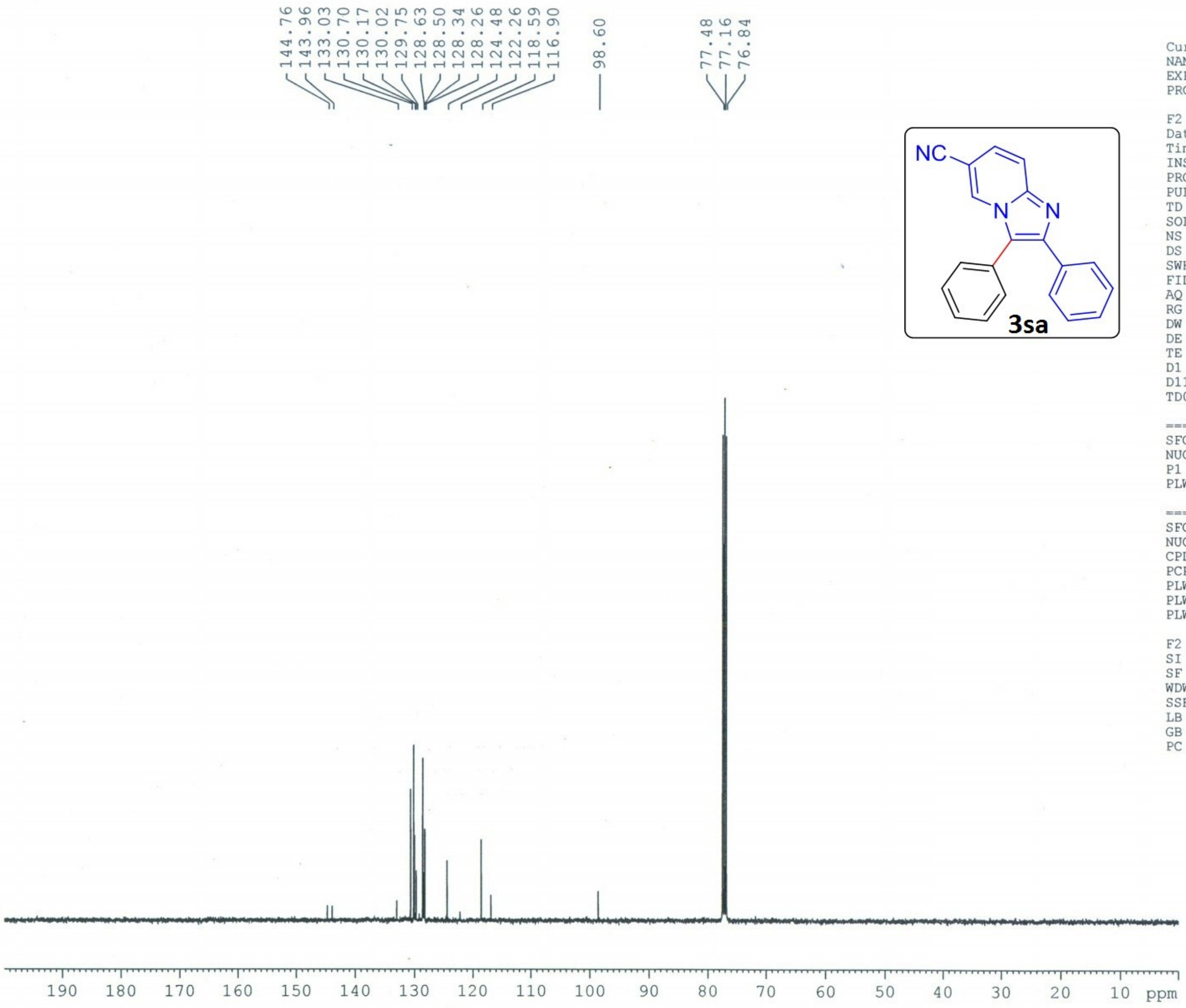

3sa

```
Current Data Parameters
NAME      Dr.A.HAJRA 2017
EXPNO                1637
PROCNO                  1

F2 - Acquisition Parameters
Date_            20171008
Time                11.04
INSTRUM             spect
PROBHD   5 mm PABBO BB/
PULPROG            zgpg30
TD                  32768
SOLVENT             CDCl3
NS                   1024
DS                      2
SWH            24038.461 Hz
FIDRES          0.733596 Hz
AQ             0.6815744 sec
RG                 106.66
DW                 20.800 usec
DE                   6.50 usec
TE                  296.1 K
D1             2.00000000 sec
D11            0.03000000 sec
TD0                     1

======== CHANNEL f1 ========
SFO1        100.6278588 MHz
NUC1                  13C
P1                   8.90 usec
PLW1          54.00000000 W

======== CHANNEL f2 ========
SFO2        400.1516006 MHz
NUC2                   1H
CPDPRG[2          waltz16
PCPD2               90.00 usec
PLW2          12.00000000 W
PLW12          0.32231000 W
PLW13          0.16212000 W

F2 - Processing parameters
SI                  16384
SF          100.6177851 MHz
WDW                    EM
SSB      0
LB                   1.00 Hz
GB       0
PC                   1.40
```

1H of VBSJ-397 2nd

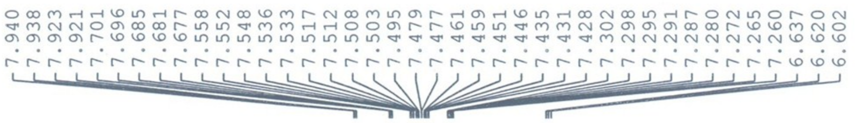
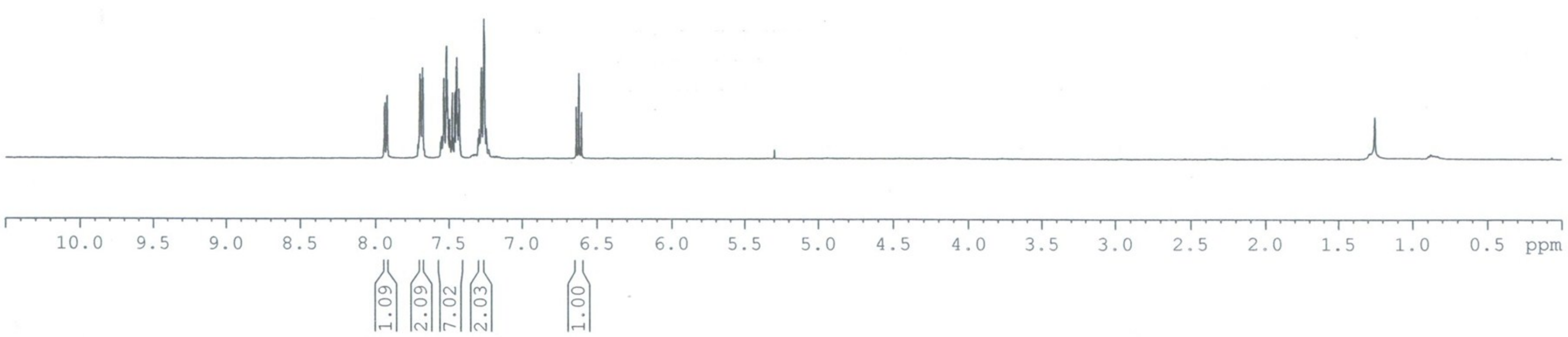

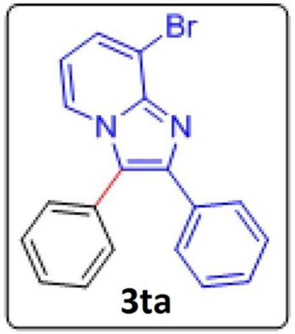

3ta

```
Current Data Parameters
NAME        Dr.A.HAJRA 2018
EXPNO                   303
PROCNO                    1

F2 - Acquisition Parameters
Date_              20180215
Time                  11.24
INSTRUM               spect
PROBHD    5 mm PABBO BB/
PULPROG                zg30
TD                    32768
SOLVENT               CDCl3
NS                       16
DS                        1
SWH                8223.685 Hz
FIDRES             0.250967 Hz
AQ                1.9922944 sec
RG                   186.42
DW                   60.800 usec
DE                     6.50 usec
TE                    296.0 K
D1               1.00000000 sec
TD0                       1

======== CHANNEL f1 ========
SFO1            400.1524711 MHz
NUC1                     1H
P1                    14.75 usec
PLW1            12.00000000 W

F2 - Processing parameters
SI                    16384
SF              400.1500000 MHz
WDW                      EM
SSB       0
LB                     0.30 Hz
GB        0
PC                     1.00
```

13C of VBSJ- 397

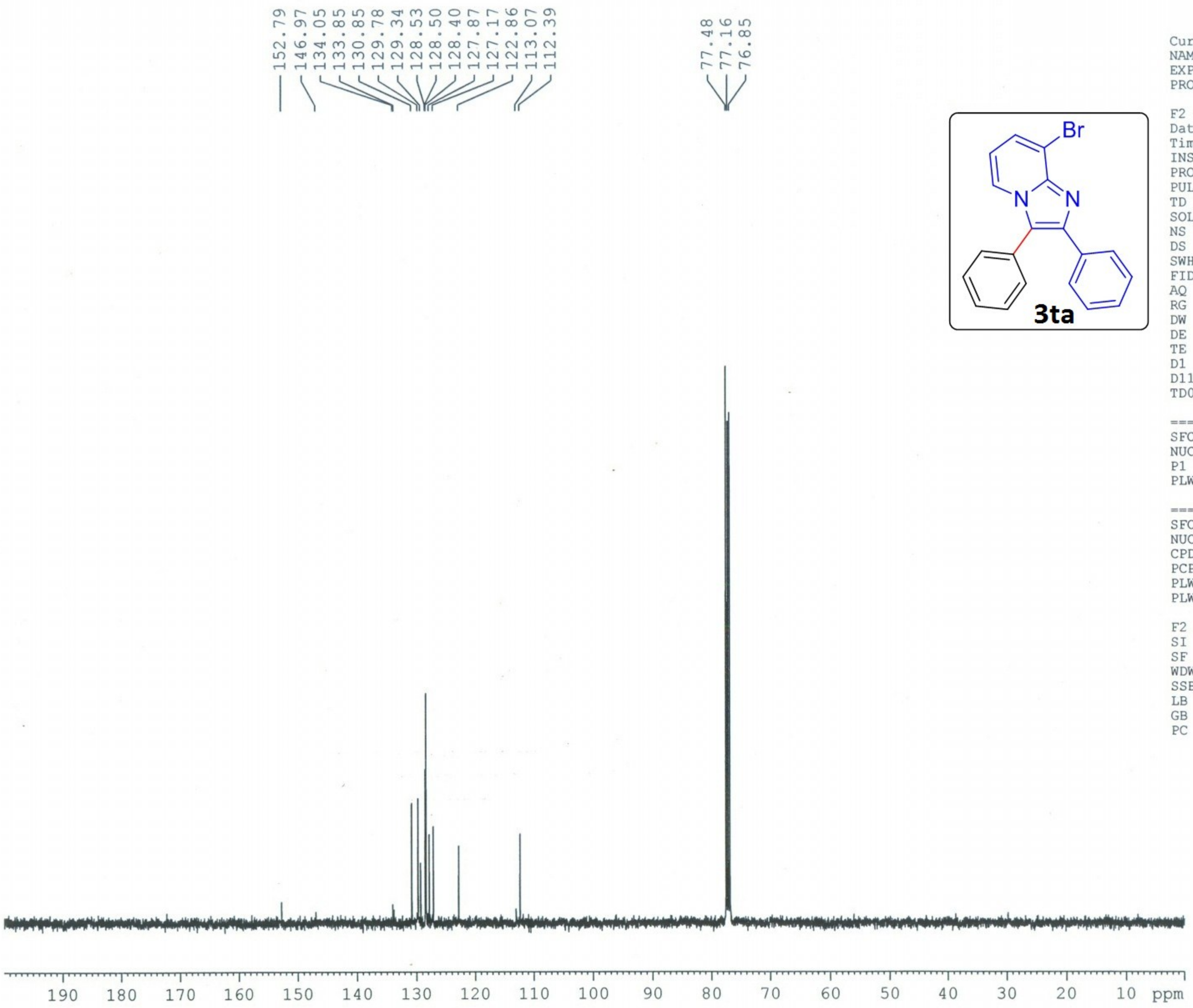

3ta

BRUKER

Current Data Parameters
NAME Dr.A.HAJRA 2017
EXPNO 1661
PROCNO 1

F2 - Acquisition Parameters
Date_ 20171012
Time 14.52
INSTRUM spect
PROBHD 5 mm PABBO BB/
PULPROG zgdc
TD 32768
SOLVENT CDCl3
NS 2048
DS 2
SWH 24038.461 Hz
FIDRES 0.733596 Hz
AQ 0.6815744 sec
RG 148.91
DW 20.800 usec
DE 6.50 usec
TE 298.7 K
D1 2.00000000 sec
D11 0.03000000 sec
TD0 1

======== CHANNEL f1 ========
SFO1 100.6278588 MHz
NUC1 13C
P1 8.90 usec
PLW1 54.00000000 W

======== CHANNEL f2 ========
SFO2 400.1516006 MHz
NUC2 1H
CPDPRG[2 waltz16
PCPD2 90.00 usec
PLW2 12.00000000 W
PLW12 0.32231000 W

F2 - Processing parameters
SI 16384
SF 100.6177825 MHz
WDW EM
SSB 0
LB 1.00 Hz
GB 0
PC 1.00

1H of VBSJ-372 2nd

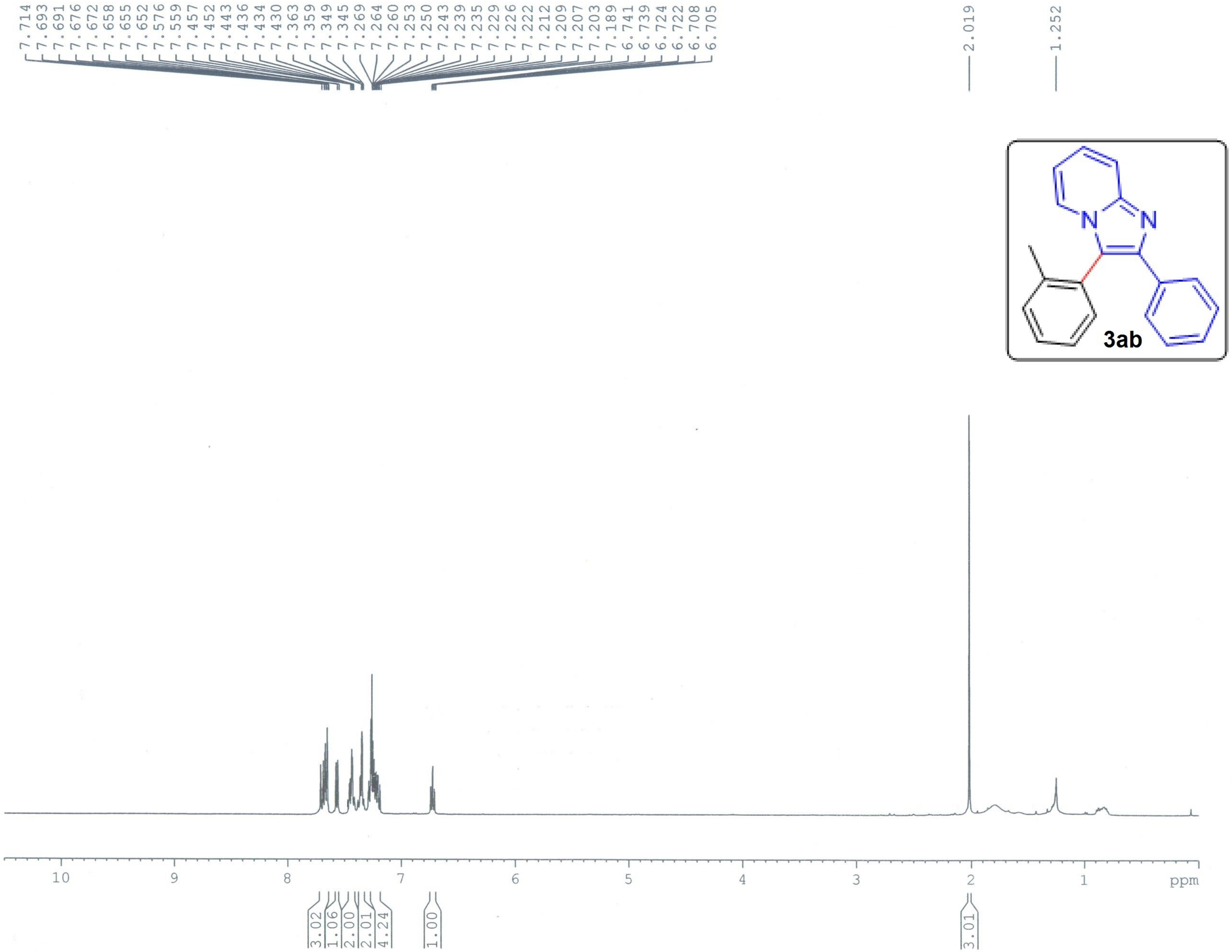

3ab

Current Data Parameters
NAME Dr.A.HAJRA 2018
EXPNO 301
PROCNO 1

F2 - Acquisition Parameters
Date_ 20180214
Time 20.32
INSTRUM spect
PROBHD 5 mm PABBO BB/
PULPROG zg30
TD 32768
SOLVENT CDCl3
NS 16
DS 1
SWH 8223.685 Hz
FIDRES 0.250967 Hz
AQ 1.9922944 sec
RG 186.42
DW 60.800 usec
DE 6.50 usec
TE 296.0 K
D1 1.00000000 sec
TD0 1

======== CHANNEL f1 ========
SFO1 400.1524711 MHz
NUC1 1H
P1 14.75 usec
PLW1 12.00000000 W

F2 - Processing parameters
SI 16384
SF 400.1500096 MHz
WDW EM
SSB 0
LB 0.30 Hz
GB 0
PC 1.00

13C of VBSJ-372

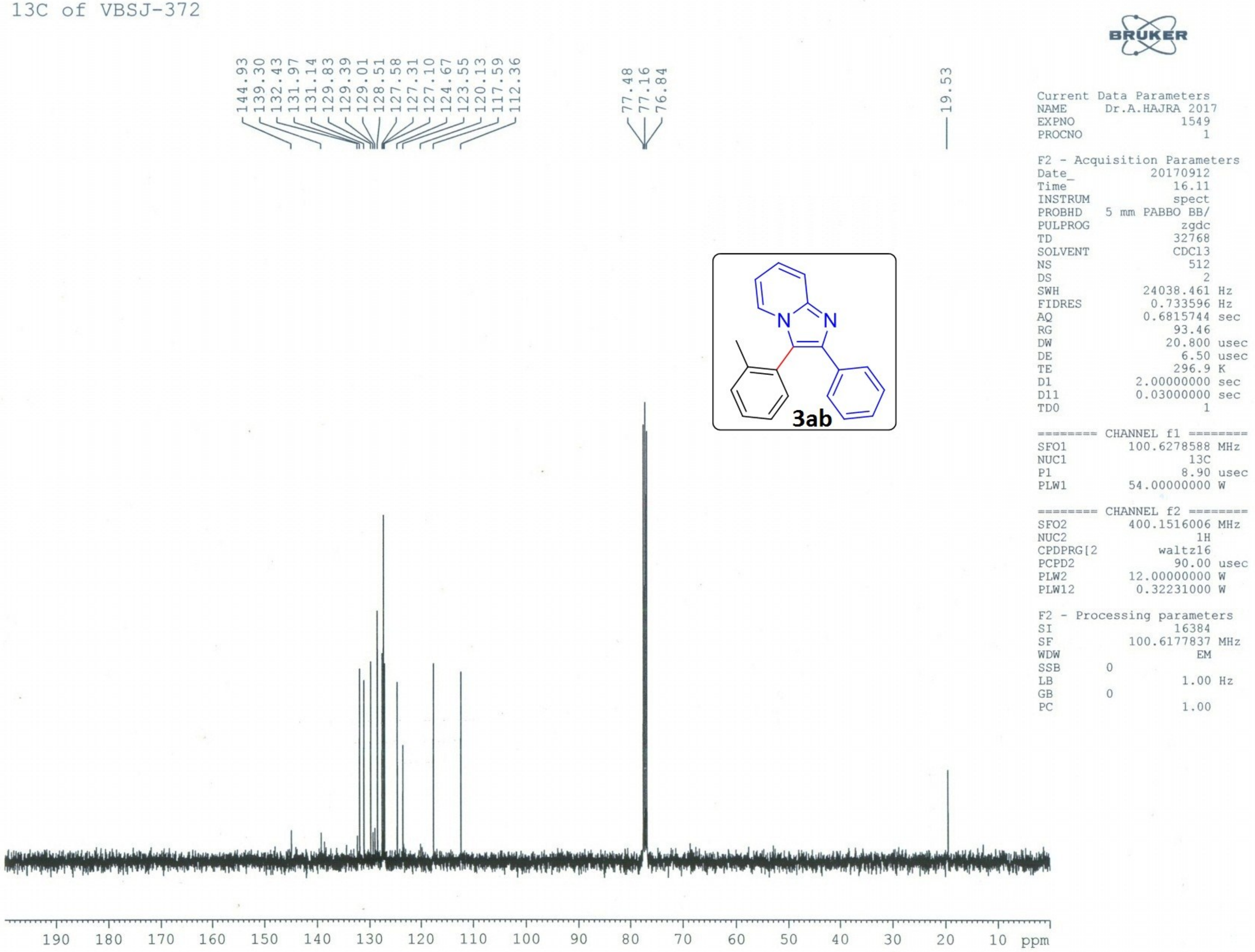

1H of VBSJ-375

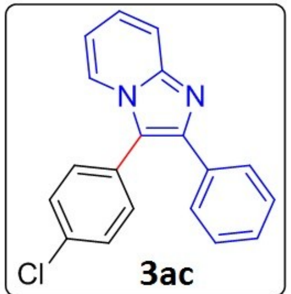

3ac

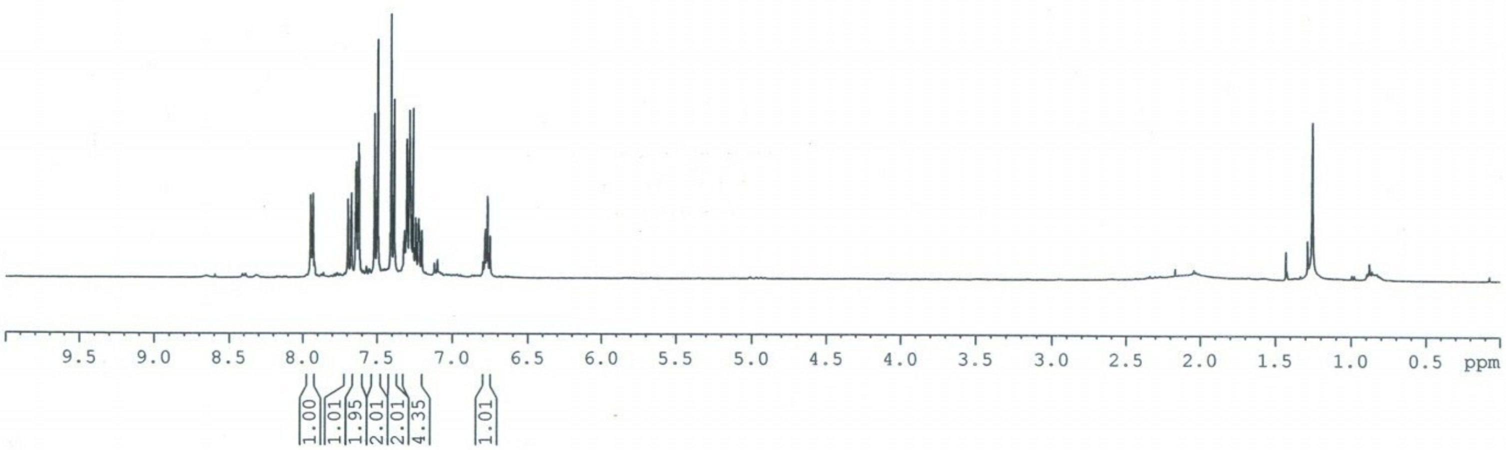

Current Data Parameters
NAME Dr.A.HAJRA 2017
EXPNO 1556
PROCNO 1

F2 - Acquisition Parameters
Date_ 20170912
Time_ 20.46
INSTRUM spect
PROBHD 5 mm PABBO BB/
PULPROG zg30
TD 32768
SOLVENT CDCl3
NS 8
DS 1
SWH 8223.685 Hz
FIDRES 0.250967 Hz
AQ 1.9922944 sec
RG 135.7
DW 60.800 usec
DE 6.50 usec
TE 298.5 K
D1 1.00000000 sec
TD0 1

======== CHANNEL f1 ========
SFO1 400.1524711 MHz
NUC1 1H
P1 14.75 usec
PLW1 12.00000000 W

F2 - Processing parameters
SI 16384
SF 400.1500095 MHz
WDW EM
SSB 0
LB 0.30 Hz
GB 0
PC 1.00

13C of VBSJ-375

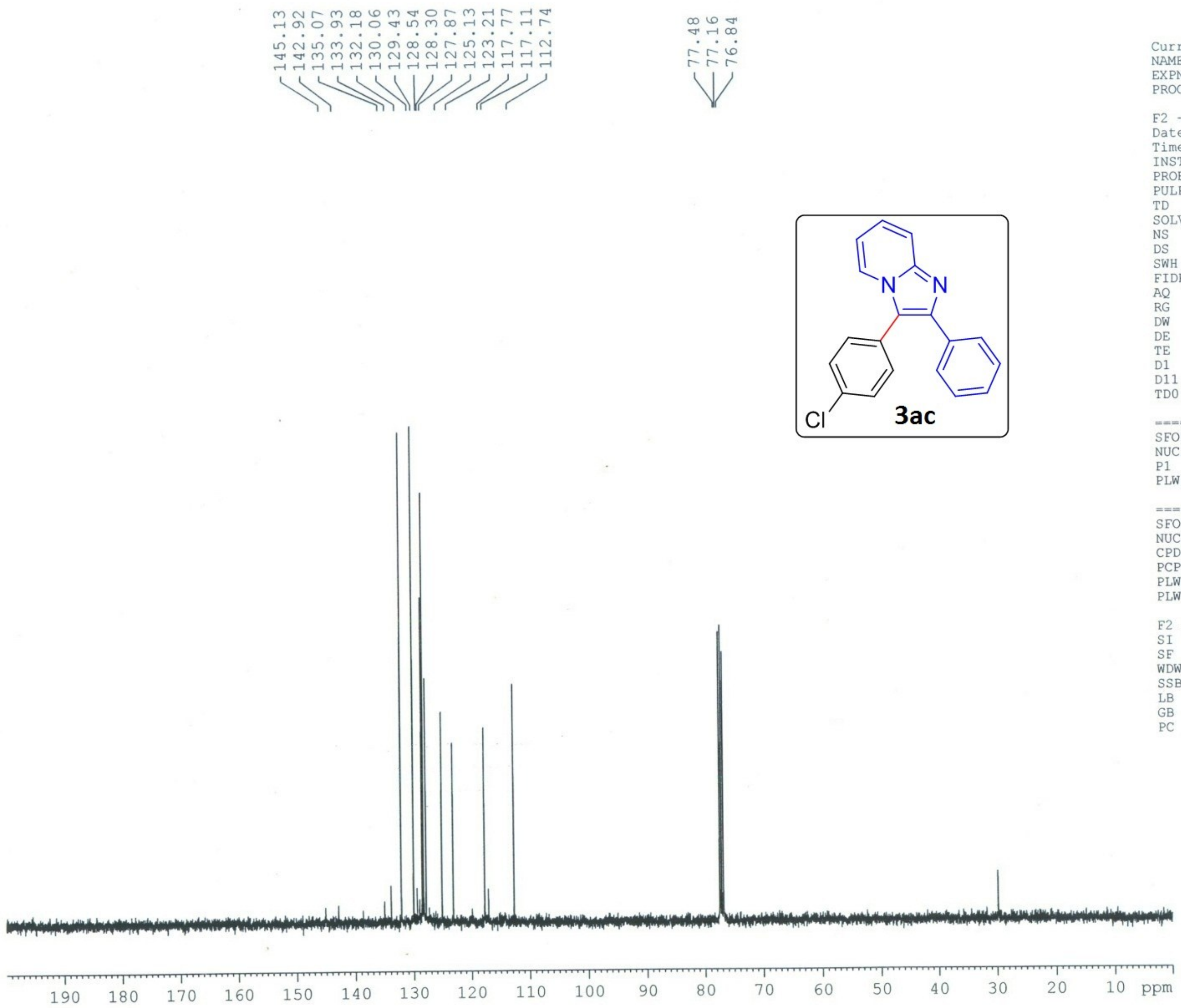

3ac

BRUKER

Current Data Parameters
NAME Dr.A.HAJRA 2017
EXPNO 1557
PROCNO 1

F2 - Acquisition Parameters
Date_ 20170912
Time 21.02
INSTRUM spect
PROBHD 5 mm PABBO BB/
PULPROG zgdc
TD 32768
SOLVENT CDCl3
NS 480
DS 2
SWH 24038.461 Hz
FIDRES 0.733596 Hz
AQ 0.6815744 sec
RG 135.7
DW 20.800 usec
DE 6.50 usec
TE 299.4 K
D1 2.00000000 sec
D11 0.03000000 sec
TD0 1

======== CHANNEL f1 ========
SFO1 100.6278588 MHz
NUC1 13C
P1 8.90 usec
PLW1 54.00000000 W

======== CHANNEL f2 ========
SFO2 400.1516006 MHz
NUC2 1H
CPDPRG[2 waltz16
PCPD2 90.00 usec
PLW2 12.00000000 W
PLW12 0.32231000 W

F2 - Processing parameters
SI 16384
SF 100.6177837 MHz
WDW EM
SSB 0
LB 1.00 Hz
GB 0
PC 1.40

1H of of VBSJ-376

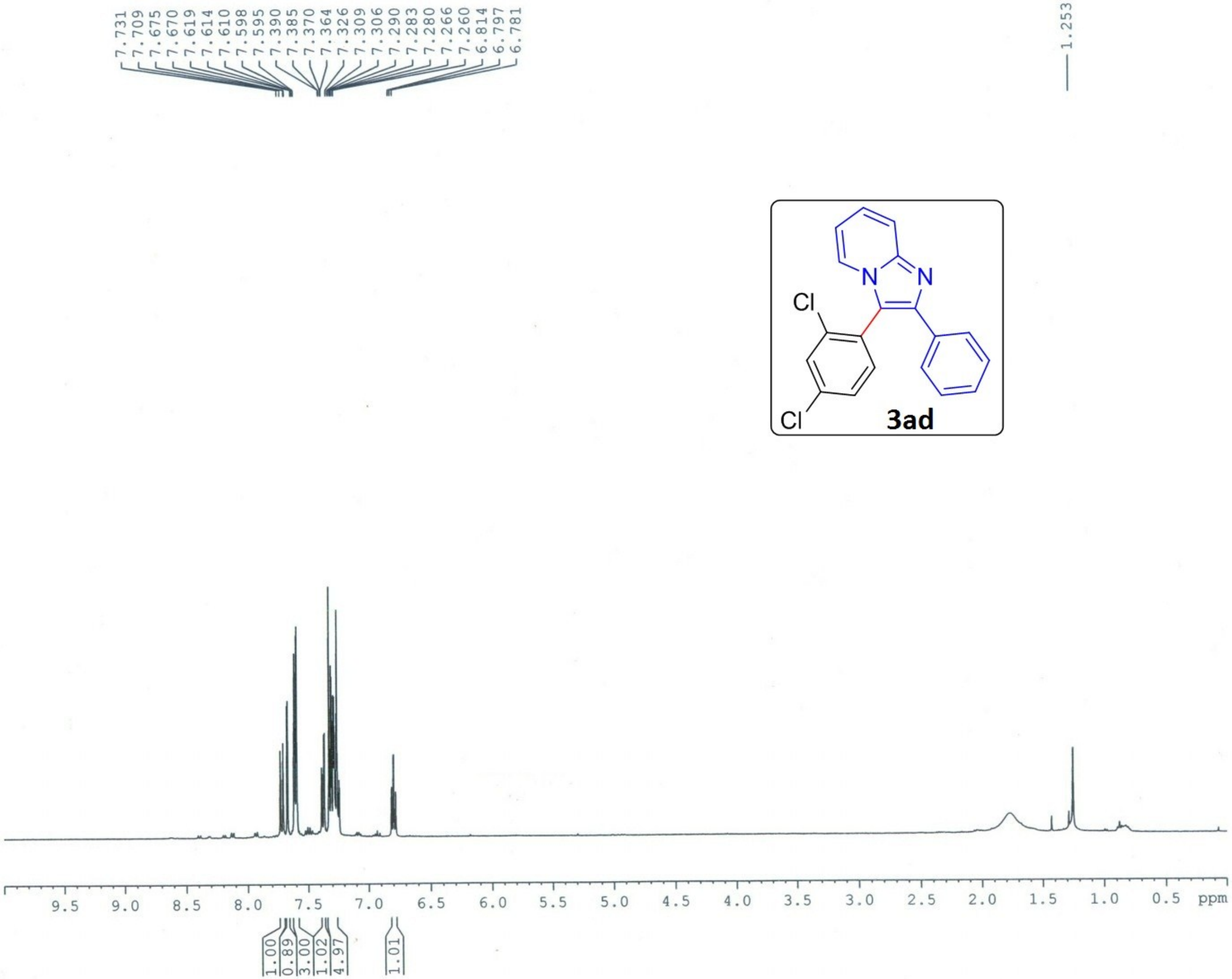

BRUKER

Current Data Parameters
NAME Dr.A.HAJRA 2017
EXPNO 1572
PROCNO 1

F2 - Acquisition Parameters
Date_ 20170916
Time 17.43
INSTRUM spect
PROBHD 5 mm PABBO BB/
PULPROG zg30
TD 32768
SOLVENT CDCl3
NS 16
DS 1
SWH 8223.685 Hz
FIDRES 0.250967 Hz
AQ 1.9922944 sec
RG 168.31
DW 60.800 usec
DE 6.50 usec
TE 298.0 K
D1 1.00000000 sec
TD0 1

======== CHANNEL f1 ========
SFO1 400.1524711 MHz
NUC1 1H
P1 14.75 usec
PLW1 12.00000000 W

F2 - Processing parameters
SI 16384
SF 400.1500096 MHz
WDW EM
SSB 0
LB 0.30 Hz
GB 0
PC 1.00

13C of VBSJ-376

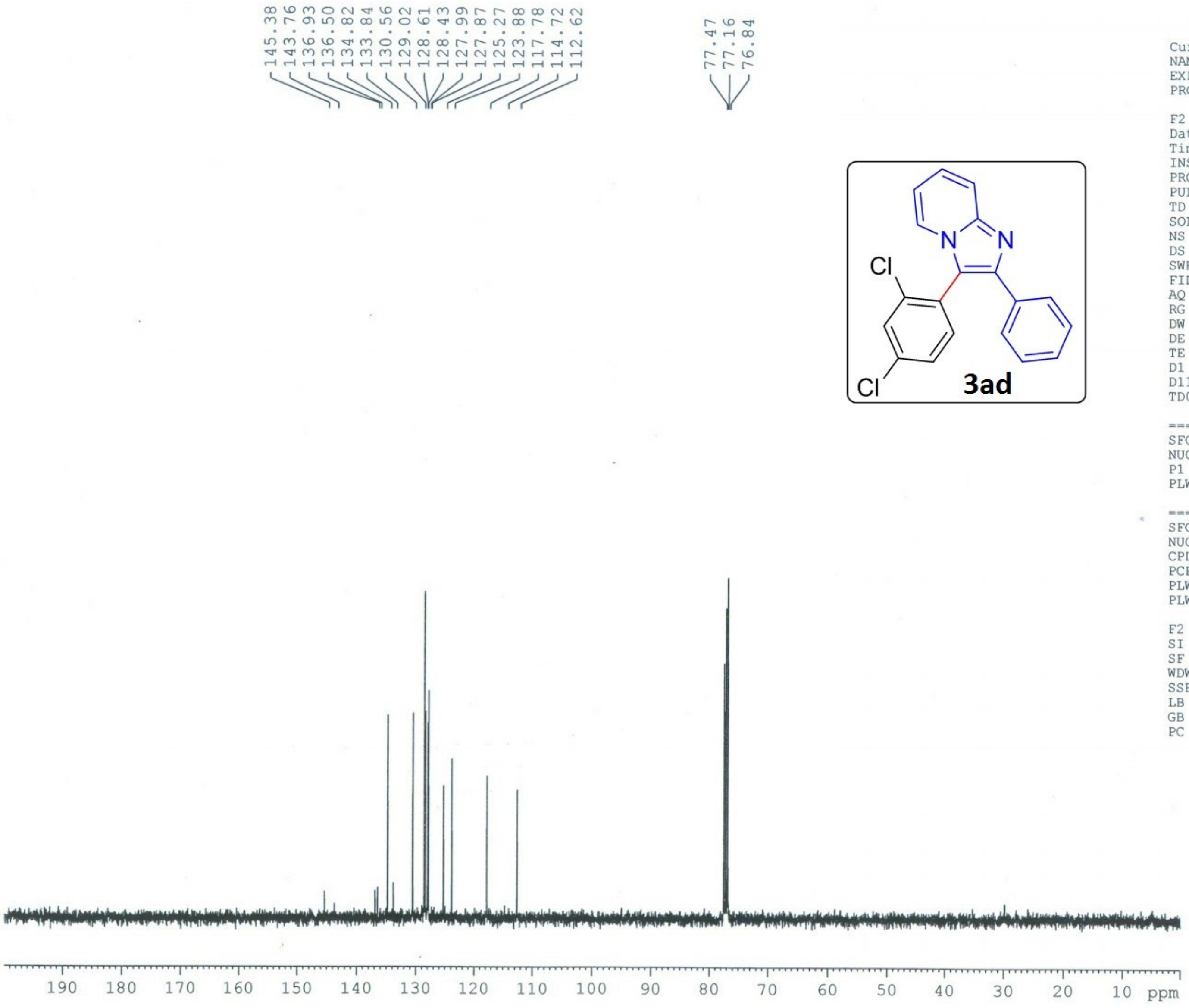

BRUKER

Current Data Parameters
NAME Dr.A.HAJRA 2017
EXPNO 1573
PROCNO 1

F2 - Acquisition Parameters
Date_ 20170916
Time 17.53
INSTRUM spect
PROBHD 5 mm PABBO BB/
PULPROG zgdc
TD 32768
SOLVENT CDCl3
NS 512
DS 2
SWH 24038.461 Hz
FIDRES 0.733596 Hz
AQ 0.6815744 sec
RG 168.31
DW 20.800 usec
DE 6.50 usec
TE 298.8 K
D1 2.00000000 sec
D11 0.03000000 sec
TD0 1

======== CHANNEL f1 ========
SFO1 100.6278588 MHz
NUC1 13C
P1 8.90 usec
PLW1 54.00000000 W

======== CHANNEL f2 ========
SFO2 400.1516006 MHz
NUC2 1H
CPDPRG[2 waltz16
PCPD2 90.00 usec
PLW2 12.00000000 W
PLW12 0.32231000 W

F2 - Processing parameters
SI 16384
SF 100.6177834 MHz
WDW EM
SSB 0
LB 1.00 Hz
GB 0
PC 1.00

1H of VBSJ-399

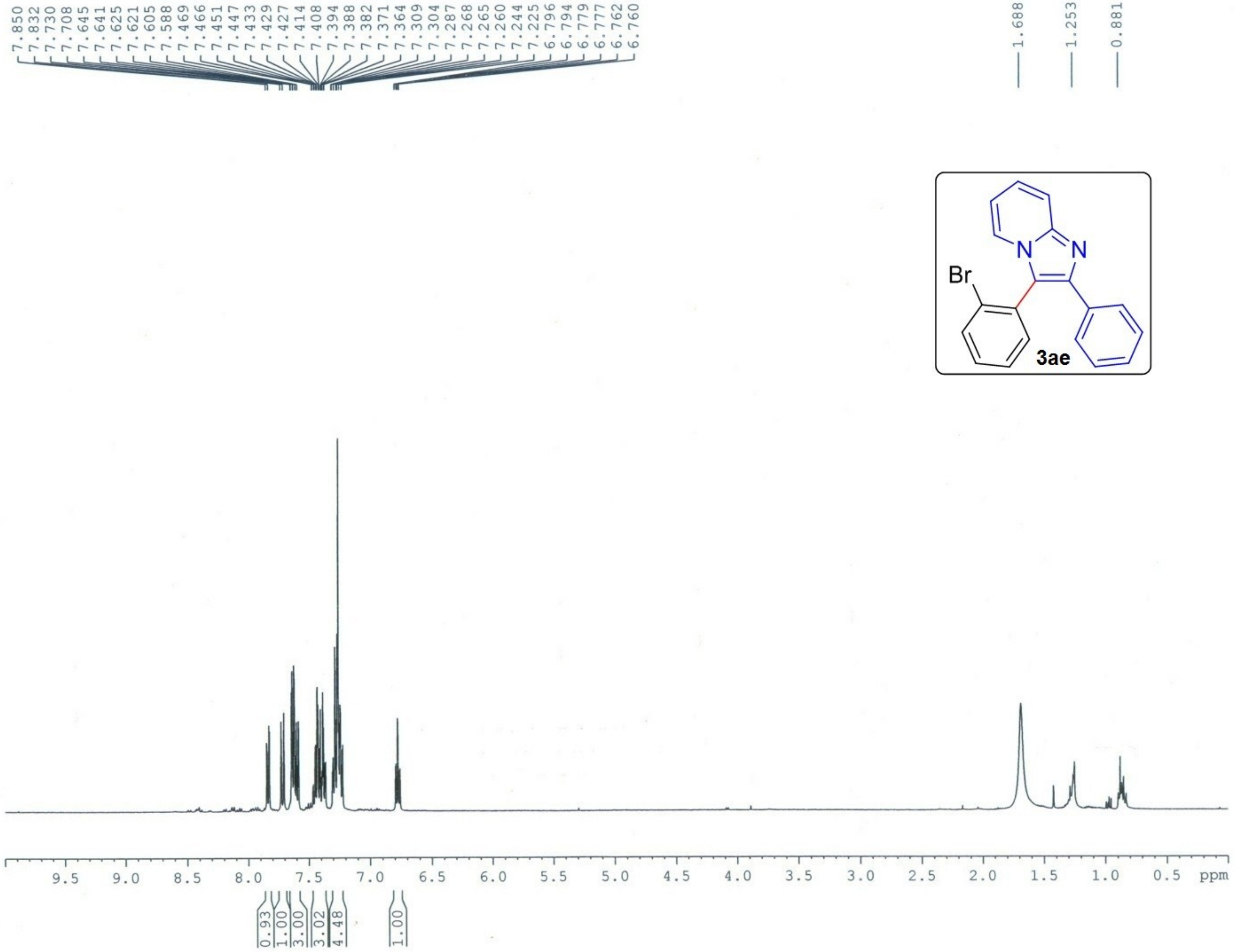

BRUKER

Current Data Parameters
NAME Dr.A.HAJRA 2017
EXPNO 1667
PROCNO 1

F2 - Acquisition Parameters
Date_ 20171013
Time 18.01
INSTRUM spect
PROBHD 5 mm PABBO BB/
PULPROG zg30
TD 32768
SOLVENT CDCl3
NS 32
DS 1
SWH 8223.685 Hz
FIDRES 0.250967 Hz
AQ 1.9922944 sec
RG 186.42
DW 60.800 usec
DE 6.50 usec
TE 297.3 K
D1 1.00000000 sec
TD0 1

======== CHANNEL f1 ========
SFO1 400.1524711 MHz
NUC1 1H
P1 14.75 usec
PLW1 12.00000000 W

F2 - Processing parameters
SI 16384
SF 400.1500095 MHz
WDW EM
SSB 0
LB 0.30 Hz
GB 0
PC 1.00

13C of VBSJ-399

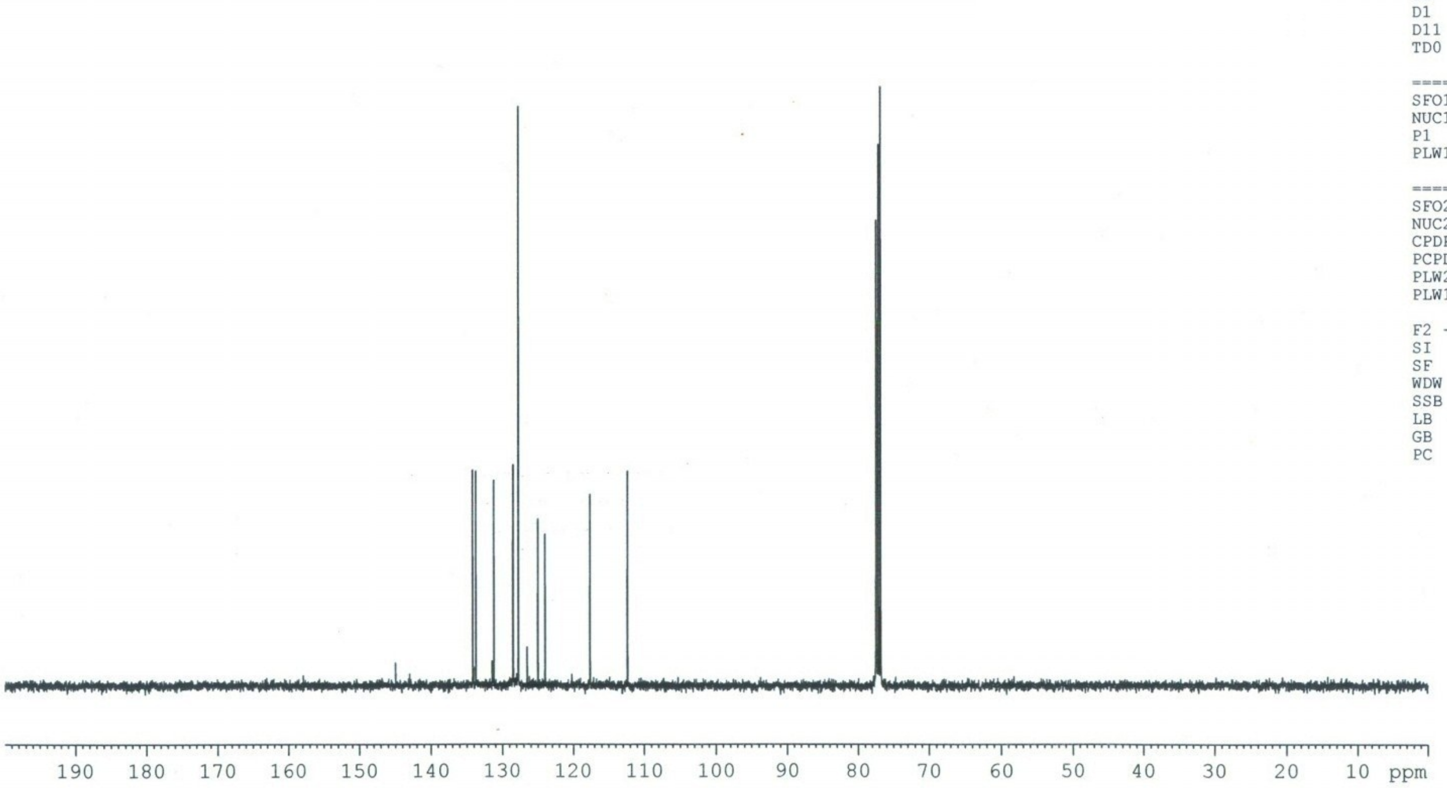

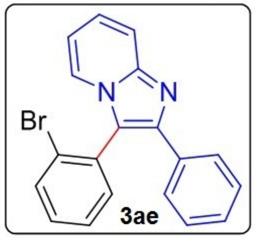

Current Data Parameters
NAME Dr.A.HAJRA 2017
EXPNO 1675
PROCNO 1

F2 - Acquisition Parameters
Date_ 20171013
Time 23.34
INSTRUM spect
PROBHD 5 mm PABBO BB/
PULPROG zgdc
TD 32768
SOLVENT CDCl3
NS 2048
DS 2
SWH 24038.461 Hz
FIDRES 0.733596 Hz
AQ 0.6815744 sec
RG 168.31
DW 20.800 usec
DE 6.50 usec
TE 298.6 K
D1 2.00000000 sec
D11 0.03000000 sec
TD0 1

======== CHANNEL f1 ========
SFO1 100.6278588 MHz
NUC1 13C
P1 8.90 usec
PLW1 54.00000000 W

======== CHANNEL f2 ========
SFO2 400.1516006 MHz
NUC2 1H
CPDPRG[2 waltz16
PCPD2 90.00 usec
PLW2 12.00000000 W
PLW12 0.32231000 W

F2 - Processing parameters
SI 16384
SF 100.6177833 MHz
WDW EM
SSB 0
LB 1.00 Hz
GB 0
PC 1.00

1H of VBSJ-360

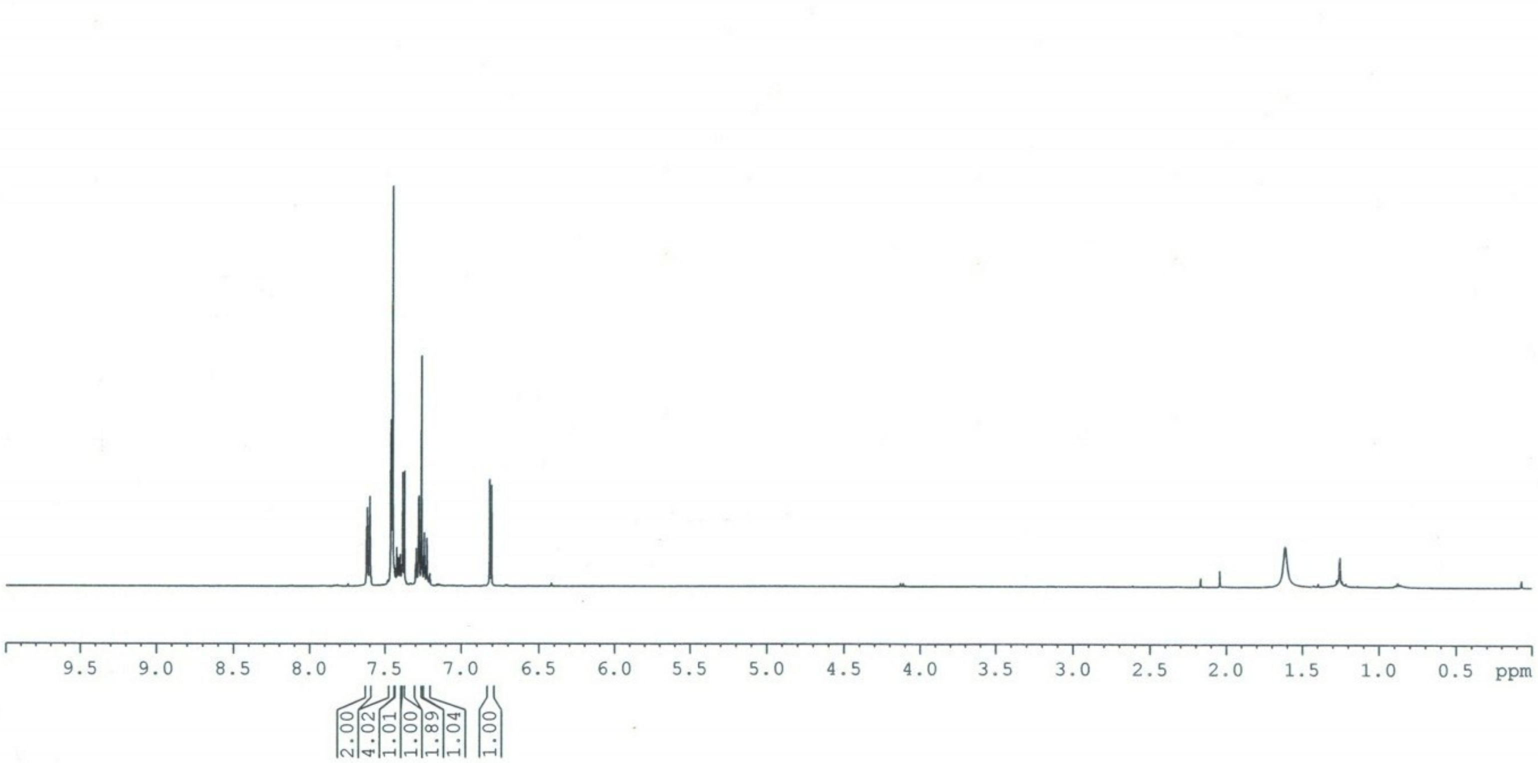

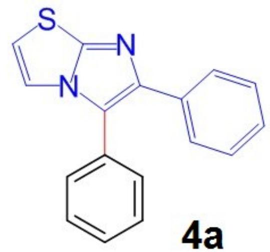

4a

BRUKER

```
Current Data Parameters
NAME        Dr.A.HAJRA 2017
EXPNO                  1471
PROCNO                    1

F2 - Acquisition Parameters
Date_              20170825
Time                  21.59
INSTRUM                spect
PROBHD   5 mm PABBO BB/
PULPROG                zg30
TD                    32768
SOLVENT               CDCl3
NS                       32
DS                        1
SWH                8223.685 Hz
FIDRES             0.250967 Hz
AQ                1.9922944 sec
RG                   186.42
DW                   60.800 usec
DE                     6.50 usec
TE                    298.3 K
D1               1.00000000 sec
TD0                       1

======== CHANNEL f1 ========
SFO1            400.1524711 MHz
NUC1                     1H
P1                    14.75 usec
PLW1            12.00000000 W

F2 - Processing parameters
SI                    16384
SF              400.1500097 MHz
WDW                      EM
SSB      0
LB                     0.30 Hz
GB       0
PC                     1.00
```

13C of VBSJ-360

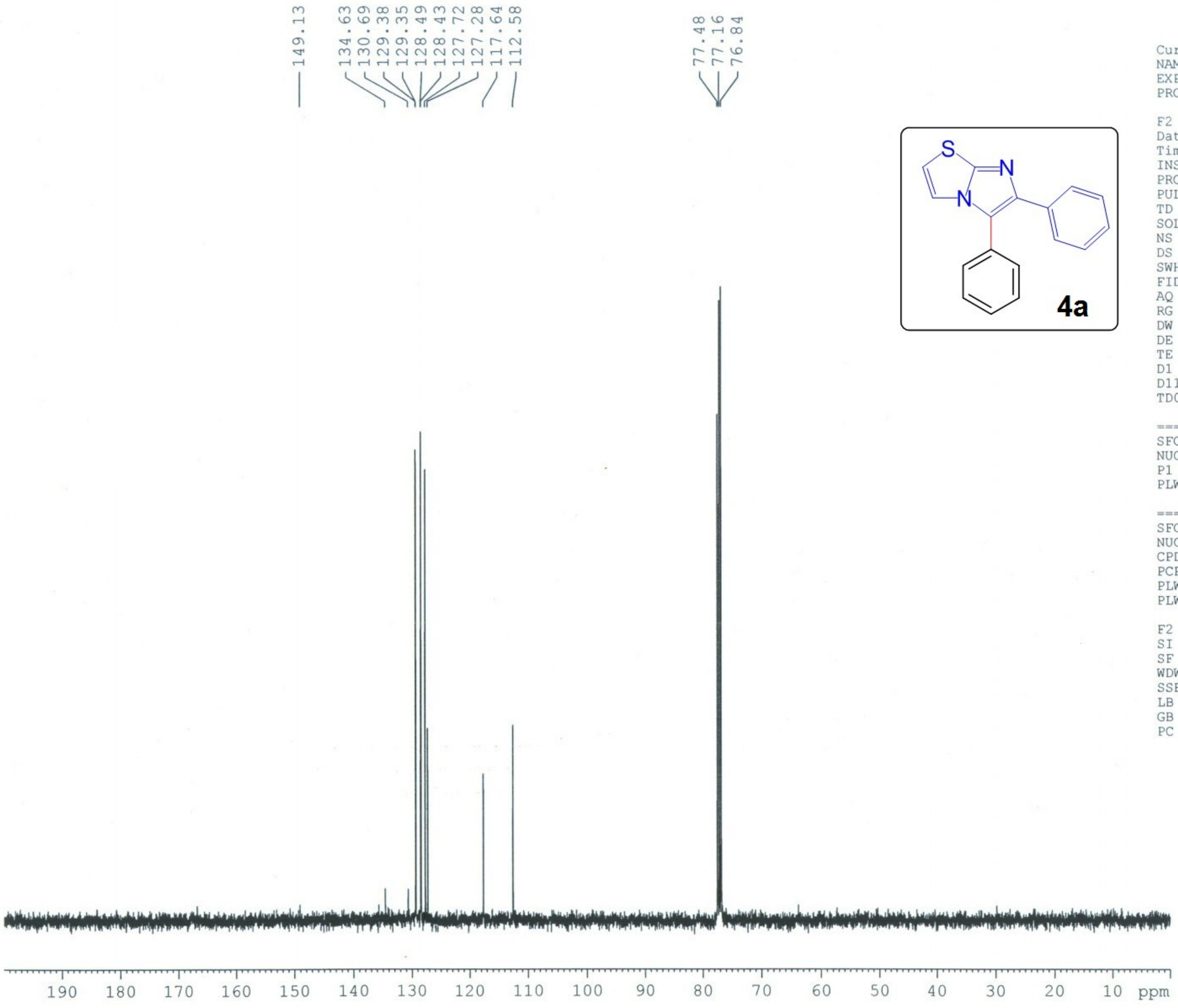

4a

```
Current Data Parameters
NAME        Dr.A.HAJRA 2017
EXPNO                  1474
PROCNO                    1

F2 - Acquisition Parameters
Date_              20170826
Time                   0.34
INSTRUM               spect
PROBHD    5 mm PABBO BB/
PULPROG                zgdc
TD                    32768
SOLVENT               CDCl3
NS                     1024
DS                        2
SWH              24038.461 Hz
FIDRES            0.733596 Hz
AQ               0.6815744 sec
RG                   186.42
DW                   20.800 usec
DE                     6.50 usec
TE                    298.2 K
D1               2.00000000 sec
D11              0.03000000 sec
TD0                       1

======== CHANNEL f1 ========
SFO1          100.6278588 MHz
NUC1                    13C
P1                     8.90 usec
PLW1         54.00000000 W

======== CHANNEL f2 ========
SFO2          400.1516006 MHz
NUC2                     1H
CPDPRG[2            waltz16
PCPD2                 90.00 usec
PLW2         12.00000000 W
PLW12         0.32231000 W

F2 - Processing parameters
SI                    16384
SF            100.6177835 MHz
WDW                      EM
SSB      0
LB                     1.00 Hz
GB       0
PC                     1.10
```

1H of VBSJ-413

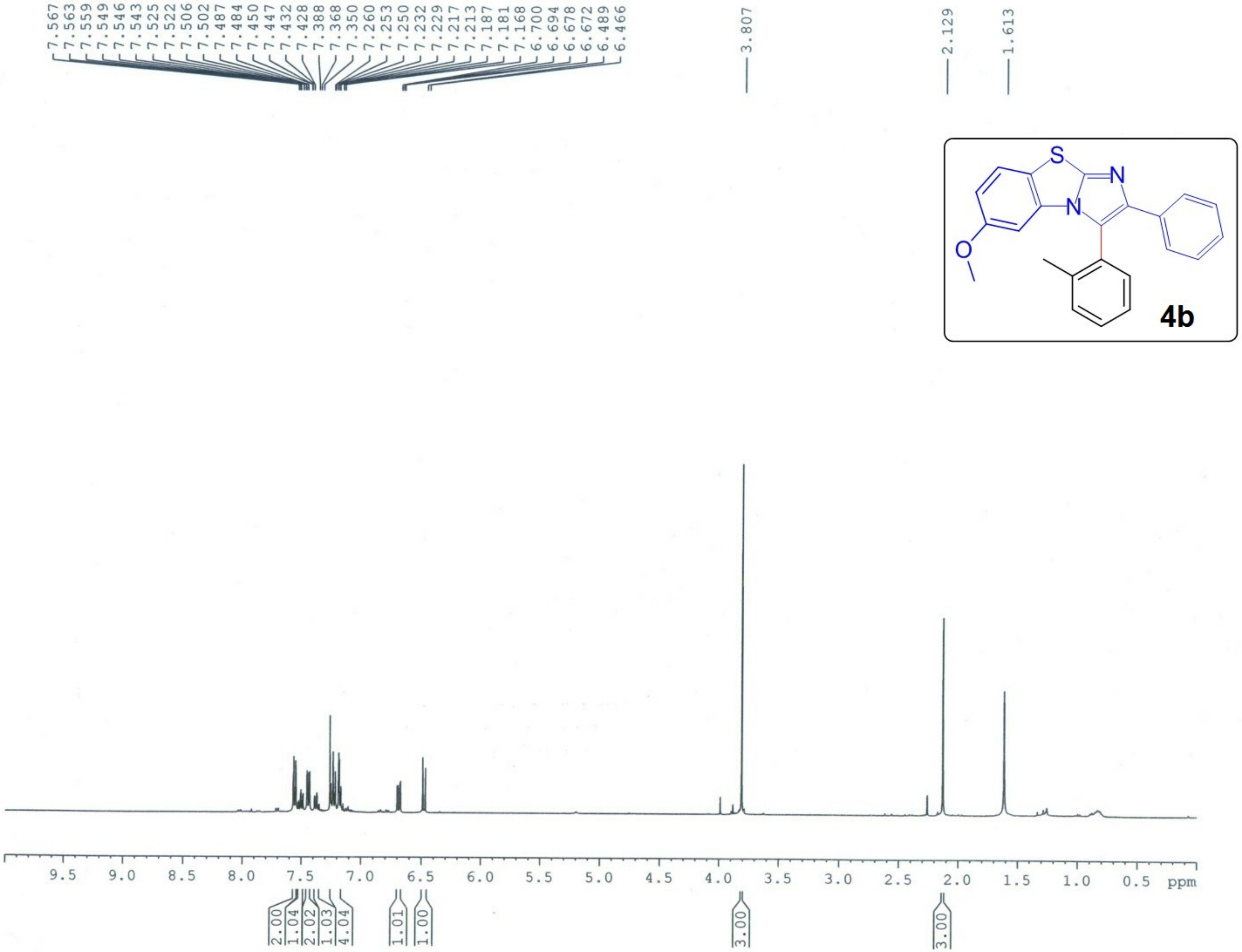

4b

Current Data Parameters
NAME Dr.A.HAJRA 2017
EXPNO 1913
PROCNO 1

F2 - Acquisition Parameters
Date_ 20171114
Time 18.11
INSTRUM spect
PROBHD 5 mm PABBO BB/
PULPROG zg30
TD 32768
SOLVENT CDCl3
NS 32
DS 1
SWH 8223.685 Hz
FIDRES 0.250967 Hz
AQ 1.9922944 sec
RG 135.7
DW 60.800 usec
DE 6.50 usec
TE 296.1 K
D1 1.00000000 sec
TD0 1

======== CHANNEL f1 ========
SFO1 400.1524711 MHz
NUC1 1H
P1 14.75 usec
PLW1 12.00000000 W

F2 - Processing parameters
SI 16384
SF 400.1500095 MHz
WDW EM
SSB 0
LB 0.30 Hz
GB 0
PC 0.20

13C of VBSJ-413

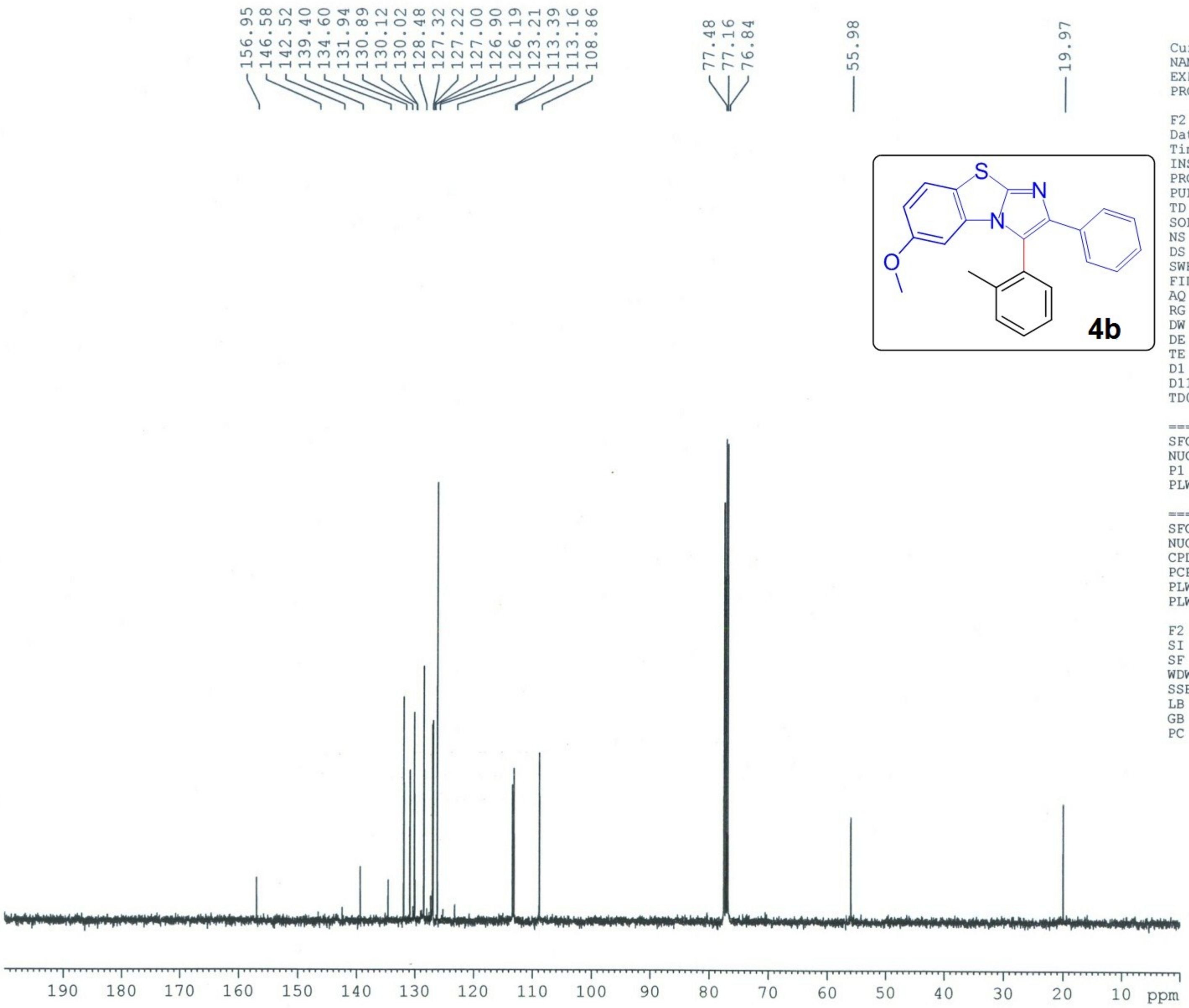

4b

BRUKER

Current Data Parameters
NAME Dr.A.HAJRA 2017
EXPNO 1915
PROCNO 1

F2 - Acquisition Parameters
Date_ 20171114
Time_ 20.54
INSTRUM spect
PROBHD 5 mm PABBO BB/
PULPROG zgdc
TD 32768
SOLVENT CDCl3
NS 2048
DS 2
SWH 24038.461 Hz
FIDRES 0.733596 Hz
AQ 0.6815744 sec
RG 135.7
DW 20.800 usec
DE 6.50 usec
TE 297.7 K
D1 2.00000000 sec
D11 0.03000000 sec
TD0 1

======== CHANNEL f1 ========
SFO1 100.6278588 MHz
NUC1 13C
P1 8.90 usec
PLW1 54.00000000 W

======== CHANNEL f2 ========
SFO2 400.1516006 MHz
NUC2 1H
CPDPRG[2 waltz16
PCPD2 90.00 usec
PLW2 12.00000000 W
PLW12 0.32231000 W

F2 - Processing parameters
SI 16384
SF 100.6177835 MHz
WDW EM
SSB 0
LB 1.00 Hz
GB 0
PC 1.00

1H of of VBSJ-408

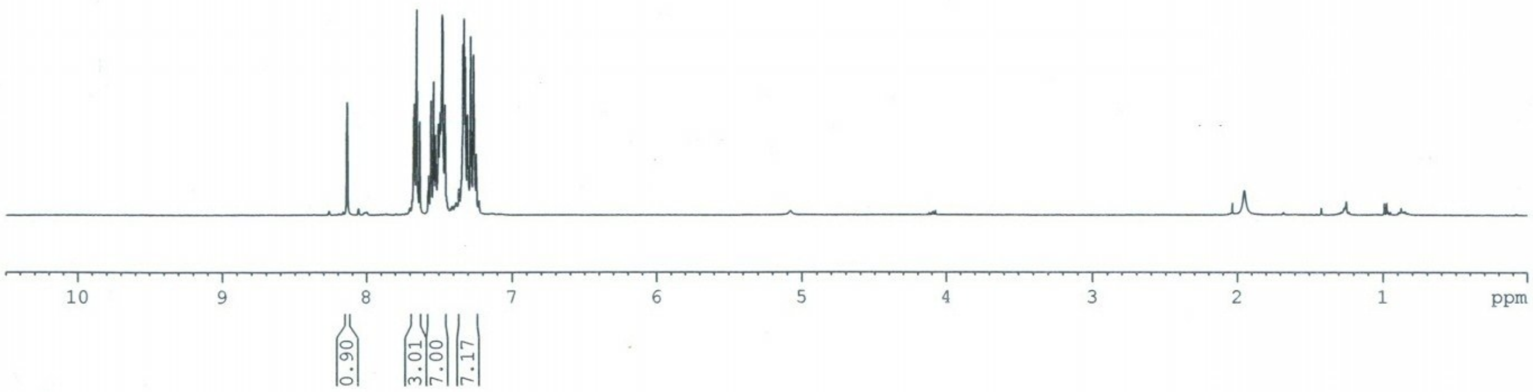

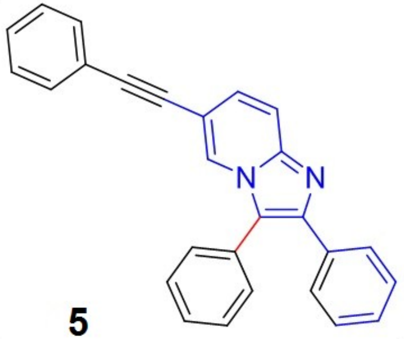

5

Current Data Parameters
NAME Dr.A.HAJRA 2017
EXPNO 1790
PROCNO 1

F2 - Acquisition Parameters
Date_ 20171102
Time 18.03
INSTRUM spect
PROBHD 5 mm PABBO BB/
PULPROG zg30
TD 32768
SOLVENT CDCl3
NS 16
DS 1
SWH 8223.685 Hz
FIDRES 0.250967 Hz
AQ 1.9922944 sec
RG 67.81
DW 60.800 usec
DE 6.50 usec
TE 296.8 K
D1 1.00000000 sec
TD0 1

======== CHANNEL f1 ========
SFO1 400.1524711 MHz
NUC1 1H
P1 14.75 usec
PLW1 12.00000000 W

F2 - Processing parameters
SI 16384
SF 400.1500149 MHz
WDW EM
SSB 0
LB 0.30 Hz
GB 0
PC 1.00

13C of VBSJ-408

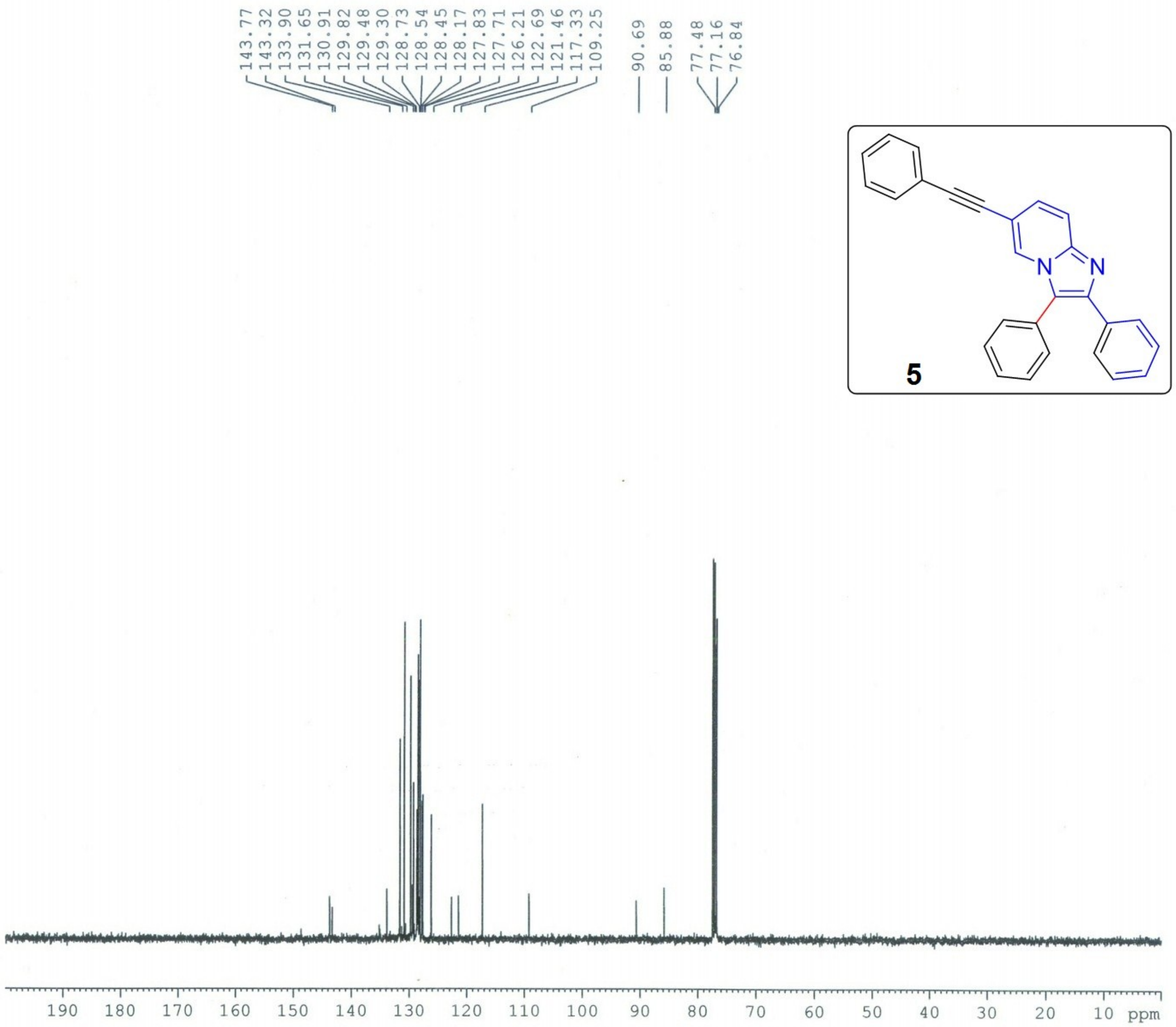

Current Data Parameters
NAME Dr.A.HAJRA 2017
EXPNO 1791
PROCNO 1

F2 - Acquisition Parameters
Date_ 20171102
Time 18.14
INSTRUM spect
PROBHD 5 mm PABBO BB/
PULPROG zgpg30
TD 32768
SOLVENT CDCl3
NS 210
DS 2
SWH 24038.461 Hz
FIDRES 0.733596 Hz
AQ 0.6815744 sec
RG 30.11
DW 20.800 usec
DE 6.50 usec
TE 297.4 K
D1 2.00000000 sec
D11 0.03000000 sec
TD0 1

======== CHANNEL f1 ========
SFO1 100.6278588 MHz
NUC1 13C
P1 8.90 usec
PLW1 54.00000000 W

======== CHANNEL f2 ========
SFO2 400.1516006 MHz
NUC2 1H
CPDPRG[2 waltz16
PCPD2 90.00 usec
PLW2 12.00000000 W
PLW12 0.32231000 W
PLW13 0.16212000 W

F2 - Processing parameters
SI 16384
SF 100.6177881 MHz
WDW EM
SSB 0
LB 1.00 Hz
GB 0
PC 1.20

1H of of VBSJ 409

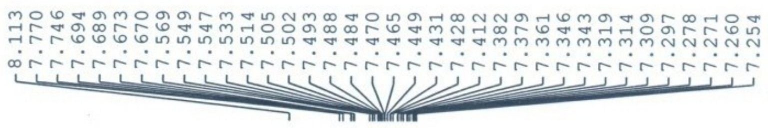

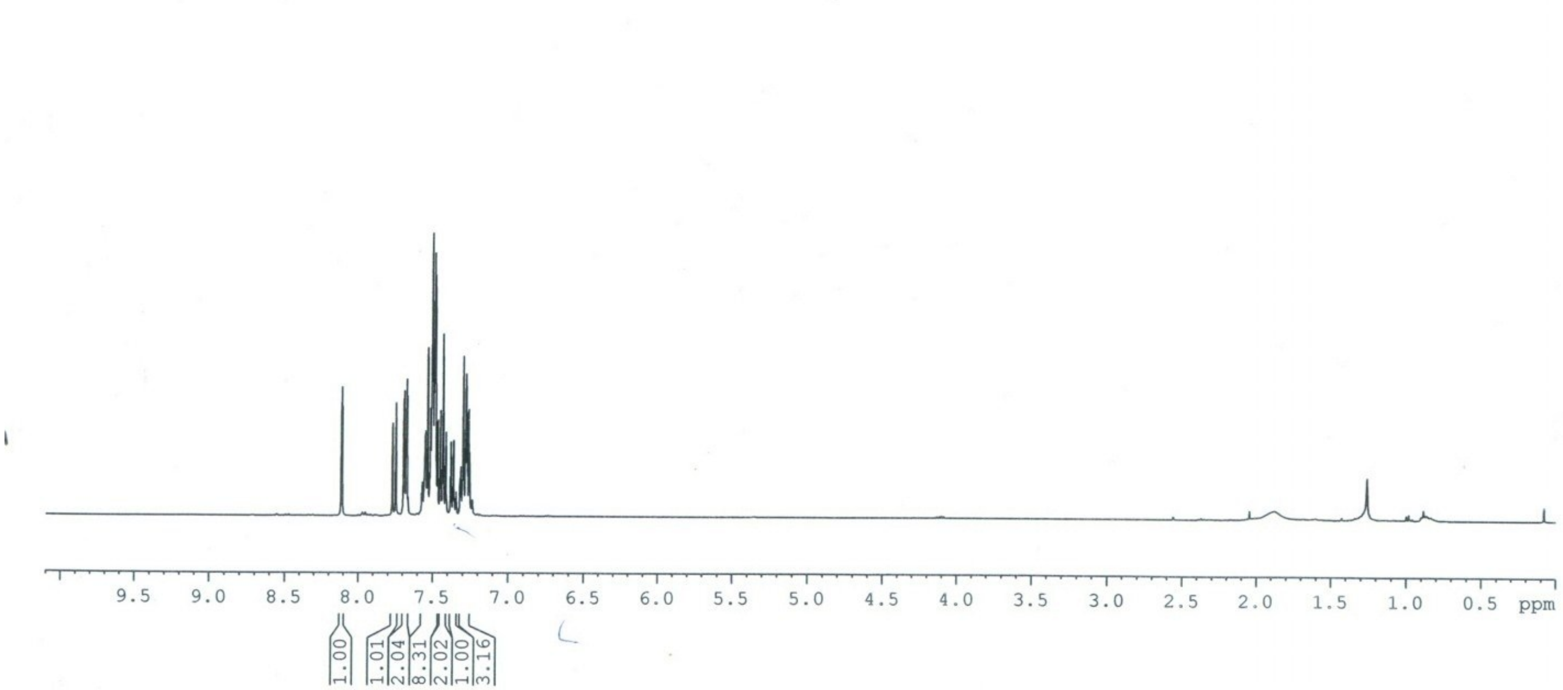

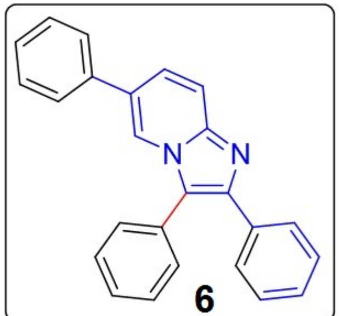

6

Current Data Parameters
NAME Dr.A.HAJRA 2017
EXPNO 1849
PROCNO 1

F2 - Acquisition Parameters
Date_ 20171107
Time 11.57
INSTRUM spect
PROBHD 5 mm PABBO BB/
PULPROG zg30
TD 32768
SOLVENT CDCl3
NS 16
DS 1
SWH 8223.685 Hz
FIDRES 0.250967 Hz
AQ 1.9922944 sec
RG 87.66
DW 60.800 usec
DE 6.50 usec
TE 295.7 K
D1 1.00000000 sec
TD0 1

======== CHANNEL f1 ========
SFO1 400.1524711 MHz
NUC1 1H
P1 14.75 usec
PLW1 12.00000000 W

F2 - Processing parameters
SI 16384
SF 400.1500096 MHz
WDW EM
SSB 0
LB 0.30 Hz
GB 0
PC 1.00

13C of VBSJ 409

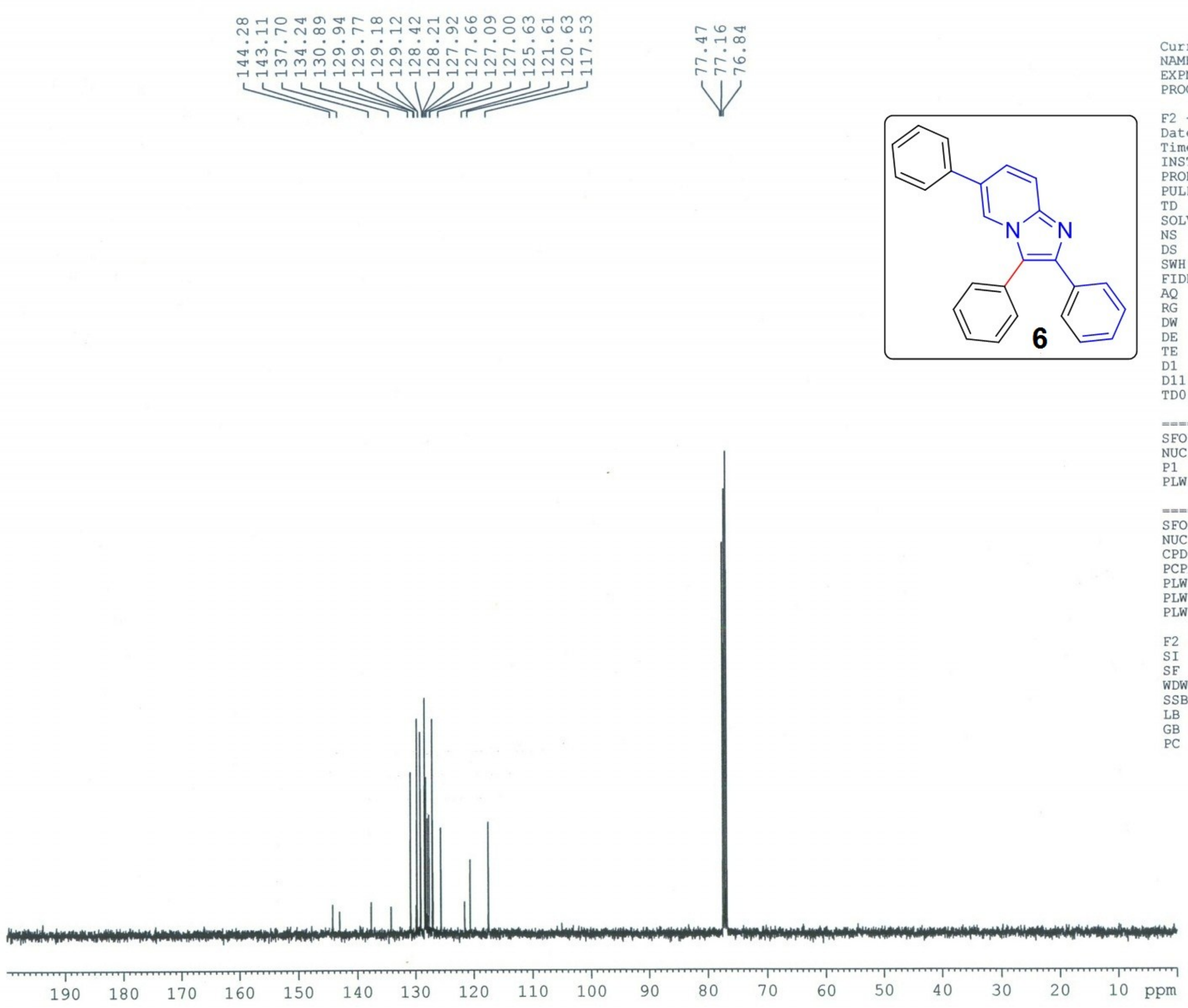


```
Current Data Parameters
NAME        Dr.A.HAJRA 2017
EXPNO                  1850
PROCNO                    1

F2 - Acquisition Parameters
Date_             20171107
Time                 12.09
INSTRUM              spect
PROBHD    5 mm PABBO BB/
PULPROG            zgpg30
TD                  32768
SOLVENT             CDCl3
NS                    180
DS                      2
SWH             24038.461 Hz
FIDRES           0.733596 Hz
AQ              0.6815744 sec
RG                  77.59
DW                 20.800 usec
DE                   6.50 usec
TE                  296.4 K
D1             2.00000000 sec
D11            0.03000000 sec
TD0                     1

======== CHANNEL f1 ========
SFO1          100.6278588 MHz
NUC1                  13C
P1                   8.90 usec
PLW1          54.00000000 W

======== CHANNEL f2 ========
SFO2          400.1516006 MHz
NUC2                   1H
CPDPRG[2          waltz16
PCPD2               90.00 usec
PLW2          12.00000000 W
PLW12          0.32231000 W
PLW13          0.16212000 W

F2 - Processing parameters
SI                  16384
SF            100.6177862 MHz
WDW                    EM
SSB       0
LB                   1.00 Hz
GB        0
PC                   1.20
```
